# Supplementary material for: Identification of novel genes with enriched expression in human intermediate progenitors reveals a key role for CDKN3 in cortical development
Source: Stem Cell Reports. 2026 Jul 9;21(8):103012. doi: 10.1016/j.stemcr.2026.103012 (PMC13476879; doi:10.1016/j.stemcr.2026.103012)
Supplement: Supplementary file 1 [file mmc1.pdf]

Stem Cell Reports, Volume *21*

Supplemental Information

Identification of novel genes with enriched expression in human intermediate progenitors reveals a key role for CDKN3 in cortical development

Pauline Antonie Ulmke, Martin N. Ivanov, Hoang Duy Nguyen, Linh Pham, Asisa Muchamedin, Ayman Alzu'bi, Lora V. Veleva, Tsvetomir Kachovski, Xiaoyi Mao, Krzysztof P. Lubieniecki, Emil Kovachev, Gavin J. Clowry, Anton B. Tonchev, Huu Phuc Nguyen, and Tran Tuoc

Supplemental Figures

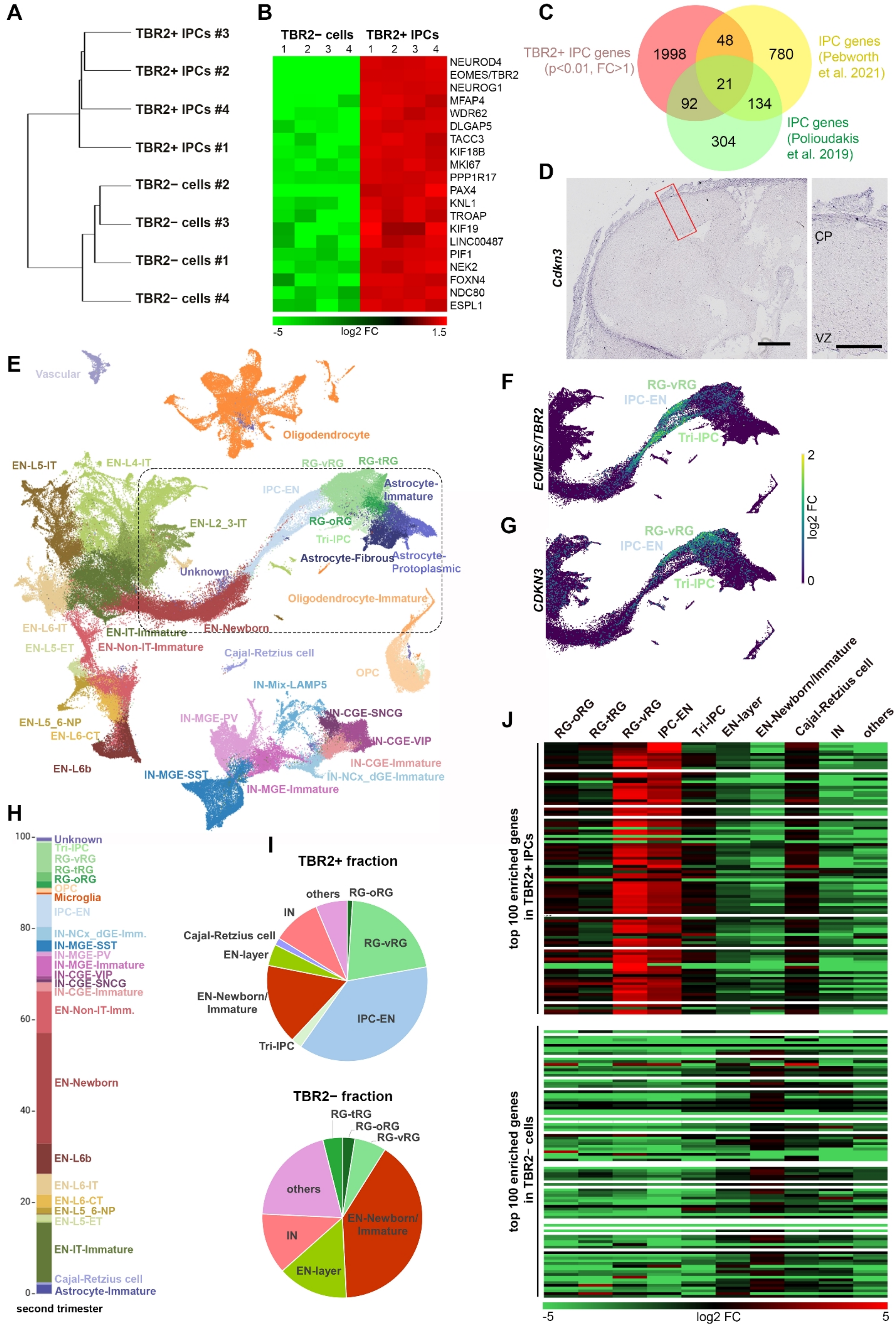

Figure S1. RNA-seq analysis of human TBR2+ IPCs and TBR2− nuclei in the context with scRNA-seq data.

(A) Cluster dendrogram analysis of RNA-seq for human TBR2+ and TBR2− sorted nuclei samples (*N* = 4 RNA-seq libraries per condition). **(B)** Heatmap showing the top 20 genes with high expression (enriched/upregulated) in TBR2+ nuclei and low expression in TBR2− nuclei. **(C)** Overlap of genes upregulated in hIPCs in this study (p-value < 0.01, log2FC > 1) and IPC genes identified in previous studies. **(D)** *In situ* hybridization for *Cdkn3* in the E14.5 mouse brain. Magnified cortical region is shown by a red box. **(E)** UMAP plots of the scRNA-seq data from Wang el al., 2025 showing the distribution of cell types from 38 human neocortical samples across different developmental stages. **(F, G)** UMAP plots of the scRNA-seq data from Wang el al. showing expression levels of *EOMES/TBR2* (F) and *CDKN3* (G). Expression values are displayed as log2-transformed normalized UMI counts, plotted relative to the mean expression across all cells in the dataset. **(H)** Cell composition of the second-trimester cortex (GW13–23) identified by Wang et al. **(I)** Pie chart showing cell type proportions of TBR2+ hIPCs and TBR2− cells after deconvolution with scRNA-seq data of the Wang et al. dataset. **(J)** Heatmap showing the top 100 genes with high expression in TBR2+ nuclei and the top 100 genes with high expression in TBR2− nuclei in cell types identified in the scRNA-seq from Wang el al. VZ, ventricular zone; CP, cortical plate; oRG, outer radial glia; tRG, truncated RG; vRG; ventricular RG, IPC-EN, intermediate progenitors for excitatory neurons; Tri-IPC, tripotent IPC; IT, intratelencephalic; L, layer; IN, interneurons; Imm., immature; IN-NCx_dGE-Immature, neocortex and dorsal ganglionic eminence-derived immature INs; CGE, caudal ganglionic eminence; SNCG, synuclein-gamma; VIP, vasoactive intestinal polypeptide; MGE, medial ganglionic eminence; PV, parvalbumin; SST, somatostatin; LAMP5, Lysosomal Associated Membrane Protein Family Member 5; OPC, oligodendrocyte precursor cells. Scale bars: 500 μm, 250 μm (higher magnification).

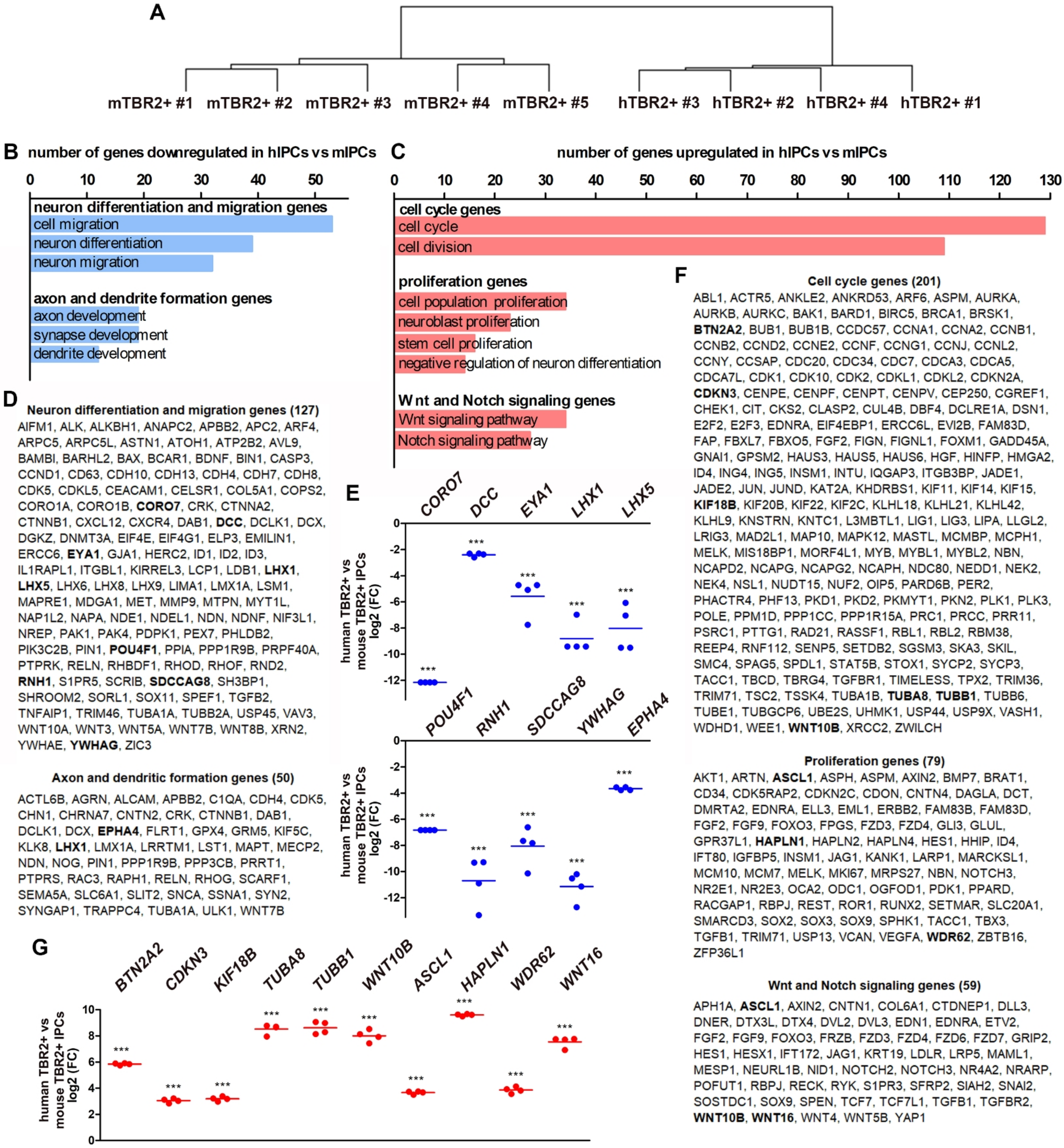

A
mTBR2+ #1
mTBR2+ #2
mTBR2+ #3
mTBR2+ #4
mTBR2+ #5
hTBR2+ #3
hTBR2+ #2
hTBR2+ #4
hTBR2+ #1
B
number of genes downregulated in hIPCs vs mIPCs
0
10
20
30
40
50
neuron differentiation and migration genes
cell migration
neuron differentiation
neuron migration
axon and dendrite formation genes
axon development
synapse development
dendrite development
C
number of genes upregulated in hIPCs vs mIPCs
0
10
20
30
40
50
60
70
80
90
100
110
120
130
cell cycle genes
cell cycle
cell division
proliferation genes
cell population proliferation
neuroblast proliferation
stem cell proliferation
negative regulation of neuron differentiation
Wnt and Notch signaling genes
Wnt signaling pathway
Notch signaling pathway
D
Neuron differentiation and migration genes (127)
AIFM1, ALK, ALKBH1, ANAPC2, APBB2, APC2, ARF4, ARPC5, ARPC5L, ASTN1, ATOH1, ATP2B2, AVL9, BAMBI, BARHL2, BAX, BCAR1, BDNF, BIN1, CASP3, CCND1, CD63, CDH10, CDH13, CDH4, CDH7, CDH8, CDK5, CDKL5, CEACAM1, CELSR1, COL5A1, COPS2, CORO1A, CORO1B, **CORO7**, CRK, CTNNA2, CTNNB1, CXCL12, CXCR4, DAB1, **DCC**, DCLK1, DCX, DGKZ, DNMT3A, EIF4E, EIF4G1, ELP3, EMILIN1, ERCC6, **EYA1**, GJA1, HERC2, ID1, ID2, ID3, IL1RAPL1, ITGBL1, KIRREL3, LCP1, LDB1, **LHX1**, **LHX5**, LHX6, LHX8, LHX9, LIMA1, LMX1A, LSM1, MAPRE1, MDGA1, MET, MMP9, MTPN, MYT1L, NAP1L2, NAPA, NDE1, NDEL1, NDN, NDNF, NIF3L1, NREP, PAK1, PAK4, PDPK1, PEX7, PHLDB2, PIK3C2B, PIN1, **POU4F1**, PPIA, PPP1R9B, PRPF40A, PTPRK, RELN, RHBDF1, RHOD, RHOF, RND2, **RNH1**, S1PR5, SCRIB, **SDCCAG8**, SH3BP1, SHROOM2, SORL1, SOX11, SPEF1, TGFB2, TNFAIP1, TRIM46, TUBA1A, TUBB2A, USP45, VAV3, WNT10A, WNT3, WNT5A, WNT7B, WNT8B, XRN2, YWHAE, **YWHAG**, ZIC3
Axon and dendritic formation genes (50)
ACTL6B, AGRN, ALCAM, APBB2, C1QA, CDH4, CDK5, CHN1, CHRNA7, CNTN2, CRK, CTNNB1, DAB1, DCLK1, DCX, **EPHA4**, FLRT1, GPX4, GRM5, KIF5C, KLK8, **LHX1**, LMX1A, LRRTM1, LST1, MAPT, MECP2, NDN, NOG, PIN1, PPP1R9B, PPP3CB, PRRT1, PTPRS, RAC3, RAPH1, RELN, RHOG, SCARF1, SEMA5A, SLC6A1, SLIT2, SNCA, SSNA1, SYN2, SYNGAP1, TRAPPC4, TUBA1A, ULK1, WNT7B
E
CORO7
DCC
EYA1
LHX1
LHX5
human TBR2+ vs mouse TBR2+ IPCs log2 (FC)
0
-2
-4
-6
-8
-10
-12
***
POU4F1
RNH1
SDCCAG8
YWHAG
EPHA4
F
Cell cycle genes (201)
ABL1, ACTR5, ANKLE2, ANKRD53, ARF6, ASPM, AURKA, AURKB, AURKC, BAK1, BARD1, BIRC5, BRCA1, BRSK1, **BTN2A2**, BUB1, BUB1B, CCDC57, CCNA1, CCNA2, CCNB1, CCNB2, CCND2, CCNE2, CCNF, CCNG1, CCNJ, CCNL2, CCNY, CCSAP, CDC20, CDC34, CDC7, CDCA3, CDCA5, CDCA7L, CDK1, CDK10, CDK2, CDKL1, CDKL2, CDKN2A, **CDKN3**, CENPE, CENPF, CENPT, CENPV, CEP250, CGREF1, CHEK1, CIT, CKS2, CLASP2, CUL4B, DBF4, DCLRE1A, DSN1, E2F2, E2F3, EDNRA, EIF4EBP1, ERCC6L, EVI2B, FAM83D, FAP, FBXL7, FBXO5, FGF2, FIGN, FIGNL1, FOXM1, GADD45A, GNAI1, GPSM2, HAUS3, HAUS5, HAUS6, HGF, HINFP, HMGA2, ID4, ING4, ING5, INSM1, INTU, IQGAP3, ITGB3BP, JADE1, JADE2, JUN, JUND, KAT2A, KHDRBS1, KIF11, KIF14, KIF15, **KIF18B**, KIF20B, KIF22, KIF2C, KLHL18, KLHL21, KLHL42, KLHL9, KNSTRN, KNTC1, L3MBTL1, LIG1, LIG3, LIPA, LLGL2, LRIG3, MAD2L1, MAP10, MAPK12, MASTL, MCMBP, MCPH1, MELK, MIS18BP1, MORF4L1, MYB, MYBL1, MYBL2, NBN, NCAPD2, NCAPG, NCAPG2, NCAPH, NDC80, NEDD1, NEK2, NEK4, NSL1, NUDT15, NUF2, OIP5, PARD6B, PER2, PHACTR4, PHF13, PKD1, PKD2, PKMYT1, PKN2, PLK1, PLK3, POLE, PPM1D, PPP1CC, PPP1R15A, PRC1, PRCC, PRR11, PSRC1, PTTG1, RAD21, RASSF1, RBL1, RBL2, RBM38, REEP4, RNF112, SENP5, SETDB2, SGSM3, SKA3, SKIL, SMC4, SPAG5, SPDL1, STAT5B, STOX1, SYCP2, SYCP3, TACC1, TBCD, TBRG4, TGFBR1, TIMELESS, TPX2, TRIM36, TRIM71, TSC2, TSSK4, TUBA1B, **TUBA8**, **TUBB1**, TUBB6, TUBE1, TUBGCP6, UBE2S, UHMK1, USP44, USP9X, VASH1, WDHD1, WEE1, **WNT10B**, XRCC2, ZWILCH
Proliferation genes (79)
AKT1, ARTN, **ASCL1**, ASPH, ASPM, AXIN2, BMP7, BRAT1, CD34, CDK5RAP2, CDKN2C, CDON, CNTN4, DAGLA, DCT, DMRTA2, EDNRA, ELL3, EML1, ERBB2, FAM83B, FAM83D, FGF2, FGF9, FOXO3, FPGS, FZD3, FZD4, GLI3, GLUL, GPR37L1, **HAPLN1**, HAPLN2, HAPLN4, HES1, HHIP, ID4, IFT80, IGFBP5, INSM1, JAG1, KANK1, LARP1, MARCKSL1, MCM10, MCM7, MELK, MKI67, MRPS27, NBN, NOTCH3, NR2E1, NR2E3, OCA2, ODC1, OGFOD1, PDK1, PPARD, RACGAP1, RBPJ, REST, ROR1, RUNX2, SETMAR, SLC20A1, SMARCD3, SOX2, SOX3, SOX9, SPHK1, TACC1, TBX3, TGFB1, TRIM71, USP13, VCAN, VEGFA, **WDR62**, ZBTB16, ZFP36L1
Wnt and Notch signaling genes (59)
APH1A, **ASCL1**, AXIN2, CNTN1, COL6A1, CTDNEP1, DLL3, DNER, DTX3L, DTX4, DVL2, DVL3, EDN1, EDNRA, ETV2, FGF2, FGF9, FOXO3, FRZB, FZD3, FZD4, FZD6, FZD7, GRIP2, HES1, HESX1, IFT172, JAG1, KRT19, LDLR, LRP5, MAML1, MESP1, NEURL1B, NID1, NOTCH2, NOTCH3, NR4A2, NRARP, POFUT1, RBPJ, RECK, RYK, S1PR3, SFRP2, SIAH2, SNAI2, SOSTDC1, SOX9, SPEN, TCF7, TCF7L1, TGFB1, TGFBR2, **WNT10B**, **WNT16**, WNT4, WNT5B, YAP1
G
BTN2A2
CDKN3
KIF18B
TUBA8
TUBB1
WNT10B
ASCL1
HAPLN1
WDR62
WNT16
human TBR2+ vs mouse TBR2+ IPCs log2 (FC)
10
8
6
4
2
0
***

Figure S2. Upregulation of proliferation and cell cycle genes and downregulation of neuronal differentiation genes in hIPCs versus mIPCs.

(**A**) Cluster dendrogram analysis of RNA-seq for mouse TBR2+ sorted nuclei (*N* = 5 RNA-seq libraries per condition) and human TBR2+ sorted nuclei (*N* = 4 RNA-seq libraries per condition). (**B, C**) Gene Ontology analyses of genes downregulated in hIPCs versus mIPCs (B), and genes upregulated in hIPCs versus mIPCs (C). **(D, E)** List of genes downregulated in hIPCs versus mIPCs that functionally fall under neuron differentiation and migration and axon and dendritic formation (D) and the expression levels of selected genes (highlighted bold in the adjoining gene list) as identified in the individual samples of hIPCs versus mIPCs (E). (**F, G**) List of genes upregulated in hIPCs versus mIPCs that functionally fall under cell cycle, proliferation, and Wnt/Notch signalling, respectively (F) and the expression levels of selected genes (highlighted bold in the adjoining gene list) as identified in the individual samples of hIPCs versus mIPCs (G). ***P<0.001.

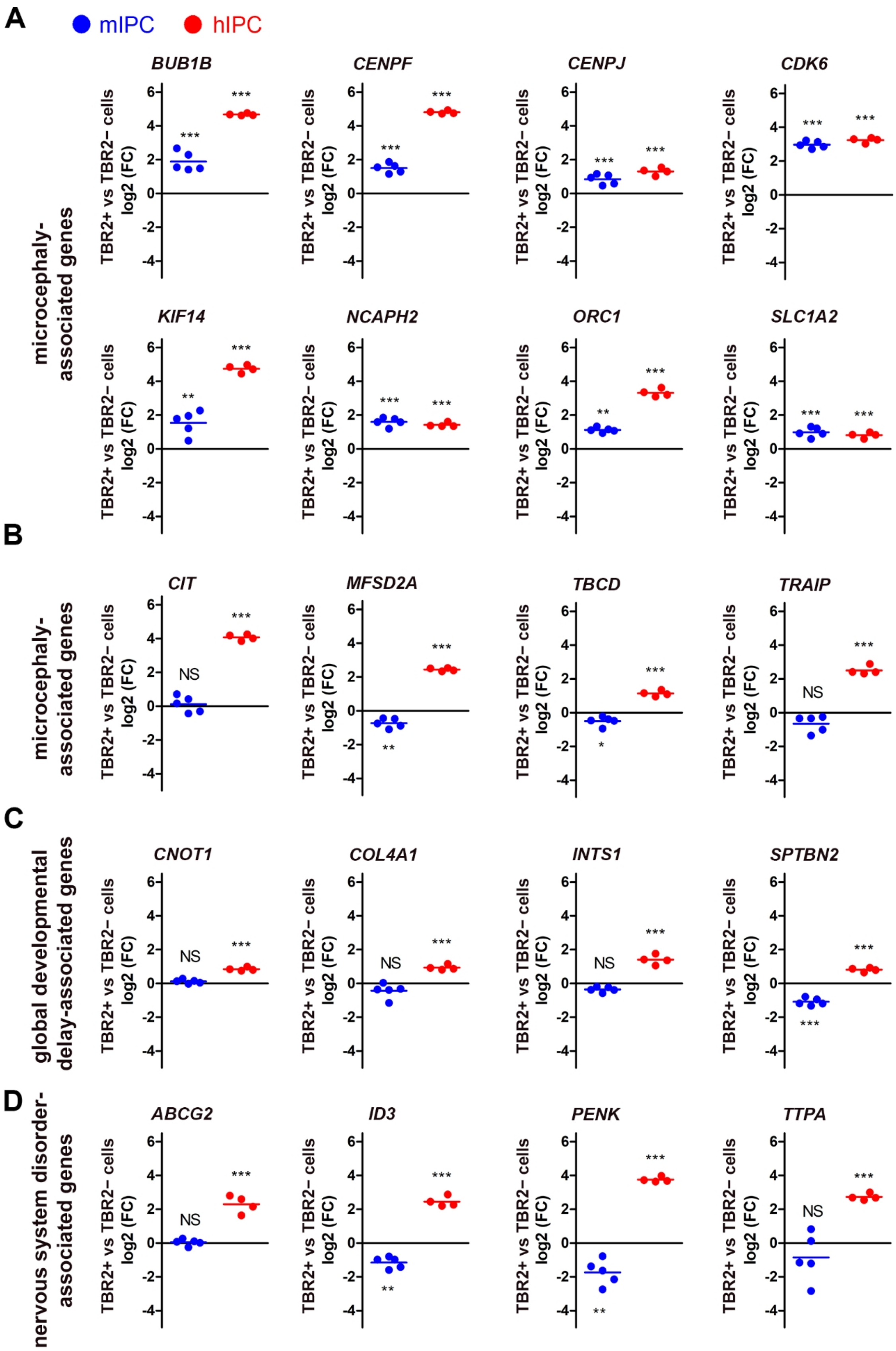

A
mIPC
hIPC
microcephaly-associated genes
BUB1B
CENPF
CENPJ
CDK6
KIF14
NCAPH2
ORC1
SLC1A2
TBR2+ vs TBR2− cells log2 (FC)
B
microcephaly-associated genes
CIT
MFSD2A
TBCD
TRAIP
C
global developmental delay-associated genes
CNOT1
COL4A1
INTS1
SPTBN2
D
nervous system disorder-associated genes
ABCG2
ID3
PENK
TTPA
NS

Figure S3. hIPC genes involved in diseases linked to brain development.

(A-D) The expression levels of selected genes as identified in the individual samples in RNA-seq of mIPCs and hIPCs presenting evolutionary conserved IPC genes linked to primary microcephaly (A) and human-specific IPC-enriched genes associated with primary microcephaly (B), global developmental delay (C) and nervous system disorders (D). *N* = 5 RNA-seq libraries per condition for mIPCs and *N* = 4 RNA-seq libraries per condition for hIPCs. *P<0.05, **P<0.01, ***P<0.001; NS, not significant.

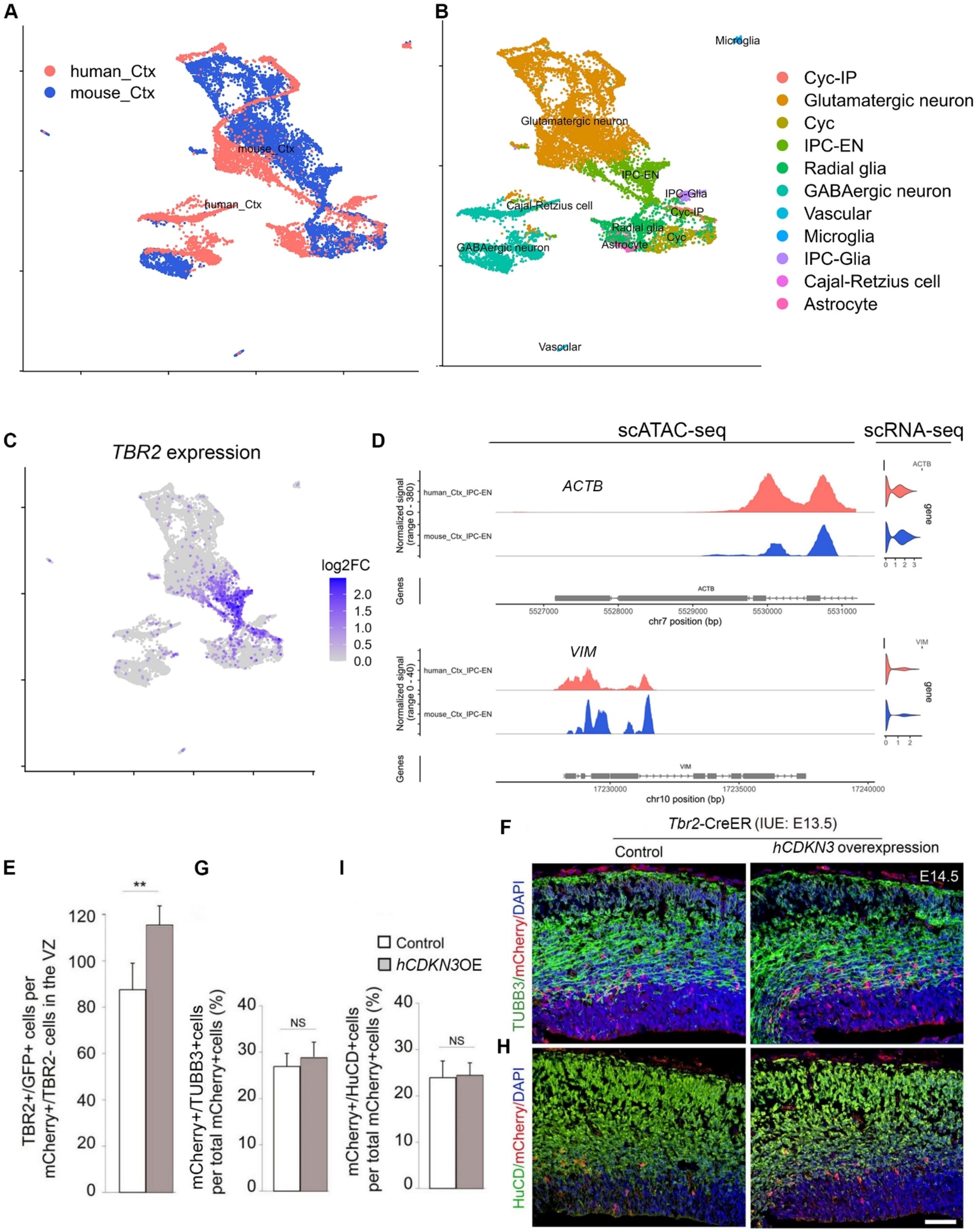

A
human_Ctx
mouse_Ctx
B
Microglia
Glutamatergic neuron
IPC-EN
IPC-Glia
Cyc-IP
Cajal-Retzius cell
Radial glia
Cyc
Astrocyte
GABAergic neuron
Vascular
Cyc-IP
Glutamatergic neuron
Cyc
IPC-EN
Radial glia
GABAergic neuron
Vascular
Microglia
IPC-Glia
Cajal-Retzius cell
Astrocyte
C
TBR2 expression
log2FC
2.0
1.5
1.0
0.5
0.0
D
scATAC-seq
scRNA-seq
ACTB
Normalized signal (range 0 - 380)
human_Ctx_IPC-EN
mouse_Ctx_IPC-EN
Genes
chr7 position (bp)
VIM
Normalized signal (range 0 - 40)
chr10 position (bp)
E
TBR2+/GFP+ cells per mCherry+/TBR2- cells in the VZ
**
G
mCherry+/TUBB3+cells per total mCherry+cells (%)
NS
I
Control
hCDKN3OE
mCherry+/HuCD+cells per total mCherry+cells (%)
NS
F
Tbr2-CreER (IUE: E13.5)
Control
hCDKN3 overexpression
E14.5
TUBB3/mCherry/DAPI
H
HuCD/mCherry/DAPI

Figure S4. Cross-species single-cell integration and effects of CDKN3 overexpression in mouse IPCs.

(A, B) UMAP visualization of integrated single-cell multiome datasets from developing human and mouse cortex (Wang et al., 2025; Liu et al., 2025), showing combined cells from both species following cross-species integration, colored by species (A) or by annotated cell clusters (B). **(C)** Feature plot showing *TBR2* expression across integrated clusters. **(D)** Cross-species single-cell ATAC-seq analysis showing chromatin accessibility at the *ACTB* and the *VIM* locus in mouse and human radial glia and IPC-EN together with expression levels. **(E)** Bar graph showing quantitative analysis of eGFP and mCherry co-expressing cells in E15.5 control and *hCDKN3*OE cortices (*N* = 6 images from 3 independent embryos per condition). **(F, H)** Double immunohistochemical analysis with antibodies that specifically mark mCherry and neuronal markers TUBB3 (F) or HuC/HuD (H) in the E14.5 *Tbr2*CreER mouse cortex, 24 h after electroporation with an empty mCherry-eGFP-only plasmid as control or *hCDKN3* overexpression plasmid. **(G, I)** Bar graphs showing quantitative analysis of TUBB3 (G) or HuC/HuD (I) and mCherry co-expressing neurons in E14.5 control and *hCDKN3*OE cortices (*N* = 6 images from 3 independent embryos per condition). Cyc-IP, cycling intermediate progenitor cells; IPC-EN, intermediate progenitors generating excitatory neurons; Cyc, cycling progenitors; IPC-Glia, intermediate progenitors generating glial cells. Scale bar: 100 μm. *$P<0.05$, **$P<0.01$, ***$P<0.001$; NS, not significant.

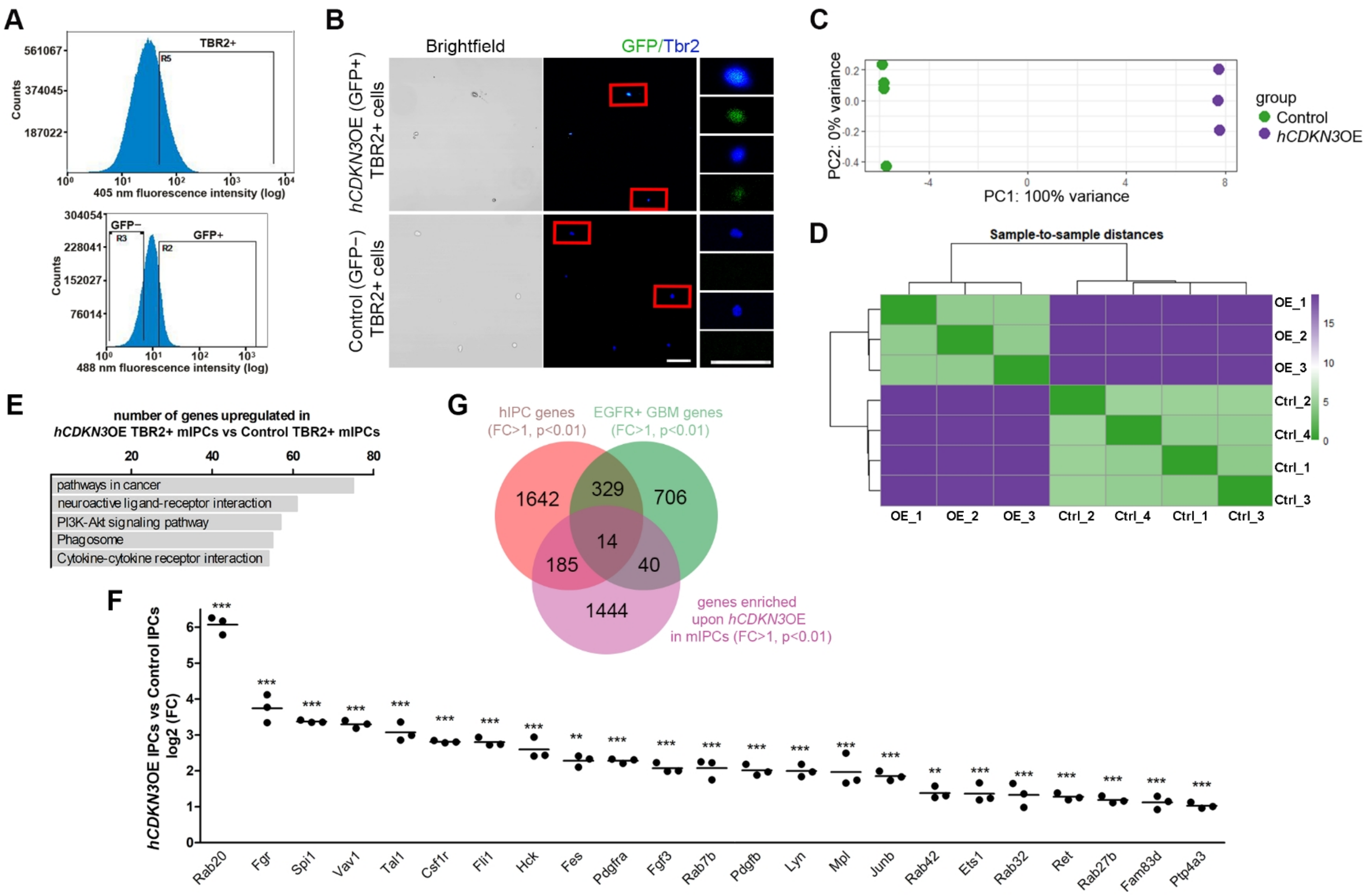

A
TBR2+
R5
Counts
405 nm fluorescence intensity (log)
GFP−
R3
GFP+
R2
488 nm fluorescence intensity (log)
B
Brightfield
GFP/Tbr2
hCDKN3OE (GFP+) TBR2+ cells
Control (GFP−) TBR2+ cells
C
PC2: 0% variance
PC1: 100% variance
group
Control
hCDKN3OE
D
Sample-to-sample distances
OE_1
OE_2
OE_3
Ctrl_2
Ctrl_4
Ctrl_1
Ctrl_3
E
number of genes upregulated in hCDKN3OE TBR2+ mIPCs vs Control TBR2+ mIPCs
pathways in cancer
neuroactive ligand-receptor interaction
PI3K-Akt signaling pathway
Phagosome
Cytokine-cytokine receptor interaction
G
hIPC genes (FC>1, p<0.01)
EGFR+ GBM genes (FC>1, p<0.01)
genes enriched upon hCDKN3OE in mIPCs (FC>1, p<0.01)
1642
329
706
14
185
40
1444
F
hCDKN3OE IPCs vs Control IPCs log2 (FC)
Rab20
Fgr
Spi1
Vav1
Tal1
Csf1r
Fli1
Hck
Fes
Pdgfra
Fgf3
Rab7b
Pdgfb
Lyn
Mpl
Junb
Rab42
Ets1
Rab32
Ret
Rab27b
Fam83d
Ptp4a3

Figure S5. Validation and transcriptomic analysis of CDKN3-overexpressing IPCs.

(**A**) Representative FACS plots showing sorting gates for TBR2 (405 nm) and GFP (488 nm) signals used to isolate *hCDKN3*-overexpressing (OE) and control TBR2+ IPCs (Ctrl). (**B**) Representative images of post-sorted cell suspensions from *in utero* electroporated mouse cortex labeled with TBR2 antibody. (**C**) PCA plot showing transcriptomic variance between *hCDKN3*OE and control samples. **(D)** Cluster dendrogram analysis of RNA-seq for sorted *hCDKN3*OE TBR2+ cells and control TBR2+ cells ($N \geq 3$ technical replicates for both conditions). **(E)** KEGG pathway enrichment analysis of genes upregulated in *hCDKN3*OE mIPCs vs control mIPCs (p-value < 0.01, log2FC > 1). **(F)** Expression levels of selected proto-oncogenes in *hCDKN3*OE mIPCs versus control mIPCs. **(G)** Overlap of genes upregulated in hIPCs, in EGFR+ GBM and in mIPCs with *hCDKN3*OE (p-value < 0.01, log2FC > 1). Scale bar: 100 µm. *P<0.05, **P<0.01, ***P<0.001; NS, not significant.

Supplemental Table Legends (separate files)

Table S1. Differential gene expression between human TBR2+ IPCs and TBR2− nuclei (related to Fig. 1 and S1)

Table S2. Overlap between mIPC and hIPC genes including Gene Ontology categories (related to Fig. 2 and S2)

Table S3. Chromatin, epigenetic and TF genes upregulated in hIPCs (related to Fig. 3)

Table S4. hIPC genes associated to diseases (related to Fig. 4 and S3)

Table S5. 266 human-specific top-enriched IPC genes and their categories (related to Fig. 5)

Table S6. Differential gene expression between *hCDKN3*OE mIPCs and Control mIPCs including Gene Ontology categories (related to Fig. 7 and S5)

Table S7. Statistical analyses

Table S8. List of primers
