# Supplementary material for: Identification of novel genes with enriched expression in human intermediate progenitors reveals a key role for CDKN3 in cortical development
Source: Stem Cell Reports. 2026 Jul 9;21(8):103012. doi: 10.1016/j.stemcr.2026.103012 (PMC13476879; doi:10.1016/j.stemcr.2026.103012)
Supplement: Supplementary file 2 [file mmc10.pdf]

STEM CELL REPORTS
Article
ISSCR
OPEN ACCESS

Identification of novel genes with enriched expression in human intermediate progenitors reveals a key role for CDKN3 in cortical development

Pauline Antonie Ulmke,[1,6,*] Martin N. Ivanov,[2,3,6] Hoang Duy Nguyen,[1] Linh Pham,[1] Asisa Muchamedin,[1] Ayman Alzu'bi,[5] Lora V. Veleva,[3] Tsvetomir Kachovski,[4] Xiaoyi Mao,[1] Krzysztof P. Lubieniecki,[1] Emil Kovachev,[4] Gavin J. Clowry,[5] Anton B. Tonchev,[2,3] Huu Phuc Nguyen,[1] and Tran Tuoc[1,7,*]
[1]Department of Human Genetics, Ruhr University Bochum, Bochum, Germany
[2]NutriLect Research Group, Research Institute, Medical University-Varna, Varna, Bulgaria
[3]Department of Anatomy and Cell Biology, Medical University-Varna, Varna, Bulgaria
[4]Specialized Hospital for Obstetrics and Gynecology "Prof. Dimitar Stamatov"–Varna, Medical University-Varna, Varna, Bulgaria
[5]Newcastle University Biosciences Institute, Newcastle University, Newcastle upon Tyne, UK
[6]These authors contributed equally
[7]Lead contact
*Correspondence: pauline.ulmke@ruhr-uni-bochum.de (P.A.U.), tran.tuoc@ruhr-uni-bochum.de (T.T.)
https://doi.org/10.1016/j.stemcr.2026.103012

SUMMARY

The evolutionary expansion of the human neocortex relies on species-specific features of intermediate progenitor cells (IPCs), including enhanced proliferation and neuronal production. The transcriptomic differences in IPCs underlying their differential capacity across species remain elusive. To identify the transcriptional signature of human IPCs (hIPCs), we isolated TBR2-positive IPCs from developing human neocortex. A comparative genome-wide expression analysis of IPC transcriptional profiles from human and mouse outlined genes preferentially expressed in hIPCs encoding key factors of cell signaling, transcriptional regulation, and proliferation. Mutations in several hIPC-specific genes were linked to cortical malformations and brain tumors. Functional experiments involving hIPC-specific overexpression of *CDKN3* in developing mouse cortex validated the pivotal role of *CDKN3* in IPC proliferation and neurogenesis, and supported its identification as a key determinant of hIPC biogenesis. Our findings offer new insights into the molecular features of hIPCs underlying their capacity to mediate the evolutionary expansion of the human neocortex.

INTRODUCTION

In the developing cerebral cortex, intermediate progenitor cells (IPCs), which express the T-box transcription factor (TF) TBR2, function as critical transit-amplifying cells, producing most cortical neurons (Haubensak et al., 2004; Hevner, 2019; Kowalczyk et al., 2009; Miyata et al., 2004; Noctor et al., 2004). Our recent study (Ulmke et al., 2021) characterized the molecular profile of IPCs in the developing mouse cortex. A genome-wide comparison of transcriptional profiles between TBR2+ mouse IPCs (mIPCs) and TBR2− cells uncovered key regulatory elements associated with cell cycle progression, chromatid segregation, transcriptional control, and cell signaling mechanisms. Notably, mutations in several IPC-related genes in humans lead to intellectual disabilities and diverse cortical malformations, such as microcephaly and corpus callosum agenesis.

Although IPCs in both rodents and humans originate from multipotent radial glial cells (RGCs) in the developing neocortex, species-specific features of IPCs have been observed. For instance, most IPCs in the mouse cortex do not express RGC markers such as SOX2 and PAX6, whereas nearly half of human TBR2+ IPCs are immunoreactive for these markers (Fietz et al., 2010; Hansen et al., 2010). Additionally, while most IPCs are direct neurogenic progenitors in rodents, the majority of human IPCs (hIPCs) can self-amplify via symmetric proliferative divisions prior to terminally differentiating into neurons (Lui et al., 2011). The complex enlargement of the human neocortex is thought to be an evolutionary adaptation, driven by the extensive growth of neuronal numbers resulting from the enhanced proliferative capacity of human neural progenitors, particularly IPCs (Dehay et al., 2015; Lui et al., 2011; Nonaka-Kinoshita et al., 2013; Stahl et al., 2013). Thus, identifying molecular factors that sustain the proliferative potential of hIPCs in the subventricular zone (SVZ) niche is crucial for understanding cortical development. Elucidating transcriptional patterns that control the generation and maintenance of hIPCs could enhance our comprehension of cortical development and aid in the development of protocols for culturing hIPCs or generating hIPCs through reprogramming of other cell sources. Furthermore, understanding the molecular features of hIPCs can illuminate the genetic underpinnings of neurological disorders resulting from defective hIPC genesis.

Single-cell RNA sequencing (scRNA-seq) analyses have been employed to investigate the molecular identities of cell types in the developing cortex, including IPCs, enabling their characterization in the developing cortex of both mouse (Kawaguchi et al., 2008; Li et al., 2020; Loo et al., 2019; Telley et al., 2016) and human (Fan

Published by Elsevier Inc. on behalf of International Society for Stem Cell Research.
This is an open access article under the CC BY license (http://creativecommons.org/licenses/by/4.0/).

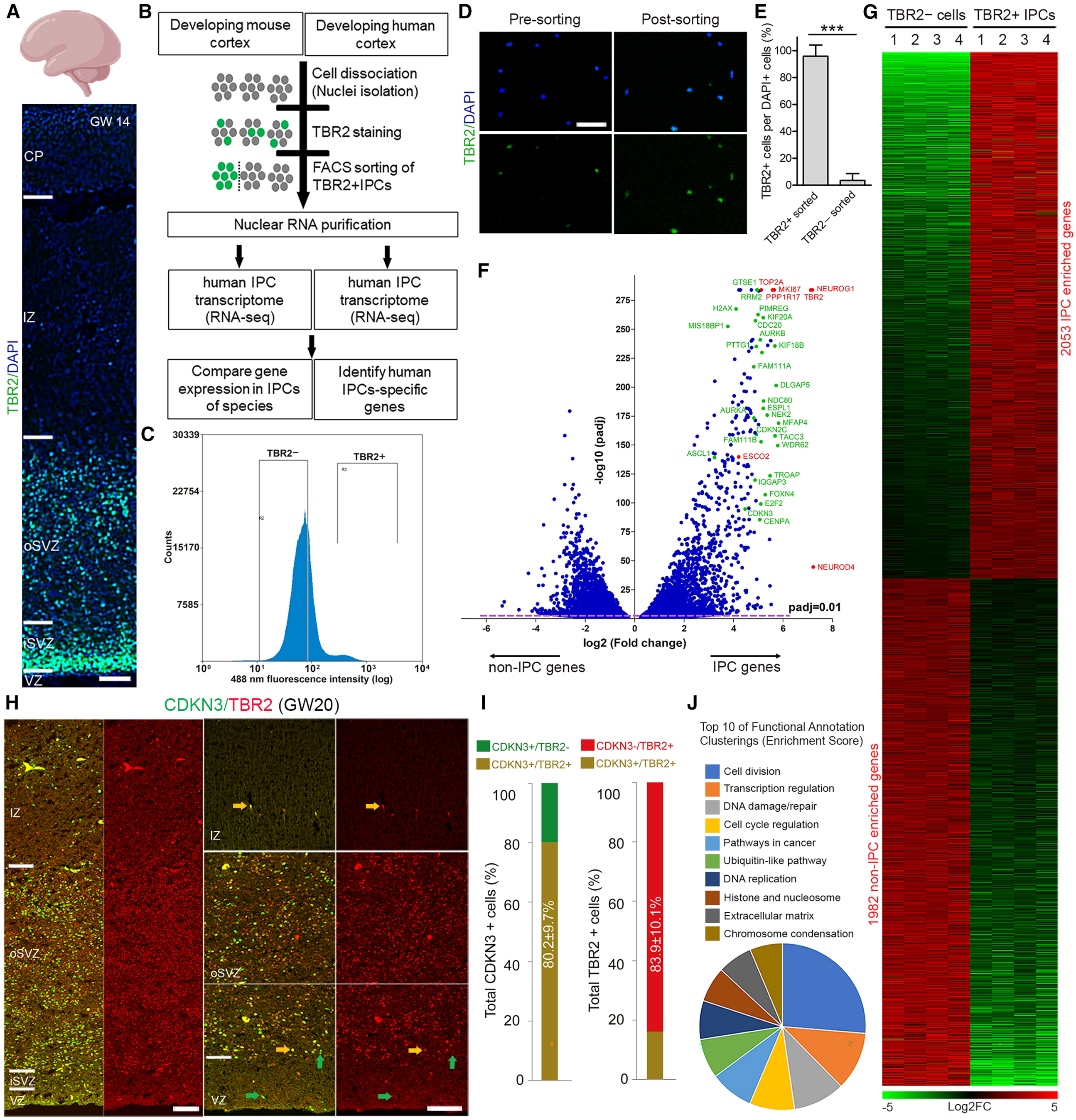

Figure 1. Cell sorting and gene expression profiling of human TBR2+ IPCs

(A) Coronal section of human cortex at gestational week 14, immunostained with TBR2 antibody. DAPI was used to label nuclei.

(B) An illustration of the experimental design used to purify TBR2-labled IPCs followed by RNA-seq analysis to compare the transcriptional profile of IPCs in humans.

(C) Representative plot shows sorting gates for TBR2+ and TBR2— nuclei from human cortex.

(D) Representative images of pre- and post-sorted nuclei suspensions from human cortex labeled with TBR2 antibody. DAPI was used to label nuclei.

(E) Bar graphs show quantitative analysis of the proportion of TBR2+ nuclei among DAPI+ nuclei after FACS ($N = 3$ images per condition).

(legend continued on next page)

et al., 2018; Li et al., 2018; Nowakowski et al., 2017; Pollen et al., 2015; Zhong et al., 2018). Nevertheless, the limitations of low-read scRNA-seq pose challenges in accurately profiling gene expression, especially for those genes with low or moderate expression. Comparative transcriptome analyses of isolated cell populations have provided additional molecular insights into cortical cell types (Albert et al., 2017; Amamoto et al., 2020; Arlotta et al., 2005; Molyneaux et al., 2015; Pinto et al., 2008; Ulmke et al., 2021).

In this study, we utilized an antibody to mark intranuclear TBR2 in single-nucleus suspensions purified from developing human neocortex procured at the peak of neurogenesis at gestational weeks (GWs) 14–17 (Andrews and Pearson, 2024; Charvet, 2020; Zarzor et al., 2023) and sorted the TBR2+ nuclei (considered hIPC derived) from the TBR2− nuclei (considered non-hIPC derived). The expression profiles of hIPC-upregulated genes were determined via RNA-seq. We then compared the transcriptome of hIPCs with that of mIPCs, at embryonic day (E) 16.5, as previously defined (Ulmke et al., 2021). In mice, peak cortical neurogenesis occurs around E15.5–16.5 (Andrews and Pearson, 2024; Qian et al., 2000). Based on these developmental trajectories, GW14–17 in humans aligns with approximately E16.5 in mice in terms of neurogenic activity. The comparative genome-wide expression analysis revealed hIPC-specific genes involved in cell signaling, transcriptional regulation, and proliferation. Many of the identified hIPC-specific genes were linked to intellectual disability, cortical malformations, and brain tumors. Among these genes, cyclin-dependent kinase inhibitor 3 (*CDKN3*) was selected for confirmatory studies. *CDKN3* is among the top 100 most upregulated genes in hIPCs and has a well-established role in proliferation and cell cycle regulation. However, its function in IPCs has not been previously described, making it a strong candidate as a key regulator of hIPC proliferation. mIPC-specific ectopic expression of *CDKN3* in the developing mouse cortex specifically promoted IPC proliferation and neurogenesis, and RNA-seq analysis of *CDKN3*-overexpressing mIPCs revealed the upregulation of the cell cycle regulators *Ccnb1* and *Cdk1*, validating the identification of a key hIPC-specific factor in cortical neurogenesis throughout evolution.

RESULTS

Sorting and transcriptome analysis of IPCs in the developing human cortex

To investigate the transcriptomic profiles of TBR2+ IPCs and TBR2− cells in the developing human cortex (Figure 1A), we purified nuclei and applied a protocol combining intranuclear immunofluorescent staining with fluorescence-activated cell sorting (FACS) (Figure 1B). Single-nucleus suspensions isolated from GW 14 and 17 human cortices were sorted using the TBR2 antibody for labeling (Sakib et al., 2021). Gating parameters were optimized to selectively isolate TBR2+ IPC and TBR2− nuclei (Figure 1C). 96% ± 8% of sorted nuclei in suspensions were immunoreactive for TBR2 using the same antibody (Figures 1D and 1E).

To elucidate gene-regulatory differences between the purified cell populations, each sorted cell population was divided into four aliquots, and four independently prepared RNA-seq libraries were generated per condition (Figure S1A). Differential gene expression analysis using DESeq2 identified 2053 genes upregulated in human (h) IPCs and 1982 genes upregulated in non-IPCs that exhibited significant expression differences between these two cell populations (p adj <0.01, log2-fold change (FC) > 1.0, Figures 1F and 1G; Table S1). Consistently, canonical IPC genes, which are consistently and specifically associated with the identity of IPCs across multiple studies, exhibited high expression in the IPC population, aligning with *in vivo* expression profiles (Figure 1F in red; Figure S1B).

To identify novel IPC-upregulated genes, we compared our IPC gene list with published single-cell RNA-seq datasets from the developing human cortex (Fan et al., 2018; Li et al., 2018; Nowakowski et al., 2017; Pebworth et al., 2021; Polioudakis et al., 2019; Pollen et al., 2015; Zhong et al., 2018). We observed considerable overlap with previously reported IPC gene sets (Figure S1C). Notably, 1998 of the 2159 IPC-upregulated genes in our dataset had not been previously annotated as IPC-specific, highlighting a substantial number of novel candidate genes (Figure 1F, showing selected novel IPC-upregulated genes in green). The IPC-specific upregulated genes were categorized into

(F and G) Volcano plot (F) and heatmap (G) showing the upregulation of IPC and non-IPC genes in corresponding sorted cell populations (N = 4 independently prepared RNA-seq libraries per condition). Selected genes with expression at high levels and high significance are labeled in red (previously known) or green (novel) (G).

(H) Double immunohistochemical analysis with antibodies that specifically mark CDKN3 and TBR2 in the human cortex at gestational week (GW) 20. The six images on the right side represent higher magnifications of cortical areas from images on the left side.

(I) Quantitative analysis of the proportions of cells co-expressing CDKN3 and TBR2 in GW20 human cortex (N = 4 images from 2 independent embryos (2 images per embryo)).

(J) Proportions of the enrichment score of the top 10 functional annotation clusters in human IPCs. VZ, ventricular zone; iSVZ, inner subventricular zone; oSVZ, outer subventricular zone; IZ, intermediate zone. Scale bars, 100 μm.

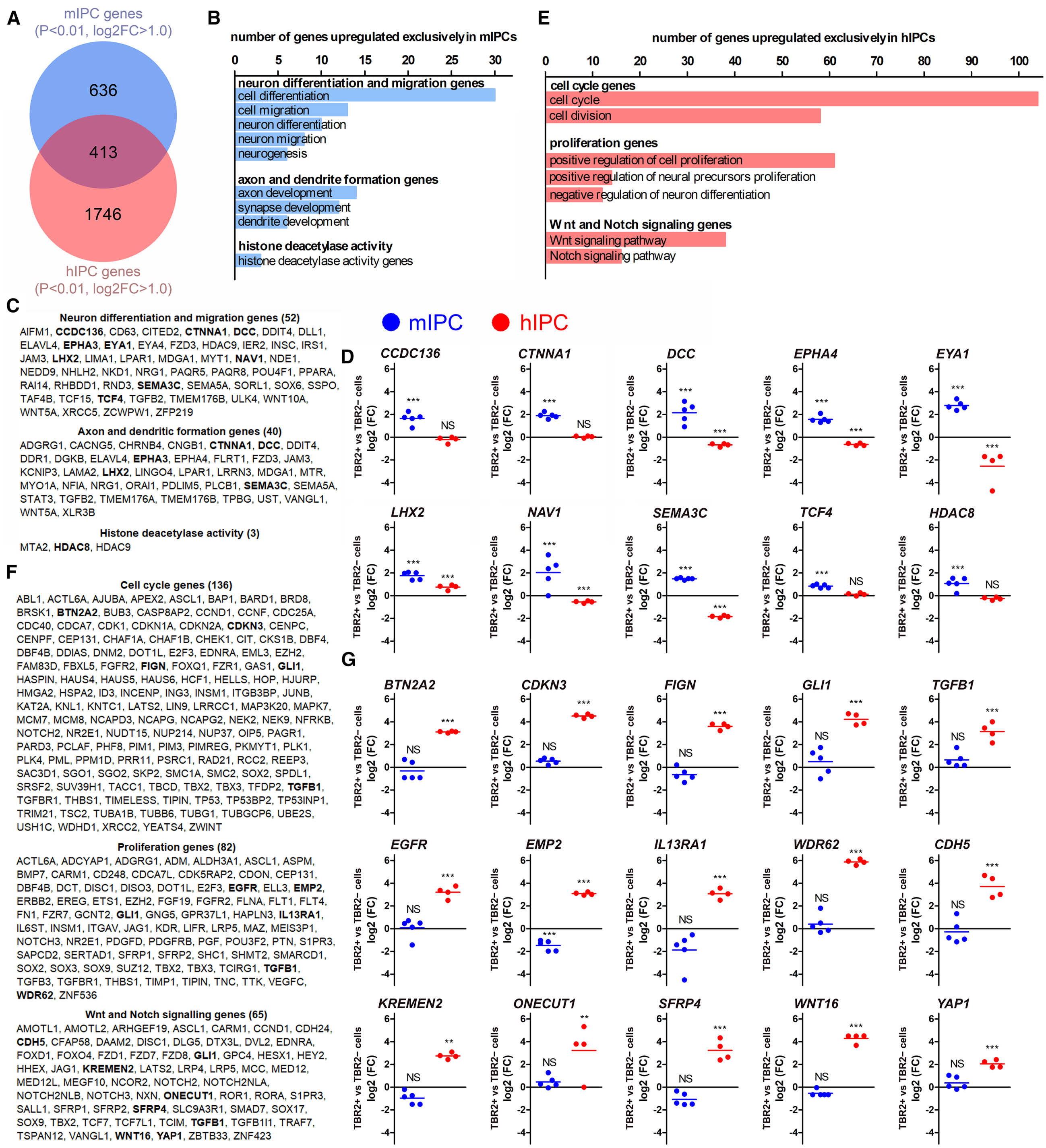

Figure 2. Upregulation of neuronal differentiation genes in mIPCs and upregulation of proliferation and cell cycle genes in hIPCs
(A) Overlap of genes upregulated in mIPCs and hIPCs (*p* value <0.01, log2FC > 1, mIPCs: *N* = 5 independently prepared RNA-seq libraries per condition; hIPCs: *N* = 4 independently prepared RNA-seq libraries per condition).
(B and C) Graphical representation of gene ontology analysis of 636 genes upregulated exclusively in mIPCs (B).
(C and D) List of genes exclusively upregulated in mIPCs that functionally fall under neuron differentiation and migration, axon and dendritic formation, and histone deacetylase activity, respectively (C), and the expression levels of selected genes (highlighted bold in the adjoining gene list) as identified in the individual samples in RNA-seq of mIPCs and hIPCs (D).

(legend continued on next page)

distinct functional groups (Figures 1J; Table S1) and their significance is further explored in subsequent sections.

To confirm the expression patterns of identified hIPC-upregulated genes, *in situ* hybridization (ISH) and immunohistochemical (IHC) analyses validated their expression in the SVZ of the developing human cortex (Figure 1H, see also Figures 3D and 3F; Figures 5L–5Z), corresponding to their expression in RNA-seq (Table S1). E.g., high levels of CDKN3 were observed in the SVZ of the developing human cortex (Figures 1H and 1I). CDKN3 protein was predominantly found in the proliferative region of the developing human cortex, most notably within the outer SVZ (Figure 1H). Quantification showed that a large proportion of CDKN3+ cells co-expressed the IPC marker TBR2, whereas only a subpopulation of TBR2+ IPCs expressed CDKN3 (Figure 1I). In the developing mouse cortex, the expression of *Cdkn3* was not detectable (Figure S1D). Consistently, scRNA-seq data from the E12.5 mouse telencephalon did not show the upregulation of *Cdkn3* transcripts in either apical or basal progenitors (Tables 7 and S12 of Moreau et al., 2021). This demonstrated that CDKN3 exhibits a species-specific expression pattern with a strong expression in human SVZ, thus raising the possibility that CDKN3 plays an important role in species-specific hIPC expansion in humans.

As an additional quality control, the RNA-seq data were compared to publicly available scRNA-seq data from 38 human neocortical samples spanning various developmental stages (Figure S1E; Wang et al., 2025). This comparison confirmed that *EOMES/TBR2* expression is highly enriched in IPCs (Figure S1F). *CDKN3* is expressed in both IPCs and ventricular radial glia (vRG) (Figure S1G). Notably, its expression is confined to a subpopulation of IPCs, potentially marking a subset of highly proliferative IPCs.

Cell-type composition analysis of the second-trimester cortex (GW13–23) confirmed the dominance of RGCs, IPCs, excitatory neurons (ENs), interneurons (INs), and oligodendrocyte/astrocyte progenitors (Figure S1H). To further characterize IPC identity, we performed deconvolution on the bulk RNA-seq data of TBR2+ and TBR2− replicates, leveraging cell type-specific expression profiles from the Wang et al. dataset (Figure S1I). Both sorted fractions represent heterogeneous populations, reflecting the developmental continuum of cortical lineages as well as inherent limitations of selection based on a single marker. While the TBR2+ fraction was enriched for intermediate progenitors for excitatory neurons (IPC-ENs) and vRG populations, the TBR2− fraction was dominated by excitatory neuronal subtypes, consistent with effective enrichment despite expected cellular heterogeneity. Accordingly, our analyses focus on relative enrichment between TBR2+ and TBR2− populations rather than absolute cellular purity. Furthermore, expression levels of the top 100 upregulated genes in TBR2+ IPCs and TBR2− cells were analyzed across different cortical cell populations (Wang et al., 2025) (Figure S1J). TBR2+ IPCs from this study most closely resemble IPC-ENs and vRGs, while the TBR2− population exhibits the highest similarity to multiple EN and IN subtypes.

Species-specific features of IPC gene expression program

In our previous study, we analyzed the gene expression profiles of TBR2+ mIPCs and TBR2− cells at embryonic day 16.5 (E16.5) during murine cortical development (Ulmke et al., 2021). To identify species-specific features in the IPC transcriptome, we compared the list of 1049 genes upregulated in mIPCs (p value <0.01, log2FC > 1) with 2159 genes upregulated in hIPCs (p value <0.01, log2FC > 1, Figure 2A; Table S2). This comparison revealed an overlap of 413 genes expressed in both mIPCs and hIPCs, including all canonical IPC genes, highlighting their evolutionary conservation from mouse to human. Additionally, 636 genes were exclusively upregulated in mIPCs, while 1746 genes were exclusive to hIPCs (Figure 2A; Table S2).

Gene ontology (GO) analysis of the 636 genes exclusively upregulated in mIPCs underscored the predominantly neurogenic nature of mIPCs, revealing the upregulation of genes involved in neuronal differentiation, as well as those implicated in neuronal migration, axon, and dendrite formation (Figures 2B–2D). Notably, genes encoding histone deacetylases (HDACs) such as MTA2, HDAC8, and HDAC9 were prominently expressed in mIPCs, consistent with reports of low histone acetylation levels in these cells (Kerimoglu et al., 2021).

Conversely, reflecting the high proliferative capacity of hIPCs, which undergo symmetric proliferative divisions before differentiating into mature cells (Lui et al., 2011), a large number of cell cycle and proliferation genes were upregulated in hIPCs (Figures 2E–2G). Moreover, genes associated with proliferation-linked signaling pathways such as WNT and NOTCH were also notably upregulated in hIPCs (Figures 2E–2G).

Additionally, comparing the differently expressed genes between mouse and human TBR2+ IPCs directly also revealed a robust upregulation of cell cycle and

(E) Graphical representation of Gene Ontology analysis of 1746 genes upregulated exclusively in hIPCs (F, G) List of genes exclusively upregulated in hIPCs that functionally fall under cell cycle, proliferation and Wnt/Notch signaling, respectively (F) and the expression levels of selected genes (highlighted bold in the adjoining gene list) as identified in the individual samples in RNA-seq of mIPCs and hIPCs (G). *p < 0.05, **p < 0.01, and ***p < 0.001; NS, not significant.

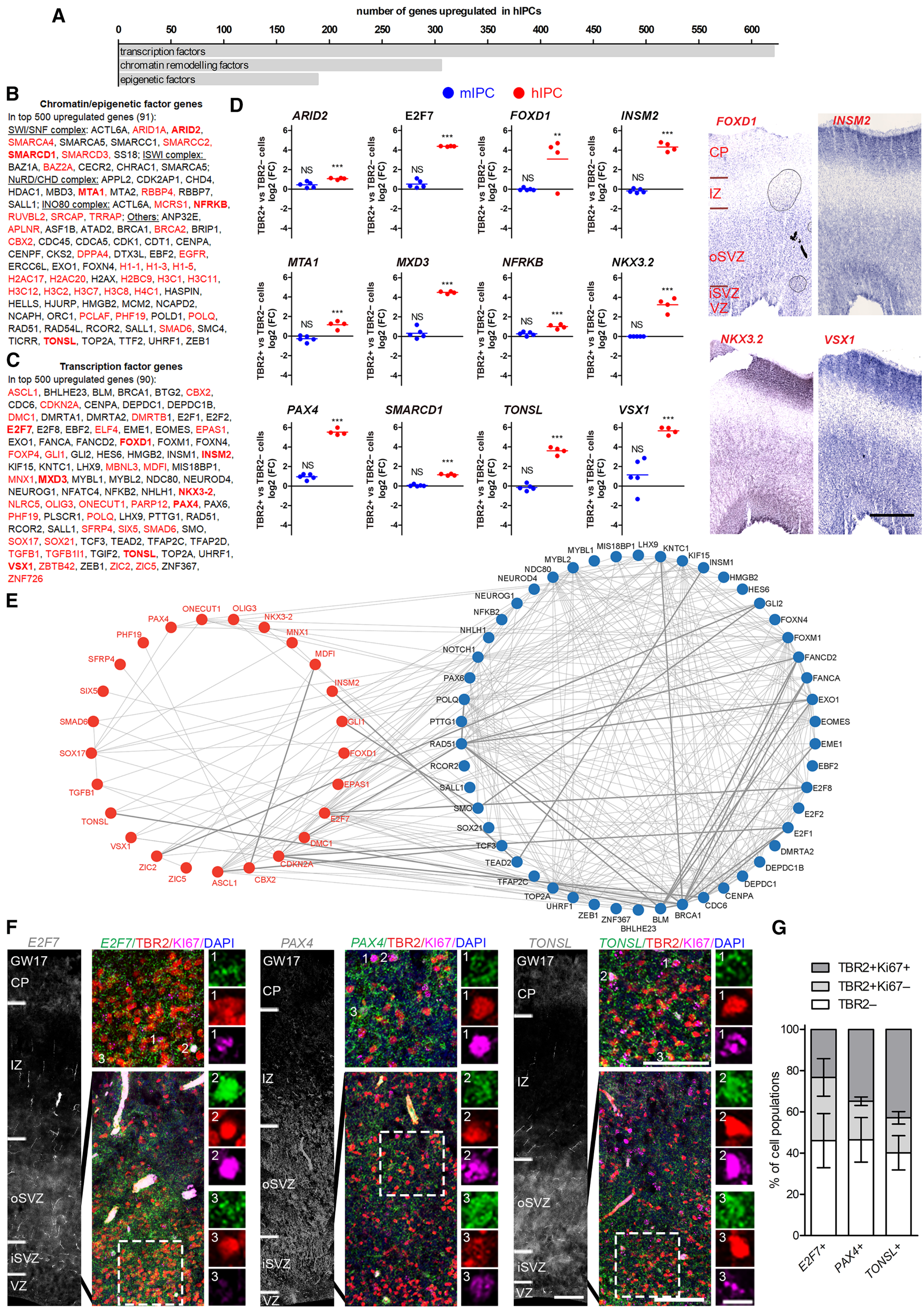

(legend on next page)

proliferation-related genes in hIPCs versus mIPCs, while genes linked to differentiation and axon/dendrite-formation were downregulated (Figure S2).

In summary, these findings suggest that mIPCs at E16.5 are primarily geared toward neuronal differentiation and migration, as evidenced by their specific gene expression profile. In contrast, hIPCs at GW14-17 exhibit a distinct expression pattern enriched in genes promoting cell cycle progression and proliferation, underscoring their role in generating a larger pool of progenitors before undergoing terminal differentiation into mature neurons.

Identification of hIPC transcriptional regulators

Investigating the transcriptional landscape of hIPCs, we found the upregulation of several genes involved in transcriptional regulation, chromatin remodeling, and epigenetic processes (Figures 3A–3D). Specifically, our analysis revealed a notable upregulation of chromatin factor genes in sorted hIPCs, with 307 genes showing significant upregulation (padj <0.01), including 91 among the top 500 upregulated genes (Figures 3B and 3D). These chromatin modifiers belong to several complexes such as SWI/SNF, ISWI, NuRD/CHD, and INO80 complex, indicating active chromatin remodeling in hIPCs (Figures 3B and 3D; Table S3). Notably, many of these upregulated chromatin-modifying genes were unique to hIPCs and not upregulated in mIPCs (Figures 3B and 3D, marked in red).

Additionally, we identified a robust upregulation of genes encoding histone proteins (*H2AC17, H2AC20, H2AX, H2BC9, H3C1, H3C2, H3C7, H3C8, H3C11, H3C12, H4C1*) or linker histones (*H1-1, H1-3, H1-5*) in hIPCs (Figure 3B). These histone components are integral to nucleosomes, which play a critical role in regulating DNA accessibility to transcriptional machinery. The elevated expression of histone genes in hIPCs suggests a tightly controlled gene regulatory environment distinct from TBR2− cells. Notably, this specific upregulation of histone-related genes was not observed in mIPCs (Figure 3B).

Furthermore, our transcriptomic analysis identified 618 upregulated TF genes in hIPCs, including prominent families such as C2H2-type zinc finger factors, homeodomain factors, and basic-helix-loop-helix (bHLH) factors (Table S3). Among these, 90 TFs ranked among the top 500 upregulated genes (Figures 3C and 3D), featuring known regulators of neurogenesis such as *EOMES* (*TBR2*), *NEUROD4*, and *NEUROG1*. Interestingly, several TFs previously unreported in brain development, such as *CDKN2A, DEPDC1, E2F7*, and *PAX4*, were also highly upregulated in hIPCs, suggesting novel roles in human cortical development.

To explore potential interactions among these upregulated TFs, we constructed a protein-protein interaction network using the STRING database (Figure 3E). This network analysis identified a highly interconnected network of the 90 top upregulated TFs, with 55 TFs common to both mouse and hIPCs (blue nodes) and 36 TFs specific to hIPCs (red nodes). Notably, certain TFs exhibited extensive interactions with others, positioning them centrally within the network, including TFs found in both species (*BLM, BRCA1, RAD51*) and those unique to hIPCs (*ASCL1, CDKN2A, E2F7*).

Among the TFs upregulated in hIPCs relative to TBR2− cells, the proliferation-related TFs E2F7, PAX4, and TONSL were particularly notable, as their expression has not previously been described in human cortical development. Their expression in IPCs was assessed by fluorescence *in situ* hybridization (FISH) for *E2F7, PAX4*, and *TONSL* in the GW17 human cortex sections, combined with immunostaining for TBR2 and the proliferation marker Ki67 (Figure 3F). All three transcripts were

Figure 3. Identification of novel hIPC-specific transcription regulators

(A) Graphical representation of Gene Ontology analysis of genes upregulated in TBR2+ IPCs vs. TBR2− cells isolated from the developing human cortex (p value <0.01, log2FC > 1, $N = 4$ RNA-seq libraries per condition), focusing on the regulation of gene expression.

(B and C) List of chromatin/epigenetic factor genes (B) and transcription factor genes (C) identified in the top 500 of upregulated genes of hIPCs sorted by their involvement in different complexes, with genes not upregulated in mIPCs highlighted red.

(D) Expression levels of selected genes (highlighted in bold in the gene list) as identified in the individual samples in RNA-seq of mIPCs and hIPCs ($N = 5$ RNA-seq libraries per condition for mIPCs and $N = 4$ RNA-seq libraries per condition for hIPCs) with micrographs showing *in situ* hybridization with distinctive expression in the GW20 human cortex.

(E) Protein-protein interaction network of transcription factors (TFs) found in the top 500 hIPC-upregulated genes. The list of TFs was imported into the STRING database (http://string-db.org/), and interactions between the differentially expressed transcription factors were extracted using default settings. Blue nodes represent TFs upregulated in both mIPCs and hIPCs, while red nodes represent TFs exclusively upregulated in hIPCs. The thin lines indicate a low interaction score (<0.4), while the thick lines indicate a medium or high interaction score (>0.4).

(F) Fluorescence *in situ* hybridizations probed for *E2F7, PAX4,* or *TONSL* combined with Ki67-specific antibody in the human cortex at GW17.

(G) Quantitative analysis of reciprocal TBR2, Ki67 co-expression with *E2F7, PAX4,* or *TONSL* in the GW17 human cortex ($N = 4$ images from a single human fetal cortical specimen). VZ, ventricular zone; iSVZ, inner subventricular zone; oSVZ, outer subventricular zone; IZ, intermediate zone; CP, cortical plate. $^{*}p < 0.05$, $^{**}p < 0.01$, and $^{***}p < 0.001$; NS, not significant. Scale bars, 2 mm (D), 500 μm (F, overview), 50 μm (F, higher magnifications), and 10 μm (F, single-cell panel).

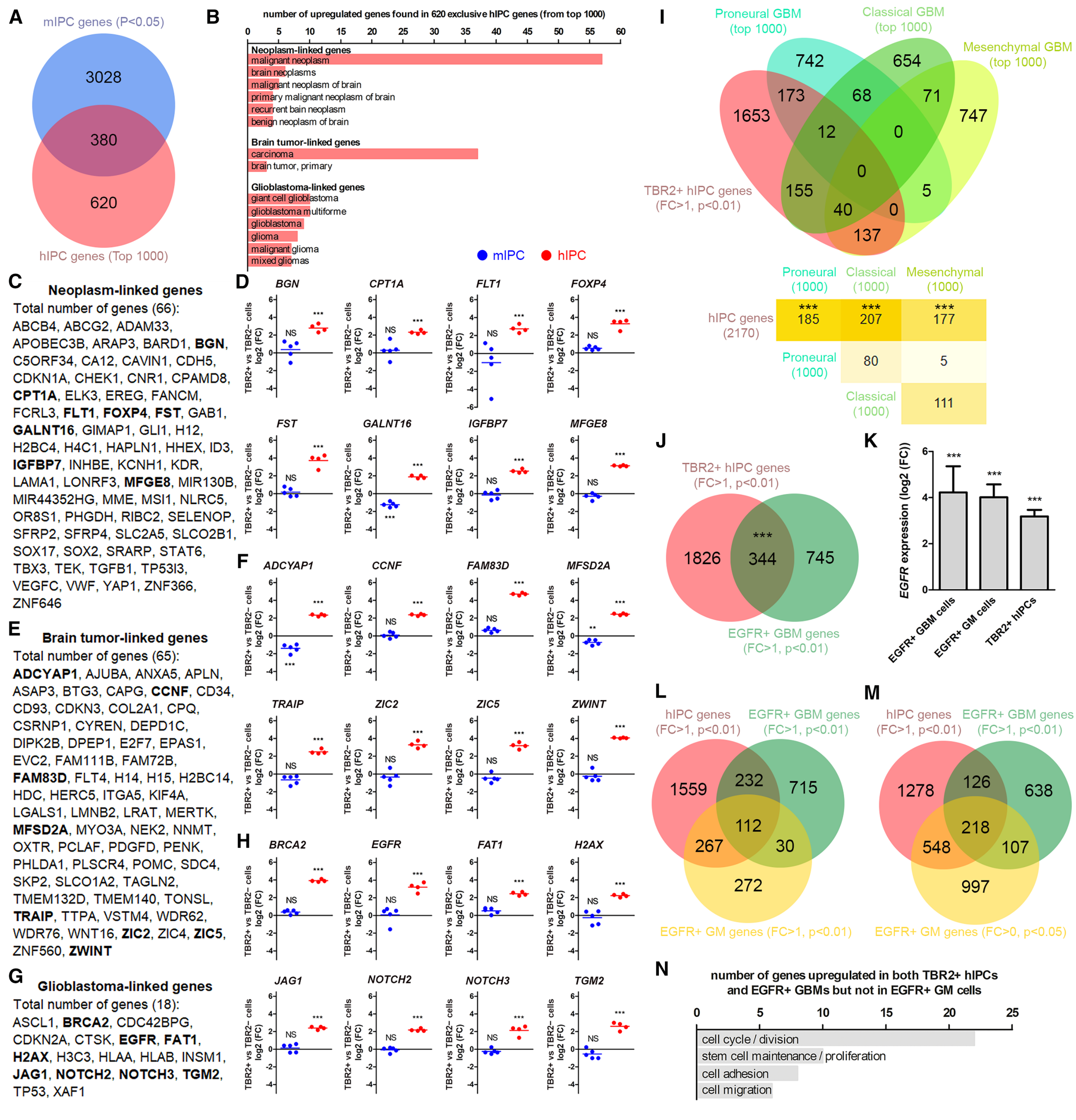

Figure 4. Mutations of hIPC-upregulated genes are linked to brain cancer

(A) Overlap of the top 1000 hIPC-upregulated genes with the 3408 genes upregulated in mIPCs (p value <0.05), identifying 620 hIPC-specific genes.

(B) Graphical representation of Human Phenotype Ontology for 620 hIPC-specific genes showing terms related to brain cancer.

(C–H) List (C, E, G) and selected genes (D, F, H) of hIPC-specific genes, which link to neoplasm (C, D), brain tumor (E, F), and glioblastoma (G, H). The expression level of selected genes is highlighted in bold in the adjoining gene list.

(I) Overlap of genes upregulated in hIPCs (p value <0.01, log2FC > 1) and top 1000 upregulated genes in GBM subtypes (Brennan et al., 2013).

(J) Overlap of genes upregulated in hIPCs and in EGFR+ GBM cells (p value <0.01, log2FC > 1).

(K) Expression levels of *EGFR* transcripts in EGFR+ GBM cells, EGFR+ GM cells, and TBR2+ hIPCs.

(legend continued on next page)

detected predominantly within the SVZ compartments and frequently co-localized with TBR2+ IPCs (Figure 3F). Quantitative analysis showed that the majority of *E2F7*+, *PAX4*+, or *TONSL*+ cells were TBR2+ IPCs (54.0 ± 13.2%, 53.6 ± 10.8%, and 59.9 ± 8.3%, respectively), indicating a preferential enrichment with IPCs. Remarkably, TBR2+ IPCs expressing *E2F7*, *PAX4*, or *TONSL* exhibited high proliferation rates, with 43.7 ± 4.5%, 63.6 ± 9.3%, and 71.6 ± 3.0% of cells being Ki67+, respectively (Figure 3H). These results demonstrate that the expression of *E2F7, PAX4*, and *TONSL* is preferentially associated with proliferative IPC subsets.

E2F7, a transcriptional repressor, regulates genes involved in cell cycle progression, specifically during the G1/S transition (Di Stefano et al., 2003; Li et al., 2008). Previous studies have highlighted its role in promoting proliferation in glioblastoma (Yang et al., 2020; Yu et al., 2020a), and our findings suggest it may similarly enhance proliferation in hIPCs. PAX4, traditionally known for its involvement in pancreatic development, has been shown to promote β-cell proliferation, potentially via the activation of the c-myc-Id2 pathway (Brun et al., 2004; Hu He et al., 2011; Lorenzo et al., 2017). In insulinomas, elevated *PAX4* expression was detected (Miyamoto et al., 2001). Although a role for PAX4 in brain development has not been previously established, its notable upregulation in hIPCs indicates that it may have a previously unrecognized function in promoting the proliferation of IPCs. TONSL, essential for homologous recombination (HR) repair during DNA replication, stabilizes replication forks and resolves DNA damage, ensuring proper cell cycle progression (Huang et al., 2022; O'Donnell et al., 2010). Neural progenitors undergo rapid cell division, making them highly reliant on robust DNA repair mechanisms to prevent genomic instability. In cancer biology, TONSL is critical for the survival and proliferation of cancer stem cells, particularly in glioblastoma (Lee et al., 2023). This suggests it may play a role in maintaining proliferative capacity in other stem cell populations, such as IPCs. Together, the upregulation of these proliferation-related TFs in hIPCs suggests mechanistic insights into the high proliferative capacity of these cells. However, the precise functions of E2F7, PAX4, and TONSL in regulating IPC proliferation remain to be fully elucidated and warrant further investigation.

Overall, our findings underscore distinctive transcriptional and epigenetic features of hIPCs compared to their mouse counterparts, highlighting a unique regulatory landscape crucial for human cortical development.

Comparative analysis of conserved and human-specific IPC genes in cortical malformations and brain tumors

In our previous study, we found several cortical malformation phenotypes associated with mutations of mIPC-upregulated genes (Ulmke et al., 2021). We hypothesized that key transcriptome elements in the mIPCs are conserved in hIPCs and play critical roles in cortical development. To explore this hypothesis, we performed a systematic human phenotype ontology (HPO) analysis (Robinson et al., 2008) to investigate the involvement of 413 genes with conserved expression in mIPCs and hIPCs (p value <0.01, log2FC > 1, Figure 2A) in diseases. Indeed, mutation in several of these evolutionary conserved IPC genes are associated with cortical malformations such as primary microcephaly (*BUB1B, CDK6, CENPF, CENPJ, KIF14, NCAPH, NTNG2, ORC1, SLC1A2, STIL*, Figure S3A), indicating conserved functions in cortical development among species.

We further examined phenotypes linked to hIPC-upregulated genes by comparing the top 1000 most upregulated genes in hIPCs to the 3408 genes significantly upregulated in mIPCs (p value <0.05) (Figure 4A). We applied this threshold ($p < 0.05$) to ensure the inclusion of all potentially upregulated genes in mIPCs, preventing false classification of genes as human-specific due to minor differences in statistical significance. Of the top 1000 hIPC-upregulated genes, 620 were not upregulated in mIPCs. Thus, their upregulation is considered as human-specific (Figures 4A; Table S4). Among these human-specifically upregulated genes, several were associated with cortical malformations such as microcephaly (*CDK5RAP2, CEP152, CIT, H4C3, KNL1, MFSD2A, NUP214, POC1A, TBCD, TRAIP, TUBG1, TUBGCP6, WDR62, ZNF292*, Figure S3B). Additionally, we identified several hIPC-upregulated genes (p value <0.01) that are not upregulated in mIPCs and have been linked to broader categories such as global developmental delay (*AP1B1, CNOT1, COL4A1, DIAPH1, FCSK, GPAA1, GTF3C3, INTS1, KDM1A, KDM3B, KDM5C, LNPK, MAPK8IP3, OXR1, POLR2A, SLC5A6, SMARCC2, SMARCD1, SPTBN2, SUZ12, SZT2, TMEM94, TKFC, TRAF7, TRRAP, ZMIZ1, ZNF142, ZNF335*, Figure S3C) and nervous system disorders (*ABCG2, ASCL1, ID3, PENK, SELENOP, TTPA*, Figure S3D).

Remarkably, a substantial subset of the top 620 hIPC-upregulated genes is associated with proliferation-related

(L and M) Overlap of genes upregulated in hIPCs, in EGFR+ GBM cells, and in EGFR+ GM cells (p value <0.01, log2FC > 1 (L) or p value <0.05, log2FC > 0 (M), N = 3 independent human samples per group).

(N) Graphical representation of Gene Ontology for 126 genes upregulated in both TBR2+ hIPCs and EGFR+ GBM cells, but not in EGFR+ GM cells. N = 5 RNA-seq libraries per condition for mIPCs and N = 4 RNA-seq libraries per condition for hIPCs. *$p < 0.05$, **$p < 0.01$, and ***$p < 0.001$; NS, not significant.

brain diseases (Figure 4B; Table S4). Many hIPC-upregulated genes are linked to neoplasms (Figures 4C and 4D) and brain tumors (Figures 4E and 4F). Among these, glioma represents the most prevalent category of primary brain cancer (Miller et al., 2021). For instance, *CDKN3*, which is highly expressed in TBR2+ hIPCs (Figure 2G), is also elevated in low-grade glioma (LGG), where increased *CDKN3* expression correlates with poor patient survival (Cheng et al., 2025). CDKN3 directly regulates the cell cycle and promotes LGG proliferation, while its silencing has been shown to inhibit glioma cell growth (Cheng et al., 2025). Glioblastoma multiforme (GBM), the most aggressive and lethal form of glioma, is highlighted by its particularly poor prognosis (Delgado-Martin and Medina, 2020; Gimple et al., 2019). Our analysis identified 18 genes associated with GBM that are exclusively upregulated in hIPCs (*ASCL1, BRCA2, CDC42BPG, CDKN2A, CTSK,* epidermal growth factor receptor *(EGFR), FAT1, H2AX, H3C3, HLAA, HLAB, INSM1, JAG1, NOTCH2, NOTCH3, TGM2, TP53, XAF1*, Figures 4G and 4H).

GBMs can be classified into three transcriptional subtypes—proneural, classical, and mesenchymal—according to differences in gene expression profiles (Wang et al., 2017b). By comparing genes upregulated in hIPCs to the top 1000 upregulated genes in each GBM subtype from a screening of over 500 GBM tumors (Brennan et al., 2013), we observed gene overlaps with all three subtypes, with the greatest overlap in the classical subtype (Figure 4I). These overlaps were all higher than a maximum random overlap determined by random sampling and cumulative distribution function (CDF) calculation, and thus highly significant (Table S4). To further investigate the link of hIPC-upregulated genes and GBM, we analyzed an RNA-seq dataset comparing EGFR+ and EGFR− GBM cells (Tome-Garcia et al., 2017). EGFR is the predominant marker of the classical GBM subtype (Verhaak et al., 2010) and is known for its role in neural stem cell pool maintenance (Romano and Bucci, 2020). Our analysis revealed a substantial overlap, with 344 genes upregulated in both TBR2+ hIPCs and EGFR+ GBMs (p value <0.01, log2FC > 1) (Figure 4J). The overlap with EGFR+ GBMs is thus higher (32% of EGFR+ GBM upregulated genes) than with the GBM subtypes (18–21%) (Figures 4I and 4J). Again, random sampling and CDF calculation confirmed the significance of the overlap of genes upregulated in TBR2+ hIPCs and EGFR+ GBMs (Table S4).

Tome-Garcia et al. compared the EGFR+ GBMs to EGFR+ stem cell populations from the human germinal matrix (GM) at GW19-22, identifying significant transcriptomic similarities between GM and GBM EGFR+ populations, particularly in genes related to cell cycle and proliferation pathways (Tome-Garcia et al., 2017). Since *EGFR* is also highly expressed in TBR2+ hIPCs (Figure 4K), the hIPC data from our study expand the gene set common to both developing and neoplastic EGFR+ populations. The stem cell-like characteristics shared between GM and GBM may reflect broader progenitor cell properties, as many of these genes are also upregulated in TBR2+ IPCs (Figure 4L). Moreover, GBM may exhibit IPC-specific features not present in EGFR+ progenitors of the GM. We identified 126 genes that are upregulated in both TBR2+ hIPCs and EGFR+ GBMs (p value <0.01, log2FC > 1) but not in EGFR+ GM cells (p value <0.05, log2FC > 0 to include also slightly upregulated genes) (Figure 4M). These genes are primarily involved in cell cycle regulation and proliferation (Figure 4N). Furthermore, overlapping genes are associated with cell adhesion and migration (e.g., *ADGRG1, ASAP3, CDH24, CDHR1, CTNNAL1, EFNB1, ITGB8, LAMA1, PLCG1, PTK7*), indicating that the overlap extends beyond mere proliferation-related genes.

In summary, our studies revealed that conserved genes between mIPCs and hIPCs are associated with cortical malformations, indicating evolutionarily conserved functions in cortical development. Additionally, human-specific IPC genes are linked to both cortical malformations and brain tumors, including GBM, suggesting distinct proliferative and differentiative properties that contribute to the complex structure of the human brain and its associated diseases. Certain proliferative and cell cycle-related mechanisms, but also mechanisms affecting cell adhesion and migration, in GBM may be specifically related to IPCs, highlighting potential IPC-specific roles in GBM tumorigenesis.

Identification of human IPC-upregulated genes

To systematically identify genes with a specific function in hIPCs, we compared the top 500 hIPC-upregulated genes with the 3408 genes significantly upregulated in mIPCs (p value <0.05) (Ulmke et al., 2021) (Figure 5A). 234 of the top 500 hIPC-upregulated genes were also upregulated in mIPCs, indicating their evolutionary conservation. Conversely, 266 of these top hIPC-upregulated genes did not show enriched expression in mIPCs and are thus considered hIPC-specific upregulated genes (Figures 5A and 5C; Table S5). Of these 266 hIPC-specific upregulated genes, only seven (*EGFR, ASCL1, KIF4A, KNL1, LGALS1, TMEM123, TMEM158*, Figure 5B) had been previously identified in scRNA-seq analyses of the human developing cortex (Pebworth et al., 2021; Polioudakis et al., 2019).

The 266 top hIPC-upregulated genes can be categorized into different gene families encoding TFs, chromatin remodelers, lncRNAs, signaling molecules, cell cycle regulators, chromosome segregation proteins, post-translational modification (PTM) enzymes, cell-cell interaction molecules, and factors linked to DNA repair and apoptosis (Figures 5D–5K). Notably, several hIPC-upregulated genes

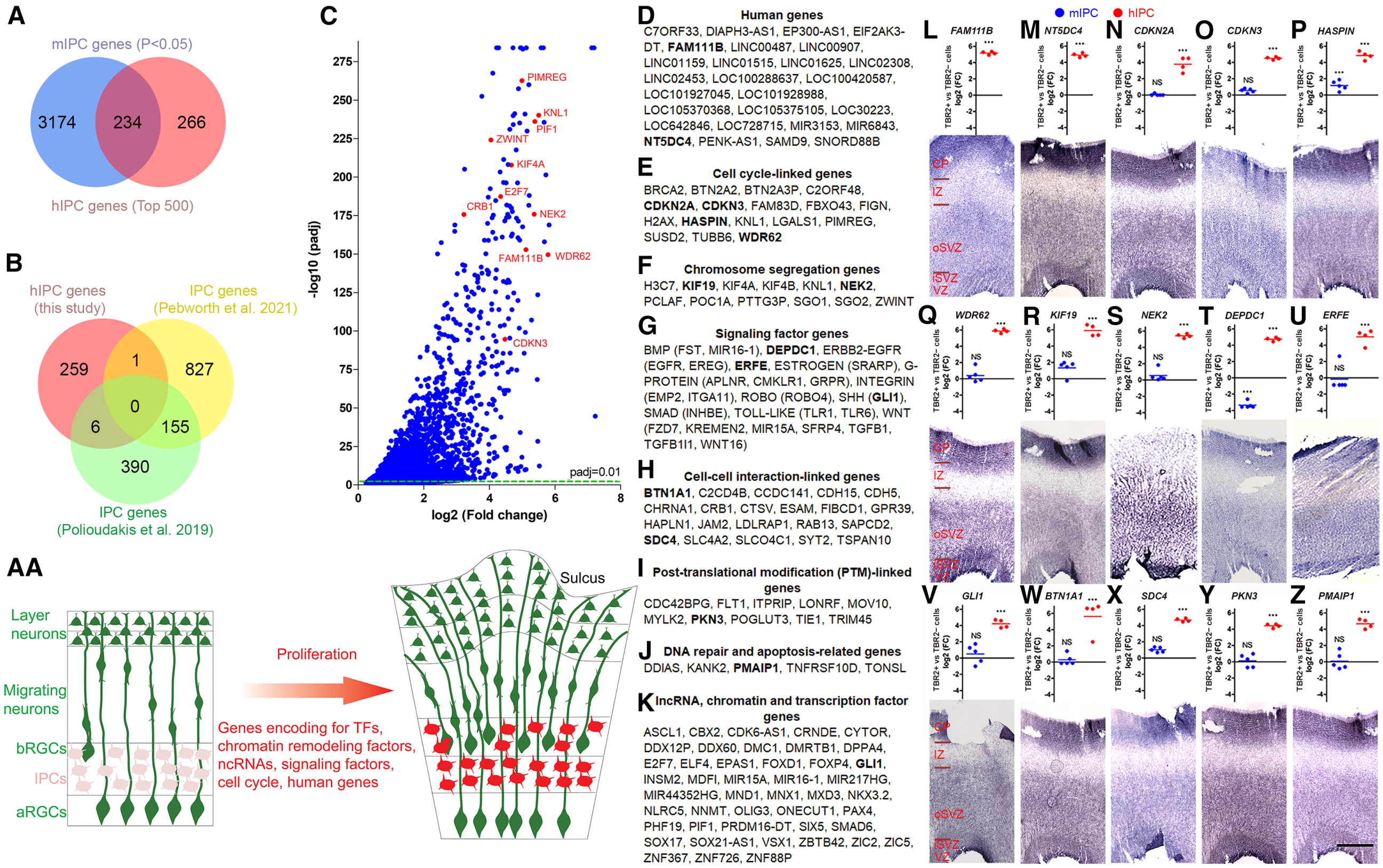

Figure 5. Identification and validation of novel human IPC-specific genes

(A) Overlap of the top 500 hIPC-upregulated genes with the 3408 genes upregulated in mIPCs (p value <0.05), identifying 266 hIPC-specific genes.

(B) Overlap of 266 hIPC-specific genes with IPC genes identified in previous studies (Pebworth et al., 2021; Polioudakis et al., 2019).

(C) Volcano plot shows the enriched expression of hIPC-upregulated genes (in red).

(D–K) List of hIPC-specific genes that functionally fall under the categories: human genes (D), cell cycle (E), chromosome segregation (F), signaling factor (G), cell-cell interaction (H), post-translational modification (PTM, I), DNA repair and apoptosis (J), lncRNA, chromatin and transcription factors (K).

(L–Z) RNA-seq with sorted mIPC and hIPC samples (*upper*) and ISH analysis with human cortical tissue at GW20 (*lower*) for the expression of selected genes, which are highlighted in bold in the adjoining gene list in (D-K). (AA) Hypothetical schematic showing that genes encoding transcription factors (TFs), chromatin remodeling factors, ncRNAs, signaling factors, and cell cycle proteins, in combination with other human-specific genes, drive the proliferation of human IPCs, ultimately resulting in an increased cortical size. VZ, ventricular zone; iSVZ, inner subventricular zone; oSVZ, outer subventricular zone; IZ, intermediate zone; CP, cortical plate; aRGCs, apical radial glial cells; IPCs, intermediate progenitor cells; bRGCs, basal radial glial cells. N = 5 RNA-seq libraries per condition for mIPCs and N = 4 RNA-seq libraries per condition for hIPCs. *p < 0.05, **p < 0.01, and ***p < 0.001; NS, not significant. Scale bars, 2 mm.

are known to be human-specific genes (Figures 5D, 5L, and 5M).

ISH analysis confirmed the subventricular expression of 15 of the hIPC-specific upregulated genes (Figures 5L–5Z). In contrast, no specific expression of these genes was observed in the SVZ of mice (Figures 5L–5Z, GenePaint.org) (Ulmke et al., 2021).

In summary, we identified and validated several hIPC-specific upregulated genes encoding for different protein families. These genes likely contribute to the unique characteristics of hIPCs in the developing human cortex, potentially resulting in a greater number of neurons and a larger cortex (Figures 5AA).

IPC-specific ectopic expression of *hCDKN3* in developing mouse cortex promotes IPC proliferation and cortical neurogenesis

To elucidate the role of genes strongly upregulated in hIPCs during cortical development, we investigated the function of CDKN3 in IPCs. Among the genes preferentially expressed in hIPCs, *CDKN3* ranks in the top 100 most upregulated genes (Figures 1F and 2G; Table S1). GO analysis

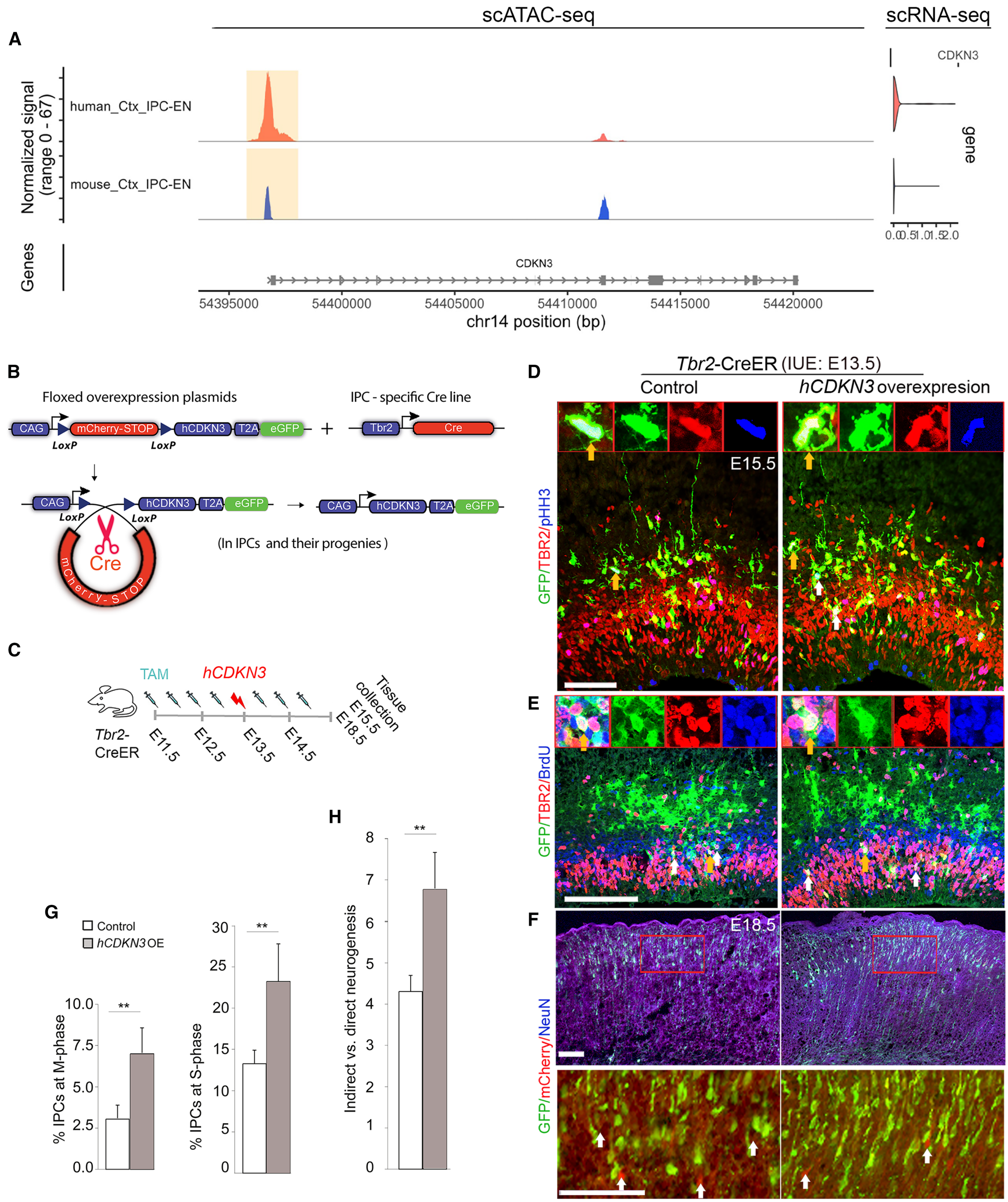

Figure 6. *CDKN3* expression regulates IPC proliferation and neurogenesis

(A) Cross-species single-cell ATAC-seq analysis showing chromatin accessibility at the *CDKN3* locus in mouse and human IPCs together with gene expression levels (original datasets from Liu et al., 2025; Wang et al., 2025).

(legend continued on next page)

showed that *CDKN3* belongs to the top gene category of cell cycle regulation (Table S5). Additionally, CDKN3 is well-documented for its role in promoting proliferation in cancer cells (Li et al., 2017, 2022; Liu et al., 2019; Wang et al., 2017a; Yu et al., 2017, 2020b; Zhang et al., 2015). *Cdkn3* is not upregulated in mIPCs compared with TBR2− cells (Table S1 of Ulmke et al., 2021), and no visible expression of *Cdkn3* is detected in the mouse VZ/SVZ (Figure S1D). Consistently, *Cdkn3* was not identified among the genes enriched in apical or basal progenitors relative to other telencephalic cell types in an E12.5 mouse single-cell RNA-seq dataset (Table S7 and Table S12 of Moreau et al., 2021).

To investigate potential regulatory mechanisms underlying the species-specific expression of *CDKN3*, chromatin accessibility at the *CDKN3* locus was analyzed using publicly available single-cell ATAC-seq datasets from developing mouse cortex (Liu et al., 2025) and human cortex (Wang et al., 2025) (Figures S4A–S4C). As a control, chromatin accessibility was examined at the housekeeping gene *ACTB* and at the canonical progenitor marker *VIM*, which previously showed conserved accessibility patterns between mouse and human neural progenitors (Liu et al., 2025) (Figure S4D). Consistent with these findings, comparable accessibility profiles were observed at the *ACTB* and *VIM* loci across both species. Cross-species integration further revealed accessible chromatin at the *CDKN3* locus in IPCs of both species, indicating that the locus is permissive for transcription (Figure 6A). However, chromatin accessibility was markedly higher in hIPCs than in mIPCs, consistent with the substantially higher *CDKN3* expression observed in hIPCs. These findings suggest that species-specific differences in chromatin accessibility contribute to the elevated expression of *CDKN3* in hIPCs.

To investigate whether *CDKN3* upregulation affects IPC proliferation, we utilized a Cre/*loxP* plasmid system containing a loxP-flanked mCherry-STOP sequence followed by an *hCDKN3*-IRES-eGFP cassette (Figure 6B) (Sokpor et al., 2024). The control group consisted of embryos electroporated with an empty mCherry-eGFP-only plasmid. For targeted *CDKN3* overexpression (OE) in IPCs, the constructs were introduced into tamoxifen (TAM)-treated *Tbr2/Eomes*-CreER mouse embryos (Pimeisl et al., 2013) via *in utero* electroporation (IUE) at E13.5 (Figure 6C). Under the control of IPC-specific *TBR2* regulatory elements, Cre recombinase specifically switches expression from mCherry to *hCDKN3* and eGFP in IPCs and their progeny, in both control and *hCDKN3*OE (Figure 6B).

At E15.5, we observed a higher ratio of TBR2+/eGFP+ cells in the SVZ to mCherry+/TBR2− cells in the VZ in cortices electroporated with the *hCDKN3* construct compared to controls, suggesting an expansion of the TBR2+ IPC population upon *hCDKN3* OE (Figure S4E). S-phase progenitors were pulse-labeled via injection of the thymidine analog BrdU 1 h prior to tissue isolation. We analyzed the proliferation rates of TBR2+ IPCs by comparing the number of TBR2+/pHH3+ (mitotic phase) and TBR2+/BrdU+ (S-phase) cells to all eGFP+ IPCs in control and OE cortices. *CDKN3* upregulation significantly elevated the proportion of basal mitosis (eGFP+/TBR2+/pHH3+ or BrdU+) in the targeted IPC population (Figures 6D, 6E, and 6G).

In cortical development, RGCs contribute to neurogenesis both directly, by generating neurons themselves, and indirectly, by producing IPCs that in turn give rise to neurons. It should be noted that this analysis was designed and executed specifically to serve as a control for the effects of IPC-specific *hCDKN3* OE, not to test the impact of *hCDKN3* on direct neurogenesis. In fact, because the *Tbr2/Eomes*-CreER system limits *hCDKN3* expression to the IPC lineage, no impact on RGCs was expected. To exclude the effect of IPC-specific *hCDKN3* OE on direct neurogenesis, we repeated IUE at E13.5. As RGCs undergo a single division within 24 h at midgestation, we isolated the tissue at E14.5 and applied double immunostaining for mCherry along with neuronal markers TUBB3 and Hu proteins HuC/HuD to quantify neurons produced directly from mCherry+ RGCs. In the control, mCherry+

(B) Sketch of the plasmid used to introduce IPC-specific *hCDKN3* overexpression (OE) in tamoxifen (TAM)-treated *Tbr2*/*Eomes*-CreER mouse embryos.

(C) Injection scheme of TAM in pregnant *Tbr2*-CreER mice in combination with *in utero* electroporation (IUE) to deliver the *hCDKN3* OE plasmid.

(D, E, and F) Micrographs at low and high magnification showing eGFP, TBR2 and pHH3 (D), eGFP, TBR2 and BrdU (E) or eGFP, mCherry and NeuN (F) immunostaining in the E15.5 (D, E) or E18.5 (F) *Tbr2*-CreER mouse cortex electroporated with an empty mCherry-eGFP-only plasmid as control or *hCDKN3* overexpression plasmid at E13.5 to overexpress *CDKN3* in the transfected cells. Upper images in D and E represent magnifications of individual cells marked by an orange arrow. Lower images in F represent magnifications of areas marked by a red frame.

(G) Quantification of the proportion of TBR2+/pHH3+ (M-phase) and TBR2+/BrdU+ (S-phase) cells among eGFP+ IPCs in E15.5 control and *hCDKN3*OE cortices ($N = 6$ images analyzed from 3 independent embryos per condition).

(H) Quantification of the ratio of eGFP+ neurons to mCherry+ neurons as a measure of indirect neurogenesis in E18.5 control and *hCDKN3*OE cortices ($N = 6$ images from 3 independent embryos per condition). *$p < 0.05$, **$p < 0.01$, and ***$p < 0.001$. Scale bars, 100 μm.

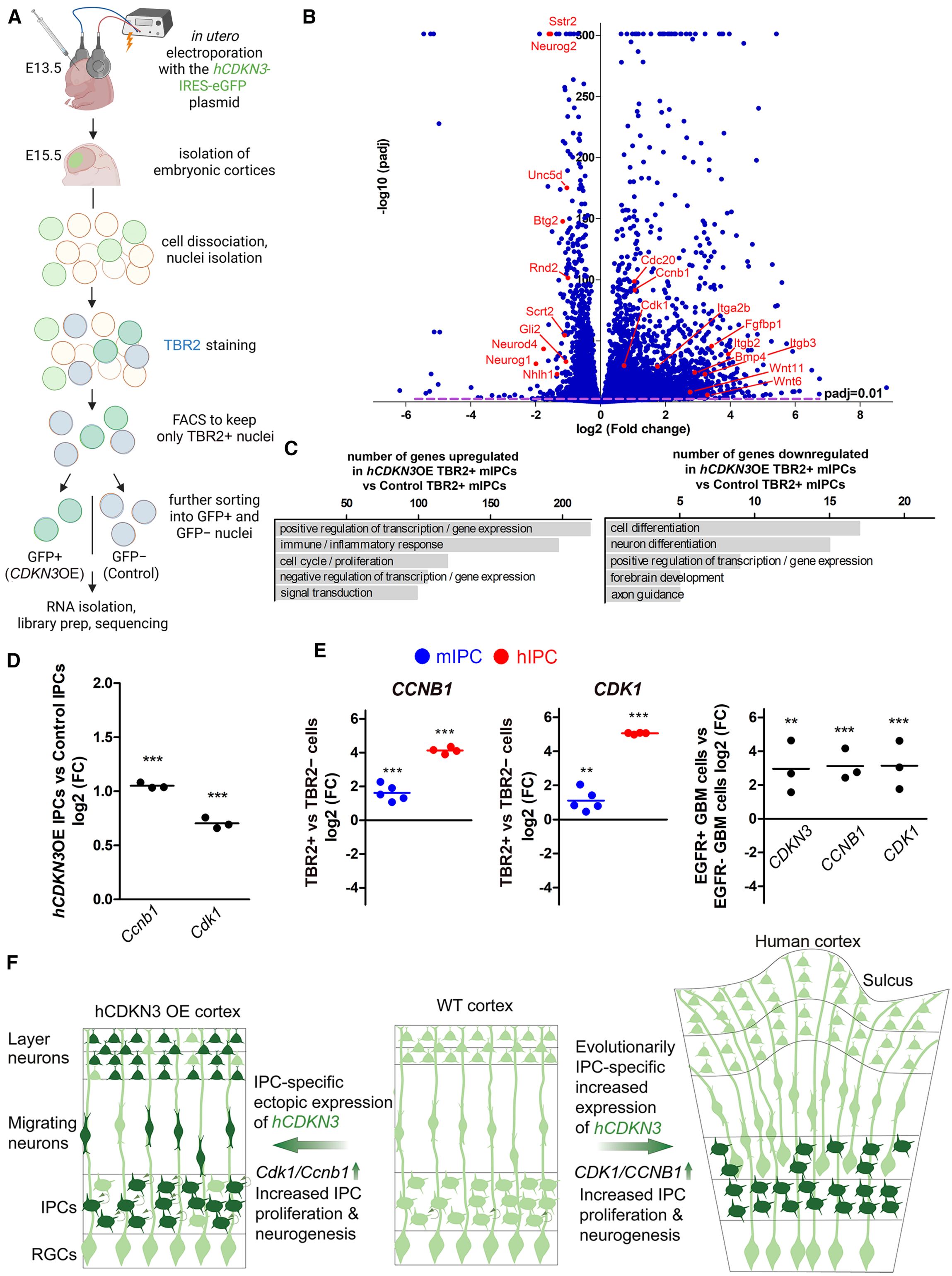

Figure 7. *CDKN3* activates proliferative transcriptional programs in IPCs

(A) Schematic of the experimental workflow combining *in utero* electroporation and FACS to isolate *hCDKN3* overexpressing (OE) TBR2+ cells and control TBR2+ cells for RNA sequencing. (Created with BioRender) (B) Volcano plot shows differential gene expression between

(legend continued on next page)

electroporated cells were situated in the SVZ/IZ, with a subset co-expressing TUBB3 and HuC/HuD (19% ± 3.5% of all mCherry+ cells), indicating differentiated neurons originating directly from RGCs (Figures S4F–S4I). Relative to controls, the cortex electroporated with the *hCDKN3* plasmid showed a comparable percentage of TUBB3 and HuC/HuD-positive neurons among the mCherry+ cells (Figures S4F–S4I), confirming that IPC-specific *hCDKN3* OE in our experimental paradigm does not influence direct neurogenesis.

To assess if the IPC-specific *hCDKN3* OE facilitates indirect neurogenesis, we counted eGFP+/NeuN+ neurons in *hCDKN3* plasmid-electroporated and control cortices at E18.5 (Figures 6F and 6H). Given the lack of difference in direct neurogenesis between *hCDKN3*-electroporated and control cortices, mCherry+/NeuN+ neurons were utilized as internal controls. Our analysis revealed a higher ratio of eGFP+ neurons to mCherry+ neurons in *hCDKN3*-electroporated cortices compared to controls.

As apoptosis in the VZ/SVZ and cortical layers, as well as direct neurogenesis, showed no significant differences between *hCDKN3* gain-of-function and control mice, our data suggest that IPC-specific OE of *hCDKN3* facilitates cortical neurogenesis by enhancing IPC proliferation.

CDKN3 plays a crucial role in cell cycle regulation, particularly in facilitating the transition from the G1 to S phase (Liu et al., 2019; Nalepa et al., 2013). Mechanistically, *CDKN3* OE has been linked to the upregulation of the cell cycle regulator *CDK1*. CDK1 interacts with Cyclin B1 (CCNB1) to form the CCNB1–CDK1 complex, which phosphorylates target substrates, promoting cell cycle progression (Hayward et al., 2019; Wang et al., 2017c).

To elucidate how CDKN3 regulates IPC proliferation, we performed bulk RNA sequencing of GFP+ (*CDKN3*OE) and GFP− (Control) TBR2+ IPCs isolated from E15.5 mouse cortices following IUE with the *hCDKN3* OE plasmid at E13.5 (Figure 7A; Figures S5A–S5D; Table S6).

Differential expression analysis revealed a pronounced transcriptional shift upon *CDKN3* OE. Canonical intermediate progenitor differentiation genes were strongly downregulated (*Btg2, Neurog1, Neurog2, Neurod1, Neurod4, Myt1, Nhlh1, Sstr2, Scrt2, Unc5d, Rnd2;* Figure 7B). Conversely, genes associated to mitotic expansion were upregulated, including core G2/M cell-cycle regulators (*Cdc20, Ccnb1*), signaling factors such as integrin signaling (*Itgb3, Itga1, Itga2b, Itgb2, Itgb5, Fyb1, Fcer1g, Itgam, Syk, Plek, Adam33, Thy1, Itgal, Tspan32, Vav1, Fgr, Vtn, Col3a1, Tyrobp, Tec, Ptk2b, Madcam1*), Tgf signaling (*Tgfb1, Tgfbr2, Tgfbr3, Acvrl1, Nrros, Spi1, Mstn, Wfikkn2, Arap1, Lrrc32, Npnt, Cldn5, Cdh5, Dab2, Col3a1, Col1a2, Ccl2, Lpxn, Lgals9, Eng*), Fgf signaling (*Fgfbp1, Fgf3, Fgf9, Fgf18, Ret, Ntrk1, Pdgfra, Kl, Csf1r, Flt1, Epha8, Flt4, Tie1, Insrr, Pdgfb, Kdr, Tek, Epha2*), Wnt signaling (*Wnt9a, Wnt6, Wnt11, Wnt4, Foxd1, Fzd6, Isl1, Ednra, Adgra2, Cdh1, Col6a1, Rspo2, Gpc3, Sostdc1*), BMP signaling (*Bmp7, Bmp4, Bmp6, Bmp5, Acvrl1, Egr1, Msx2, Tmprss6, Hfe, Gdf5, Vsir, Tmem119, Sostdc1, Msx1*). Consistent with this shift toward a proliferative transcriptional state, GO analysis identified enrichment of pathways related to cell-cycle progression and proliferation among genes upregulated in *hCDKN3*OE IPCs, whereas genes associated with cellular and neuronal differentiation were preferentially downregulated (Figure 7C; Table S6).

Notably, *CDKN3* OE induced significant upregulation of *Cdk1* and *Ccnb1* (Figure 7D). Several proto-oncogenes and other genes involved in cancer pathways were also upregulated (Figures S5E and S5F), suggesting CDKN3 drives a gene expression profile reminiscent of proliferative systems observed in cancers.

Consistent with this finding, *CDK1* and *CCNB1* are strongly upregulated in hIPCs under physiological conditions (Figure 7E). Although these factors are also upregulated in mIPCs, their expression levels are comparatively lower, suggesting that hIPCs exhibit a more robust activation of this proliferative regulatory network, which may represent an evolutionary adaptation contributing to their increased proliferative capacity.

Moreover, EGFR+ glioblastoma cells, which exhibit aggressive proliferation, also show strong upregulation of *CDK1* and *CCNB1* (Figure 7E), further underscoring a mechanistic parallel between developmental IPC proliferation and tumorigenic cell-cycle activation. Together, these

*hCDKN3*OE mIPCs and control mIPCs. Genes associated with cell-cycle/proliferation (upregulated) and neuronal differentiation (downregulated) are highlighted in red (N = 4 RNA-seq libraries per condition).

(C) Gene Ontology analyses of genes up- (top) or downregulated (bottom) in *hCDKN3*OE TBR2+ mIPCs compared to control TBR2+ mIPCs (p value <0.01, log2FC > 1 or < −1).

(D) Expression levels of *Ccnb1* and *Cdk1* in individual RNA-seq libraries of *hCDKN3*OE versus control mIPCs.

(E) *CCNB1* and *CDK1* expression levels identified in RNA-seq of mIPCs and hIPCs (TBR2+ IPCs versus TBR2− non-IPCs, N = 5 RNA-seq libraries per condition for mIPCs and N = 4 RNA-seq libraries per condition for hIPCs) and *CDKN3, CCNB1,* and *CDK1* expression levels in EGFR+ GBM cells (N = 3 independent human samples per group).

(F) Model illustrates how IPC-specific elevated expression of *hCDKN3* – either through experimental overexpression or during cortical evolution – enhances IPC proliferation and indirect neurogenesis. RGCs, radial glial cells; IPCs, intermediate progenitors. *p < 0.05, **p < 0.01, and ***p < 0.001.

data suggest that while CDKN3 retains a conserved role as a G1/S-phase regulator, its heightened expression in hIPCs may promote the species-specific expansion of neuronal output (Figure 7F).

DISCUSSION

In summary, numerous identified genes code for elements of the transcriptional, chromatin, and signaling systems in both mIPCs and hIPCs. These gene sets play established or hypothesized regulatory roles in cell division, proliferation, differentiation, and survival. Key elements of the IPC transcriptome are conserved across species from mice to humans, playing critical roles in cortical development. Mutations in these elements are likely implicated in cortical malformations and dysfunction. Notably, specific characteristics of hIPCs enable them to exhibit greater proliferation and delayed differentiation compared to their murine counterparts, which might be the prerequisite for the development of a larger cortex. Remarkably, numerous hIPC-upregulated genes are associated with aberrant proliferation-related brain diseases, including neoplasms and glioblastoma. Additionally, hIPCs may produce various groups of neurons, including INs, whereas mIPCs predominantly generate ENs.

Further research into the regulation of IPC proliferation and differentiation during cortical development will not only address fundamental biological questions but also shed light on the processes underlying related diseases.

Identification of CDKN3 as a key regulator in hIPC proliferation

In this study, we identified CDKN3 as a novel regulator of hIPC proliferation. *CDKN3* ranks among the top 100 most upregulated genes in hIPCs, with its enriched expression being specific to humans and not observed in mice.

Although CDKN3 has been traditionally classified as a negative regulator of the cell cycle due to its inhibition of CDK1, growing evidence supports an alternative role in promoting cell cycle progression in pathological contexts. An oncogenic role for aberrant OE of *CDKN3* has been implicated in multiple cancer types, in which CDKN3 was found to promote cell cycle progression, primarily by facilitating the transition from the G1 to S phase. For example, CDKN3 promotes proliferation in bladder cancer (Li et al., 2022) and gastric cancer, where its knockdown leads to reduced proliferation and invasion (Li et al., 2017). In esophageal squamous cell carcinoma, CDKN3 directly accelerates the G1/S transition, thereby driving continuous cell division and tumor growth (Liu et al., 2019). Mechanistically, CDKN3 has been shown to upregulate *CDK1* expression in renal cell carcinoma (Cen et al., 2021), a key regulator of the G1/S checkpoint and DNA replication.

To directly assess whether CDKN3 exerts a similar proliferative effect in IPCs, we used IUE to express human *CDKN3* in mIPCs under control of the *Tbr2* promoter. *CDKN3* OE significantly increased mitosis in targeted IPCs, leading to an expansion of the IPC pool. This increased progenitor pool was associated with elevated indirect neurogenesis, demonstrating that CDKN3 promotes neurogenesis by enhancing IPC proliferation. Consistent with these cellular effects, RNA-seq analysis of *CDKN3*-overexpressing mIPCs revealed the upregulation of the cell cycle regulators *Ccnb1* and *Cdk1*. Together, these findings establish CDKN3 as a key driver of IPC-mediated cortical expansion through direct regulation of cell cycle control.

Notably, the CDKN3–CCNB1–CDK1 regulatory axis observed in our *CDKN3*-OE experiments parallels mechanisms reported in cancers. EGFR+ GBMs, which exhibit high proliferative capacity, also show strong upregulation of *CDKN3*, *CDK1*, and *CCNB1*, mirroring the transcriptional response seen in *CDKN3*OE IPCs. This overlap highlights a mechanistic parallel between developmental IPC proliferation and tumorigenic cell-cycle activation, suggesting that proliferative programs active during human cortical development may be co-opted in tumorigenesis.

Comparing the genes upregulated upon *hCDKN3* OE in mIPCs with those upregulated in hIPCs and EGFR+ GBM cells, we identified 14 genes that were commonly upregulated in all three conditions: *ABHD3, ACSS3, AEBP1, ANXA5, CCNB1, CDC20, CDHR1, DSG2, FAM83D, GPR88, LRRC17, OXTR, PDGFD*, and *TMEM158* (Figure S5G). Next to CCNB1, also CDC20 and FAM83D are important regulators of cell cycle progression and proliferation but have not previously been associated with CDKN3 activity. Their co-regulation in the context of both development and cancer suggests that CDKN3 may control a broader proliferative gene network than previously appreciated.

Taken together, our results establish CDKN3 as a key regulator of hIPC proliferation and neurogenesis, linking its function to both cortical expansion and brain tumor susceptibility. Future studies will be necessary to elucidate whether CDKN3 interacts with additional species-specific regulatory mechanisms to sustain the elevated proliferative capacity of hIPCs.

Mutation of hIPC-upregulated genes links to brain tumors

Investigating the phenotypes associated with mutations of genes upregulated in hIPCs, we identified a significant number of genes linked to cancer (Figure 4). This association was not observed in murine IPC genes, suggesting that the high upregulation of cancer-associated genes

may be a human-specific characteristic of hIPCs. A typical feature of many cancer cells is elevated proliferation. Given that a substantial fraction of IPCs, particularly in the human brain, are expected to exhibit high proliferation rates, many cancer associations can be attributed to this characteristic. However, the large number of hIPC-upregulated genes implicated in brain cancers suggests a potential specific role of hIPCs in tumorigenesis.

Contrary to earlier beliefs that gliomas arise from mature glial cells at their observed locations, current understanding suggests that the tumors arise from neurogenic niches within the adult SVZ and subsequently spread to other brain regions (Jiang and Uhrbom, 2012; Lee et al., 2018; Sanai et al., 2005). This theory is substantiated by genomic data obtained from patient samples (Lee et al., 2018) and the observed high frequency of gliomas near neurogenic niches (Bhaduri et al., 2020; Mandal et al., 2020). Although previous studies have linked NSCs to the generation of GBMs (Ignatova et al., 2002; Singh et al., 2004), adult NSCs of the SVZ exhibit few prototumorigenic characteristics (Walton et al., 2009). However, when NSCs are induced to differentiate, they generate IPCs, which temporarily exhibit several glioma-like features, including aneuploidy, disrupted growth-contact inhibition, cell cycle changes, and a lack of response to growth factors (Walton et al., 2009). Hence, IPCs have been proposed as the source of "stem cell-derived" tumors (Walton et al., 2009). High-grade glioma cells expressing IPC genes exhibit a high Ki-67 labeling index, revealing high proliferation rates and suggesting a pluripotent NSC lineage nature (Bielle et al., 2017; Yoshida et al., 2023). Notably, several genes upregulated exclusively in hIPCs, were also implicated in GBM (Figures 4G and 4H). The strong upregulation of many cancer-associated genes in hIPCs in the developing cortex found in this study supports the hypothesis that IPCs could be the origin of certain tumors in the adult brain. Furthermore, our findings revealed that 344 genes (32%) upregulated in EGFR+ GBM tumors were also upregulated in hIPCs, underscoring the potential of IPCs as cells of origin for EGFR-positive GBMs.

In a subgroup of oligodendrogliomas, OE of IPC genes has been observed alongside morphological and IHC similarities to the embryonic SVZ (Henderson-Smith et al., 2016). In these tumors, a proliferating tumor cell subpopulation with IPC characteristics was identified (Henderson-Smith et al., 2016). The exhibition of IPC features is potentially critical in the oncogenesis of these oligodendroglioma, as it is linked to increased tumor necrosis and elevated recurrence rates (Henderson-Smith et al., 2016).

IPCs have further been implicated as potential cells of origin in TLX-expressing GBM tumors (Hakes and Brand, 2020). The upregulation of the neural stem cell regulator TLX in GBM correlates with poor diagnosis (Park et al., 2010). TLX initiates tumorigenesis by reverting IPCs to a stem cell-like state through the repression of ASCL1, a direct target of TLX (Hakes and Brand, 2020). Re-expression of ASCL1 successfully halted tumor formation, highlighting a potential therapeutic strategy of counteracting high expression of IPC genes to suppress tumors originating from IPCs (Hakes and Brand, 2020).

The identification of IPC-specific upregulated markers is thus essential not only for understanding brain development but also for identifying tumor cell subpopulations. Discovering pathways that regulate IPC proliferation could guide the development of therapeutic targets for tumors originating from these cells.

RESOURCE AVAILABILITY

Lead contact

Requests for further information and resources should be directed to and will be fulfilled by the lead contact, Tran Tuoc (tran.tuoc@ruhr-uni-bochum.de).

Materials availability

Plasmids generated in this study are available from the lead contact on request.

Data and code availability

- RNA-seq data have been deposited in the NCBI Sequence Read Archive (SRA) under the BioProject accession number PRJNA1213291.
- This paper does not report original code.
- Any additional information required to reanalyze the data reported in this paper is available from the lead contact upon request.

ACKNOWLEDGMENTS

We acknowledge ZVM team for their expert animal care and M. Peters and P. Bonowitz for FACS. We thank S. Arnold for providing *Tbr2*-CreER mouse line. This work was supported by TU432/3-1, TU432/6-1 DFG grants (TT), F1008N-20, IF-027-22 RUB/FoRUM grants (TT), GRK2862/1/492434978 (HPN), NextGenerationEU via Bulgarian National Recovery and Resilience Plan, Project #BG-RRP-2.004-0009-C03 (ABT, MNI).

AUTHOR CONTRIBUTIONS

P.A.U., H.D.N., M.P., and P.B. performed TBR2+ cell sorting. P.A.U., H.D.N., X.M., and K.L. performed RNA-seq and analyzed RNA-seq data. T.K., E.K., and A.B.T. provided human tissue for FACS. P.A.U., H.D.N., A.M., L.P., and T.T. performed IUE and histological analysis. M.N.I., L.V.V., A.B.T., A.A., and G.C. performed IHC and ISH of the human cortex. H.P.N. provided research tools and offered discussions for the study. T.T. conceived the study. T.T. and P.A.U. wrote the manuscript.

DECLARATION OF INTERESTS

The authors declare no competing interests.

DECLARATION OF GENERATIVE AI AND AI-ASSISTED TECHNOLOGIES IN THE WRITING PROCESS

During the preparation of this work, the authors used ChatGPT and Perplexity to improve the writing style. After using this tool, the authors reviewed and edited the content as needed and take full responsibility for the content of the publication.

STAR★METHODS

Detailed methods are provided in the online version of this paper and include the following:

- KEY RESOURCES TABLE
- EXPERIMENTAL MODEL AND STUDY PARTICIPANT DETAILS
  - Human fetal brain
  - Transgenic mice
- METHOD DETAILS
  - TBR2+ nuclei sorting from embryonic human cortex
  - TBR2+/GFP+ cell sorting from mouse cortex
  - RNA-sequencing and bioinformatics analysis
  - *In utero* electroporation (IUE)
  - Immunohistochemistry (IHC)
  - Ratio of S-phase determination
  - Human fetal brain processing for ISH
  - *In situ* hybridization validation (ISH)
  - scRNA-seq dataset and deconvolution
  - Cross-species scRNA-seq and scATAC-seq analysis
  - Glioblastoma datasets
- QUANTIFICATION AND STATISTICAL ANALYSIS
  - General quantification and statistical analysis
  - Statistical analysis of gene set overlap

SUPPLEMENTAL INFORMATION

Supplemental information can be found online at https://doi.org/10.1016/j.stemcr.2026.103012.

Received: January 21, 2025
Revised: June 11, 2026
Accepted: June 11, 2026
Published: July 9, 2026

REFERENCES

Albert, M., Kalebic, N., Florio, M., Lakshmanaperumal, N., Haffner, C., Brandl, H., Henry, I., and Huttner, W.B. (2017). Epigenome profiling and editing of neocortical progenitor cells during development. Embo J. *36*, 2642–2658. https://doi.org/10.15252/embj.201796764.

Amamoto, R., Zuccaro, E., Curry, N.C., Khurana, S., Chen, H.H., Cepko, C.L., and Arlotta, P. (2020). FIN-Seq: transcriptional profiling of specific cell types from frozen archived tissue of the human central nervous system. Nucleic Acids Res. *48*, e4. https://doi.org/10.1093/nar/gkz968.

Andrews, M.G., and Pearson, C.A. (2024). Toward an understanding of glucose metabolism in radial glial biology and brain development. Life Sci. Alliance *7*, e202302193. https://doi.org/10.26508/lsa.202302193.

Arlotta, P., Molyneaux, B.J., Chen, J., Inoue, J., Kominami, R., and Macklis, J.D. (2005). Neuronal subtype-specific genes that control corticospinal motor neuron development in vivo. Neuron *45*, 207–221.

Bhaduri, A., Di Lullo, E., Jung, D., Müller, S., Crouch, E.E., Espinosa, C.S., Ozawa, T., Alvarado, B., Spatazza, J., Cadwell, C.R., et al. (2020). Outer Radial Glia-like Cancer Stem Cells Contribute to Heterogeneity of Glioblastoma. Cell Stem Cell *26*, 48–63.e6. https://doi.org/10.1016/j.stem.2019.11.015.

Bielle, F., Ducray, F., Mokhtari, K., Dehais, C., Adle-Biassette, H., Carpentier, C., Chanut, A., Polivka, M., Poggioli, S., Rosenberg, S., et al. (2017). Tumor cells with neuronal intermediate progenitor features define a subgroup of 1p/19q co-deleted anaplastic gliomas. Brain Pathol. *27*, 567–579. https://doi.org/10.1111/bpa.12434.

Brennan, C.W., Verhaak, R.G.W., McKenna, A., Campos, B., Noushmehr, H., Salama, S.R., Zheng, S., Chakravarty, D., Sanborn, J.Z., Berman, S.H., et al. (2013). The somatic genomic landscape of glioblastoma. Cell *155*, 462–477. https://doi.org/10.1016/j.cell.2013.09.034.

Brun, T., Franklin, I., St-Onge, L., Biason-Lauber, A., Schoenle, E.J., Wollheim, C.B., and Gauthier, B.R. (2004). The diabetes-linked transcription factor PAX4 promotes beta-cell proliferation and survival in rat and human islets. J. Cell Biol. *167*, 1123–1135. https://doi.org/10.1083/jcb.200405148.

Cen, J., Liang, Y., Huang, Y., Pan, Y., Shu, G., Zheng, Z., Liao, X., Zhou, M., Chen, D., Fang, Y., et al. (2021). Circular RNA circSDHC serves as a sponge for miR-127-3p to promote the proliferation and metastasis of renal cell carcinoma via the CDKN3/E2F1 axis. Mol. Cancer *20*, 19. https://doi.org/10.1186/s12943-021-01314-w.

Charvet, C.J. (2020). Closing the gap from transcription to the structural connectome enhances the study of connections in the human brain. Dev. Dyn. *249*, 1047–1061. https://doi.org/10.1002/dvdy.218.

Cheng, H., Meng, X., Zhang, Y., Wang, P., and Lu, Y. (2025). Identification of CDKN3 overexpression as a marker of poor prognosis and potential therapeutic target in low-grade glioma. Sci. Rep. *15*, 2679. https://doi.org/10.1038/s41598-025-86338-8.

Dehay, C., Kennedy, H., and Kosik, K.S. (2015). The outer subventricular zone and primate-specific cortical complexification. Neuron *85*, 683–694. https://doi.org/10.1016/j.neuron.2014.12.060.

Delgado-Martín, B., and Medina, M.Á. (2020). Advances in the Knowledge of the Molecular Biology of Glioblastoma and Its Impact in Patient Diagnosis, Stratification, and Treatment. Adv. Sci. *7*, 1902971. https://doi.org/10.1002/advs.201902971.

Dessau, R.B., and Pipper, C.B. (2008). ["R"–project for statistical computing]. Ugeskr Laeger *170*, 328–330.

Di Stefano, L., Jensen, M.R., and Helin, K. (2003). E2F7, a novel E2F featuring DP-independent repression of a subset of E2F-regulated genes. Embo J *22*, 6289–6298. https://doi.org/10.1093/emboj/cdg613.

Durinck, S., Spellman, P.T., Birney, E., and Huber, W. (2009). Mapping identifiers for the integration of genomic datasets with the

R/Bioconductor package biomaRt. Nat. Protoc. *4*, 1184–1191. https://doi.org/10.1038/nprot.2009.97.

Fan, X., Dong, J., Zhong, S., Wei, Y., Wu, Q., Yan, L., Yong, J., Sun, L., Wang, X., Zhao, Y., et al. (2018). Spatial transcriptomic survey of human embryonic cerebral cortex by single-cell RNA-seq analysis. Cell Res. *28*, 730–745. https://doi.org/10.1038/s41422-018-0053-3.

Fietz, S.A., Kelava, I., Vogt, J., Wilsch-Bräuninger, M., Stenzel, D., Fish, J.L., Corbeil, D., Riehn, A., Distler, W., Nitsch, R., and Huttner, W.B. (2010). OSVZ progenitors of human and ferret neocortex are epithelial-like and expand by integrin signaling. Nat. Neurosci. *13*, 690–699. https://doi.org/10.1038/nn.2553.

Galaxy Community (2024). The Galaxy platform for accessible, reproducible, and collaborative data analyses: 2024 update. Nucleic Acids Res. *52*, W83–W94. https://doi.org/10.1093/nar/gkae410.

Gimple, R.C., Bhargava, S., Dixit, D., and Rich, J.N. (2019). Glioblastoma stem cells: lessons from the tumor hierarchy in a lethal cancer. Genes Dev. *33*, 591–609. https://doi.org/10.1101/gad.324301.119.

Hakes, A.E., and Brand, A.H. (2020). Tailless/TLX reverts intermediate neural progenitors to stem cells driving tumourigenesis via repression of asense/ASCL1. eLife *9*, e53377. https://doi.org/10.7554/eLife.53377.

Hansen, D.V., Lui, J.H., Parker, P.R.L., and Kriegstein, A.R. (2010). Neurogenic radial glia in the outer subventricular zone of human neocortex. Nature *464*, 554–561. https://doi.org/10.1038/nature08845.

Haubensak, W., Attardo, A., Denk, W., and Huttner, W.B. (2004). Neurons arise in the basal neuroepithelium of the early mammalian telencephalon: a major site of neurogenesis. Proc. Natl. Acad. Sci. USA *101*, 3196–3201. https://doi.org/10.1073/pnas.0308600100.

Hayward, D., Alfonso-Pérez, T., Cundell, M.J., Hopkins, M., Holder, J., Bancroft, J., Hutter, L.H., Novak, B., Barr, F.A., and Gruneberg, U. (2019). CDK1-CCNB1 creates a spindle checkpoint-permissive state by enabling MPS1 kinetochore localization. J. Cell Biol. *218*, 1182–1199. https://doi.org/10.1083/jcb.201808014.

Henderson-Smith, A., Corneveaux, J.J., De Both, M., Cuyugan, L., Liang, W.S., Huentelman, M., Adler, C., Driver-Dunckley, E., Beach, T.G., and Dunckley, T.L. (2016). Next-generation profiling to identify the molecular etiology of Parkinson dementia. Neurol. Genet. *2*, e75. https://doi.org/10.1212/NXG.0000000000000075.

Hevner, R.F. (2019). Intermediate progenitors and Tbr2 in cortical development. J. Anat. *235*, 616–625. https://doi.org/10.1111/joa.12939.

Hu He, K.H., Lorenzo, P.I., Brun, T., Jimenez Moreno, C.M., Aeberhard, D., Vallejo Ortega, J., Cornu, M., Thorel, F., Gjinovci, A., Thorens, B., et al. (2011). In vivo conditional Pax4 overexpression in mature islet beta-cells prevents stress-induced hyperglycemia in mice. Diabetes *60*, 1705–1715. https://doi.org/10.2337/db10-1102.

Huang, D.W., Sherman, B.T., and Lempicki, R.A. (2009). Systematic and integrative analysis of large gene lists using DAVID bioinformatics resources. Nat. Protoc. *4*, 44–57. https://doi.org/10.1038/nprot.2008.211.

Huang, Y.C., Yuan, W., and Jacob, Y. (2022). The Role of the TSK/TONSL-H3.1 Pathway in Maintaining Genome Stability in Multicellular Eukaryotes. Int. J. Mol. Sci. *23*, 9029. https://doi.org/10.3390/ijms23169029.

Ignatova, T.N., Kukekov, V.G., Laywell, E.D., Suslov, O.N., Vrionis, F.D., and Steindler, D.A. (2002). Human cortical glial tumors contain neural stem-like cells expressing astroglial and neuronal markers in vitro. Glia *39*, 193–206. https://doi.org/10.1002/glia.10094.

Jiang, Y., and Uhrbom, L. (2012). On the origin of glioma. Upsala J. Med. Sci. *117*, 113–121. https://doi.org/10.3109/03009734.2012.658976.

Kawaguchi, A., Ikawa, T., Kasukawa, T., Ueda, H.R., Kurimoto, K., Saitou, M., and Matsuzaki, F. (2008). Single-cell gene profiling defines differential progenitor subclasses in mammalian neurogenesis. Development *135*, 3113–3124. https://doi.org/10.1242/dev.022616.

Kerimoglu, C., Pham, L., Tonchev, A.B., Sakib, M.S., Xie, Y., Sokpor, G., Ulmke, P.A., Kaurani, L., Abbas, E., Nguyen, H., et al. (2021). H3 acetylation selectively promotes basal progenitor proliferation and neocortex expansion. Sci. Adv. *7*, eabc6792. https://doi.org/10.1126/sciadv.abc6792.

Kim, D., Langmead, B., and Salzberg, S.L. (2015). HISAT: a fast spliced aligner with low memory requirements. Nat. Methods *12*, 357–360. https://doi.org/10.1038/nmeth.3317.

Korsunsky, I., Millard, N., Fan, J., Slowikowski, K., Zhang, F., Wei, K., Baglaenko, Y., Brenner, M., Loh, P.R., and Raychaudhuri, S. (2019). Fast, sensitive and accurate integration of single-cell data with Harmony. Nat. Methods *16*, 1289–1296. https://doi.org/10.1038/s41592-019-0619-0.

Kowalczyk, T., Pontious, A., Englund, C., Daza, R.A.M., Bedogni, F., Hodge, R., Attardo, A., Bell, C., Huttner, W.B., and Hevner, R.F. (2009). Intermediate neuronal progenitors (basal progenitors) produce pyramidal-projection neurons for all layers of cerebral cortex. Cereb. Cortex *19*, 2439–2450.

Lawrence, M., Gentleman, R., and Carey, V. (2009). rtracklayer: an R package for interfacing with genome browsers. Bioinformatics *25*, 1841–1842. https://doi.org/10.1093/bioinformatics/btp328.

Lee, H., Ha, S., Choi, S., Do, S., Yoon, S., Kim, Y.K., and Kim, W.Y. (2023). Oncogenic Impact of TONSL, a Homologous Recombination Repair Protein at the Replication Fork. Int. J. Mol. Sci. *24*, 9530. https://doi.org/10.3390/ijms24119530.

Lee, J.H., Lee, J.E., Kahng, J.Y., Kim, S.H., Park, J.S., Yoon, S.J., Um, J.Y., Kim, W.K., Lee, J.K., Park, J., et al. (2018). Human glioblastoma arises from subventricular zone cells with low-level driver mutations. Nature *560*, 243–247. https://doi.org/10.1038/s41586-018-0389-3.

Li, J., Ran, C., Li, E., Gordon, F., Comstock, G., Siddiqui, H., Cleghorn, W., Chen, H.Z., Kornacker, K., Liu, C.G., et al. (2008). Synergistic function of E2F7 and E2F8 is essential for cell survival and embryonic development. Dev. Cell *14*, 62–75. https://doi.org/10.1016/j.devcel.2007.10.017.

Li, M., Che, N., Jin, Y., Li, J., and Yang, W. (2022). CDKN3 Overcomes Bladder Cancer Cisplatin Resistance via LDHA-Dependent Glycolysis Reprogramming. OncoTargets Ther. *15*, 299–311. https://doi.org/10.2147/OTT.S358008.

Li, M., Santpere, G., Imamura Kawasawa, Y., Evgrafov, O.V., Gulden, F.O., Pochareddy, S., Sunkin, S.M., Li, Z., Shin, Y., Zhu, Y., et al. (2018). Integrative functional genomic analysis of human brain development and neuropsychiatric risks. Science *362*, eaat7615. https://doi.org/10.1126/science.aat7615.

Li, Y., Ji, S., Fu, L.Y., Jiang, T., Wu, D., and Meng, F.D. (2017). Knockdown of Cyclin-Dependent Kinase Inhibitor 3 Inhibits Proliferation and Invasion in Human Gastric Cancer Cells. Oncol. Res. *25*, 721–731. https://doi.org/10.3727/096504016X14772375848616.

Li, Z., Tyler, W.A., Zeldich, E., Santpere Baró, G., Okamoto, M., Gao, T., Li, M., Sestan, N., and Haydar, T.F. (2020). Transcriptional priming as a conserved mechanism of lineage diversification in the developing mouse and human neocortex. Sci. Adv. *6*, eabd2068. https://doi.org/10.1126/sciadv.abd2068.

Liao, Y., Smyth, G.K., and Shi, W. (2014). featureCounts: an efficient general purpose program for assigning sequence reads to genomic features. Bioinformatics *30*, 923–930. https://doi.org/10.1093/bioinformatics/btt656.

Liu, J., Min, L., Zhu, S., Guo, Q., Li, H., Zhang, Z., Zhao, Y., Xu, C., and Zhang, S. (2019). Cyclin-Dependent Kinase Inhibitor 3 Promoted Cell Proliferation by Driving Cell Cycle from G1 to S Phase in Esophageal Squamous Cell Carcinoma. J. Cancer *10*, 1915–1922. https://doi.org/10.7150/jca.27053.

Liu, Y., Luo, X., Sun, Y., Chen, K., Hu, T., You, B., Xu, J., Zhang, F., Cheng, Q., Meng, X., et al. (2025). Comparative single-cell multiome identifies evolutionary changes in neural progenitor cells during primate brain development. Dev. Cell *60*, 414–428.e8. https://doi.org/10.1016/j.devcel.2024.10.005.

Loo, L., Simon, J.M., Xing, L., McCoy, E.S., Niehaus, J.K., Guo, J., Anton, E.S., and Zylka, M.J. (2019). Single-cell transcriptomic analysis of mouse neocortical development. Nat. Commun. *10*, 134. https://doi.org/10.1038/s41467-018-08079-9.

Lorenzo, P.I., Juárez-Vicente, F., Cobo-Vuilleumier, N., García-Domínguez, M., and Gauthier, B.R. (2017). The Diabetes-Linked Transcription Factor PAX4: From Gene to Functional Consequences. Genes *8*, 101. https://doi.org/10.3390/genes8030101.

Love, M.I., Huber, W., and Anders, S. (2014). Moderated estimation of fold change and dispersion for RNA-seq data with DESeq2. Genome Biol. *15*, 550. https://doi.org/10.1186/s13059-014-0550-8.

Lui, J.H., Hansen, D.V., and Kriegstein, A.R. (2011). Development and evolution of the human neocortex. Cell *146*, 18–36. https://doi.org/10.1016/j.cell.2011.06.030.

Mandal, A.S., Romero-Garcia, R., Hart, M.G., and Suckling, J. (2020). Genetic, cellular, and connectomic characterization of the brain regions commonly plagued by glioma. Brain *143*, 3294–3307. https://doi.org/10.1093/brain/awaa277.

Matho, K.S., Huilgol, D., Galbavy, W., He, M., Kim, G., An, X., Lu, J., Wu, P., Di Bella, D.J., Shetty, A.S., et al. (2021). Genetic dissection of the glutamatergic neuron system in cerebral cortex. Nature *598*, 182–187. https://doi.org/10.1038/s41586-021-03955-9.

Miller, K.D., Ostrom, Q.T., Kruchko, C., Patil, N., Tihan, T., Cioffi, G., Fuchs, H.E., Waite, K.A., Jemal, A., Siegel, R.L., and Barnholtz-Sloan, J.S. (2021). Brain and other central nervous system tumor statistics, 2021. CA Cancer J. Clin. *71*, 381–406. https://doi.org/10.3322/caac.21693.

Miyamoto, T., Kakizawa, T., Ichikawa, K., Nishio, S., Kajikawa, S., and Hashizume, K. (2001). Expression of dominant negative form of PAX4 in human insulinoma. Biochem. Biophys. Res. Commun. *282*, 34–40. https://doi.org/10.1006/bbrc.2001.4552.

Miyata, T., Kawaguchi, A., Saito, K., Kawano, M., Muto, T., and Ogawa, M. (2004). Asymmetric production of surface-dividing and non-surface-dividing cortical progenitor cells. Development *131*, 3133–3145.

Molyneaux, B.J., Goff, L.A., Brettler, A.C., Chen, H.H., Hrvatin, S., Rinn, J.L., and Arlotta, P. (2015). DeCoN: genome-wide analysis of in vivo transcriptional dynamics during pyramidal neuron fate selection in neocortex. Neuron *85*, 275–288. https://doi.org/10.1016/j.neuron.2014.12.024.

Moreau, M.X., Saillour, Y., Cwetsch, A.W., Pierani, A., and Causeret, F. (2021). Single-cell transcriptomics of the early developing mouse cerebral cortex disentangle the spatial and temporal components of neuronal fate acquisition. Development *148*, dev197962. https://doi.org/10.1242/dev.197962.

Nalepa, G., Barnholtz-Sloan, J., Enzor, R., Dey, D., He, Y., Gehlhausen, J.R., Lehmann, A.S., Park, S.J., Yang, Y., Yang, X., et al. (2013). The tumor suppressor CDKN3 controls mitosis. J. Cell Biol. *201*, 997–1012. https://doi.org/10.1083/jcb.201205125.

Newman, A.M., Liu, C.L., Green, M.R., Gentles, A.J., Feng, W., Xu, Y., Hoang, C.D., Diehn, M., and Alizadeh, A.A. (2015). Robust enumeration of cell subsets from tissue expression profiles. Nat. Methods *12*, 453–457. https://doi.org/10.1038/nmeth.3337.

Noctor, S.C., Martínez-Cerdeño, V., Ivic, L., and Kriegstein, A.R. (2004). Cortical neurons arise in symmetric and asymmetric division zones and migrate through specific phases. Nat. Neurosci. *7*, 136–144.

Nonaka-Kinoshita, M., Reillo, I., Artegiani, B., Martínez-Martínez, M.Á., Nelson, M., Borrell, V., and Calegari, F. (2013). Regulation of cerebral cortex size and folding by expansion of basal progenitors. Embo J *32*, 1817–1828. https://doi.org/10.1038/emboj.2013.96.

Nowakowski, T.J., Bhaduri, A., Pollen, A.A., Alvarado, B., Mostajo-Radji, M.A., Di Lullo, E., Haeussler, M., Sandoval-Espinosa, C., Liu, S.J., Velmeshev, D., et al. (2017). Spatiotemporal gene expression trajectories reveal developmental hierarchies of the human cortex. Science *358*, 1318–1323. https://doi.org/10.1126/science.aap8809.

O'Donnell, L., Panier, S., Wildenhain, J., Tkach, J.M., Al-Hakim, A., Landry, M.C., Escribano-Diaz, C., Szilard, R.K., Young, J.T.F., Munro, M., et al. (2010). The MMS22L-TONSL complex mediates recovery from replication stress and homologous recombination. Mol. Cell *40*, 619–631. https://doi.org/10.1016/j.molcel.2010.10.024.

Park, H.J., Kim, J.K., Jeon, H.M., Oh, S.Y., Kim, S.H., Nam, D.H., and Kim, H. (2010). The neural stem cell fate determinant TLX

promotes tumorigenesis and genesis of cells resembling glioma stem cells. Mol. Cells *30*, 403–408. https://doi.org/10.1007/s10059-010-0122-z.

Pebworth, M.P., Ross, J., Andrews, M., Bhaduri, A., and Kriegstein, A.R. (2021). Human intermediate progenitor diversity during cortical development. Proc. Natl. Acad. Sci. USA *118*, e2019415118. https://doi.org/10.1073/pnas.2019415118.

Pimeisl, I.M., Tanriver, Y., Daza, R.A., Vauti, F., Hevner, R.F., Arnold, H.H., and Arnold, S.J. (2013). Generation and characterization of a tamoxifen-inducible Eomes(CreER) mouse line. Genesis *51*, 725–733. https://doi.org/10.1002/dvg.22417.

Pinto, L., Mader, M.T., Irmler, M., Gentilini, M., Santoni, F., Drechsel, D., Blum, R., Stahl, R., Bulfone, A., Malatesta, P., et al. (2008). Prospective isolation of functionally distinct radial glial subtypes–lineage and transcriptome analysis. Mol. Cell. Neurosci. *38*, 15–42.

Polioudakis, D., de la Torre-Ubieta, L., Langerman, J., Elkins, A.G., Shi, X., Stein, J.L., Vuong, C.K., Nichterwitz, S., Gevorgian, M., Opland, C.K., et al. (2019). A Single-Cell Transcriptomic Atlas of Human Neocortical Development during Mid-gestation. Neuron *103*, 785. https://doi.org/10.1016/j.neuron.2019.06.011.

Pollen, A.A., Nowakowski, T.J., Chen, J., Retallack, H., Sandoval-Espinosa, C., Nicholas, C.R., Shuga, J., Liu, S.J., Oldham, M.C., Diaz, A., et al. (2015). Molecular identity of human outer radial glia during cortical development. Cell *163*, 55–67. https://doi.org/10.1016/j.cell.2015.09.004.

Qian, X., Shen, Q., Goderie, S.K., He, W., Capela, A., Davis, A.A., and Temple, S. (2000). Timing of CNS cell generation: a programmed sequence of neuron and glial cell production from isolated murine cortical stem cells. Neuron *28*, 69–80. https://doi.org/10.1016/s0896-6273(00)00086-6.

Robinson, P.N., Köhler, S., Bauer, S., Seelow, D., Horn, D., and Mundlos, S. (2008). The Human Phenotype Ontology: a tool for annotating and analyzing human hereditary disease. Am. J. Hum. Genet. *83*, 610–615. https://doi.org/10.1016/j.ajhg.2008.09.017.

Romano, R., and Bucci, C. (2020). Role of EGFR in the Nervous System. Cells *9*, 1887. https://doi.org/10.3390/cells9081887.

Sakib, M.S., Sokpor, G., Nguyen, H.P., Fischer, A., and Tuoc, T. (2021). Intranuclear immunostaining-based FACS protocol from embryonic cortical tissue. STAR Protoc. *2*, 100318. https://doi.org/10.1016/j.xpro.2021.100318.

Sanai, N., Alvarez-Buylla, A., and Berger, M.S. (2005). Neural stem cells and the origin of gliomas. N. Engl. J. Med. *353*, 811–822. https://doi.org/10.1056/NEJMra043666.

Sherman, B.T., Hao, M., Qiu, J., Jiao, X., Baseler, M.W., Lane, H.C., Imamichi, T., and Chang, W. (2022). DAVID: a web server for functional enrichment analysis and functional annotation of gene lists (2021 update). Nucleic Acids Res. *50*, W216–W221. https://doi.org/10.1093/nar/gkac194.

Singh, S.K., Hawkins, C., Clarke, I.D., Squire, J.A., Bayani, J., Hide, T., Henkelman, R.M., Cusimano, M.D., and Dirks, P.B. (2004). Identification of human brain tumour initiating cells. Nature *432*, 396–401. https://doi.org/10.1038/nature03128.

Sokpor, G., Ulmke, P.A., Nguyen, H.D., Pham, L., Kakani, P., Chu, V.T., Arnold, S.J., Brand-Saberi, B., Nguyen, H.P., and Tuoc, T. (2024). Targeted H3K9 acetylation at lncSox1 promoter by cell type-specific epigenome editing promotes intermediate progenitor proliferation in developing mouse cortex. Preprint at bioRxiv. https://doi.org/10.1101/2024.11.05.621758.

Stahl, R., Walcher, T., De Juan Romero, C., Pilz, G.A., Cappello, S., Irmler, M., Sanz-Aquela, J.M., Beckers, J., Blum, R., Borrell, V., and Götz, M. (2013). Trnp1 regulates expansion and folding of the mammalian cerebral cortex by control of radial glial fate. Cell *153*, 535–549. https://doi.org/10.1016/j.cell.2013.03.027.

Stuart, T., Butler, A., Hoffman, P., Hafemeister, C., Papalexi, E., Mauck, W.M., Hao, Y., Stoeckius, M., Smibert, P., and Satija, R. (2019). Comprehensive Integration of Single-Cell Data. Cell *177*, 1888–1902.e21. https://doi.org/10.1016/j.cell.2019.05.031.

Telley, L., Govindan, S., Prados, J., Stevant, I., Nef, S., Dermitzakis, E., Dayer, A., and Jabaudon, D. (2016). Sequential transcriptional waves direct the differentiation of newborn neurons in the mouse neocortex. Science *351*, 1443–1446. https://doi.org/10.1126/science.aad8361.

Tome-Garcia, J., Tejero, R., Nudelman, G., Yong, R.L., Sebra, R., Wang, H., Fowkes, M., Magid, M., Walsh, M., Silva-Vargas, V., et al. (2017). Prospective Isolation and Comparison of Human Germinal Matrix and Glioblastoma EGFR(+) Populations with Stem Cell Properties. Stem Cell Rep. *8*, 1421–1429. https://doi.org/10.1016/j.stemcr.2017.03.019.

Ulmke, P.A., Sakib, M.S., Ditte, P., Sokpor, G., Kerimoglu, C., Pham, L., Xie, Y., Mao, X., Rosenbusch, J., Teichmann, U., et al. (2021). Molecular Profiling Reveals Involvement of ESCO2 in Intermediate Progenitor Cell Maintenance in the Developing Mouse Cortex. Stem Cell Rep. *16*, 968–984. https://doi.org/10.1016/j.stemcr.2021.03.008.

Verhaak, R.G.W., Hoadley, K.A., Purdom, E., Wang, V., Qi, Y., Wilkerson, M.D., Miller, C.R., Ding, L., Golub, T., Mesirov, J.P., et al. (2010). Integrated genomic analysis identifies clinically relevant subtypes of glioblastoma characterized by abnormalities in PDGFRA, IDH1, EGFR, and NF1. Cancer Cell *17*, 98–110. https://doi.org/10.1016/j.ccr.2009.12.020.

Walton, N.M., Snyder, G.E., Park, D., Kobeissy, F., Scheffler, B., and Steindler, D.A. (2009). Gliotypic neural stem cells transiently adopt tumorigenic properties during normal differentiation. Stem Cell. *27*, 280–289. https://doi.org/10.1634/stemcells.2008-0842.

Wang, H., Chen, H., Zhou, H., Yu, W., and Lu, Z. (2017a). Cyclin-Dependent Kinase Inhibitor 3 Promotes Cancer Cell Proliferation and Tumorigenesis in Nasopharyngeal Carcinoma by Targeting p27. Oncol. Res. *25*, 1431–1440. https://doi.org/10.3727/096504017X14835311718295.

Wang, L., Wang, C., Moriano, J.A., Chen, S., Zuo, G., Cebrián-Silla, A., Zhang, S., Mukhtar, T., Wang, S., Song, M., et al. (2025). Molecular and cellular dynamics of the developing human neocortex. Nature *647*, 169–178. https://doi.org/10.1038/s41586-024-08351-7.

Wang, Q., Hu, B., Hu, X., Kim, H., Squatrito, M., Scarpace, L., deCarvalho, A.C., Lyu, S., Li, P., Li, Y., et al. (2017b). Tumor Evolution of Glioma-Intrinsic Gene Expression Subtypes Associates with

Immunological Changes in the Microenvironment. Cancer Cell *32*, 42–56.e6. https://doi.org/10.1016/j.ccell.2017.06.003.

Wang, X.Q., Lo, C.M., Chen, L., Ngan, E.S.W., Xu, A., and Poon, R.Y. (2017c). CDK1-PDK1-PI3K/Akt signaling pathway regulates embryonic and induced pluripotency. Cell Death Differ. *24*, 38–48. https://doi.org/10.1038/cdd.2016.84.

Yang, R., Wang, M., Zhang, G., Bao, Y., Wu, Y., Li, X., Yang, W., and Cui, H. (2020). E2F7-EZH2 axis regulates PTEN/AKT/mTOR signalling and glioblastoma progression. Br. J. Cancer *123*, 1445–1455. https://doi.org/10.1038/s41416-020-01032-y.

Yoshida, K., Chambers, J.K., and Uchida, K. (2023). Immunohistochemical study of neural stem cell lineage markers in canine brains, gliomas, and a glioma cell line. Vet. Pathol. *60*, 35–46. https://doi.org/10.1177/03009858221136297.

Yu, C., Cao, H., He, X., Sun, P., Feng, Y., Chen, L., and Gong, H. (2017). Cyclin-dependent kinase inhibitor 3 (CDKN3) plays a critical role in prostate cancer via regulating cell cycle and DNA replication signaling. Biomed. Pharmacother. *96*, 1109–1118. https://doi.org/10.1016/j.biopha.2017.11.112.

Yu, H., Li, Z., and Wang, M. (2020a). Expression and prognostic role of E2F transcription factors in high-grade glioma. CNS Neurosci. Ther. *26*, 741–753. https://doi.org/10.1111/cns.13295.

Yu, H., Yao, J., Du, M., Ye, J., He, X., and Yin, L. (2020b). CDKN3 promotes cell proliferation, invasion and migration by activating the AKT signaling pathway in esophageal squamous cell carcinoma. Oncol. Lett. *19*, 542–548. https://doi.org/10.3892/ol.2019.11077.

Zarzor, M.S., Blumcke, I., and Budday, S. (2023). Exploring the role of the outer subventricular zone during cortical folding through a physics-based model. eLife *12*, e82925. https://doi.org/10.7554/eLife.82925.

Zhang, L.P., Li, W.J., Zhu, Y.F., Huang, S.Y., Fang, S.Y., Shen, L., and Gao, Y.L. (2015). CDKN3 knockdown reduces cell proliferation, invasion and promotes apoptosis in human ovarian cancer. Int. J. Clin. Exp. Pathol. *8*, 4535–4544.

Zhong, S., Zhang, S., Fan, X., Wu, Q., Yan, L., Dong, J., Zhang, H., Li, L., Sun, L., Pan, N., et al. (2018). A single-cell RNA-seq survey of the developmental landscape of the human prefrontal cortex. Nature *555*, 524–528. https://doi.org/10.1038/nature25980.

STAR★METHODS

KEY RESOURCES TABLE

REAGENT or RESOURCE	SOURCE	IDENTIFIER
**Antibodies**		
Mouse monoclonal human EOMES/TBR2 (for FACS from human tissue)	R and D Systems	Cat#IC6166G; RRID: AB_3656306
Rabbit monoclonal mouse EOMES/TBR2 (for FACS from mouse tissue)	Abcam	Cat# ab183991; RRID: AB_2721040
Rat monoclonal EOMES/TBR2 (1:200)	Thermo Fisher Scientific	Cat#14-4875-80; RRID: AB_11043546
Rabbit polyclonal EOMES/TBR2 (1:200)	Abcam	Cat#ab23345; RRID: AB_778267
Chicken polyclonal GFP (1:400)	Abcam	Cat#ab13970; RRID: AB_300798
Rabbit polyclonal mCherry/RFP (1:1000)	Rockland	Cat#600-401-379; RRID: AB_2209751
Mouse monoclonal mCherry/RFP (1:1000)	Rockland	Cat#200-301-379; RRID: AB_2611063
Rabbit polyclonal human CDKN3 (1:200)	Abcam	Cat#ab175393; RRID: AB_3665380
Mouse monoclonal NeuN (1:200)	Chemicon	Cat#MAB377; RRID: AB_2298772
Mouse monoclonal HuC/HuD (HuCD) (1:20)	Thermo Fisher Scientific	Cat#A-21271; RRID:AB_221448
Mouse monoclonal TUBB3 (Tuj1) (1:200)	Millipore	Cat#MAB1637; RRID: AB_2210524
Mouse monoclonal pHH3 (1:50)	Cell Signaling	Cat#9706; RRID: AB_331748
Rat monoclonal BrdU (1:100)	Abcam	Cat#ab6326; RRID: AB_305426
Mouse monoclonal BrdU (1:40)	BD Biosciences	Cat#347580; RRID: AB_10015219
Goat Anti-Rabbit IgG Antibody, Alexa Fluor 405 Conjugated	Molecular Probes	Cat# A-31556; RRID: AB_221605
Goat Anti-Mouse IgG1 Antibody, Alexa Fluor 488 Conjugated	Molecular Probes	Cat#A-21121; RRID: AB_2535764
Goat Anti-Rabbit IgG Antibody, Alexa Fluor 488 Conjugated	Molecular Probes	Cat#A-11008; RRID:AB_143165
Goat Anti-Chicken IgG Antibody, Alexa Fluor 488 Conjugated	Molecular Probes	Cat#A-11039; RRID: AB_142924
Goat Anti-Rat IgG Antibody, Alexa Fluor 488 Conjugated	Molecular Probes	Cat#A-11006; RRID:AB_141373
Goat Anti-Mouse IgG1 Antibody, Alexa Fluor 568 Conjugated	Molecular Probes	Cat#A-21124; RRID:AB_141611
Goat Anti-Rat IgG Antibody, Alexa Fluor 568 Conjugated	Molecular Probes	Cat#A-11077; RRID: AB_141874
Donkey Anti-Rabbit IgG Antibody, Alexa Fluor 555 Conjugated	Molecular Probes	Cat#A-31572; RRID: AB_162543
Goat Anti-Mouse IgG1 Antibody, Alexa Fluor 647 Conjugated	Molecular Probes	Cat#A-21240; RRID: AB_141658
Goat Anti-Rat IgG Antibody, Alexa Fluor 647 Conjugated	Molecular Probes	Cat#A-21247; RRID: AB_141778

(*Continued on next page*)

Continued

REAGENT or RESOURCE	SOURCE	IDENTIFIER
ImmPRESS HRP Anti-Mouse Ig Reagent	Vector Laboratories	Cat#MP-7402; RRID: AB_2336528
ImmPRESS HRP Anti-Rabbit Ig Reagent	Vector Laboratories	Cat#MP-7451; RRID: AB_2631198
**Biological samples**		
Human fetal brain tissue	Hospital for Obstetrics and Gynecology "Prof. Dimitar Stamatov," Medical University Varna (MUV)	N/A
Human fetal brain tissue	MRC/Wellcome Trust-funded Human Developmental Biology Resource (HDBR)	http://www.hdbr.org
**Chemicals, peptides, and recombinant proteins**		
TRIzol LS	Thermo Fisher Scientific	Cat#10296010
PureLink PCR Purification Kit spin columns	Thermo Fisher Scientific	Cat#K310002
Hyb-mix (Hybridization buffer)	Ambion	Cat#B8807G
NBT/BCIP (Roche)	Sigma-Aldrich	Cat#11681451001
**Critical commercial assays**		
Zymo RNA clean & concentrator-5 kit	Zymo Research	Cat#R1013
Takara SMART-Seq v4 Ultra Low Input RNA kit	Takara Bio	Cat#R400752
**Deposited data**		
RNA-seq data human IPCs; RNA-seq data hCDKN3OE mouse IPCs	This paper	BioProject: PRJNA1213291
RNA-seq data mouse IPCs	Ulmke et al. (2021)	GEO: GSE168298
GBM data (EGFR+ and EGFR– populations from germinal matrix (GM) and glioblastoma)	Tome-Garcia et al. (2017)	GEO: GSE96682
Human cortex scRNA-seq/ scATAC-seq dataset	Wang et al. (2025)	NeMO ID: nemo:dat-oiif74w
Mouse cortex scRNA-seq/ scATAC-seq dataset	Liu et al. (2025)	GEO: GSE241429
**Experimental models: Organisms/strains**		
Tbr2-2A-CreER mice, strain #:036301	The Jackson Laboratory	RRID:IMSR_JAX:036301
**Oligonucleotides**		
Primers for CDKN2A, CDKN3, DEPDC1, E2F7, ERFE, FAM111B, FOXD1, GLI1, BTN1A1, HASPIN, INSM2, KIF19, NEK2, NKX3.2, NT5DC4, PAX4, PKN3, PMAIP1, SDC4, TONSL, VSX1 and WDR62, see Table S8	This paper	N/A
**Recombinant DNA**		
pCAG-LoxP-mCherry-PolyA-LoxP-hCDKN3-T2A-eGFP	This paper	N/A
**Software and algorithms**		

(*Continued on next page*)

Continued		
REAGENT or RESOURCE	SOURCE	IDENTIFIER
Adobe Photoshop	Adobe	RRID: SCR_014199
ImageJ	https://imagej.net/	RRID: SCR_003070
Galaxy web platform	http://galaxyproject.org/	RRID: SCR_006281
fasterq-dump tool (SRA Toolkit v3.0.3)	https://github.com/ncbi/sra-tools/	RRID: SCR_024350
FastQC	http://www.bioinformatics.babraham.ac.uk/projects/fastqc/	RRID: SCR_014583
HISAT2	http://ccb.jhu.edu/software/hisat2/index.shtml	RRID:SCR_015530
featureCounts	http://bioinf.wehi.edu.au/featureCounts/	RRID: SCR_012919
DeSeq2	https://bioconductor.org/packages/release/bioc/html/DESeq2.html	RRID: SCR_015687
R	http://www.r-project.org/	RRID: SCR_001905
Seurat	https://satijalab.org/seurat/get_started.html	RRID: SCR_016341
Harmony	https://github.com/immunogenomics/harmony	RRID: SCR_022206
biomaRt	https://bioconductor.org/packages/biomaRt/	RRID: SCR_019214
rtracklayer	https://bioconductor.org/packages/rtracklayer	RRID: SCR_021325
DAVID	https://david.ncifcrf.gov/	RRID: SCR_001881

EXPERIMENTAL MODEL AND STUDY PARTICIPANT DETAILS

Human fetal brain

Human fetal brain tissue was obtained from spontaneous abortions that occurred in the Hospital for Obstetrics and Gynecology "Prof. Dimitar Stamatov," Medical University Varna (MUV), Bulgaria, after an informed written maternal consent and following the approval of the MUV ethics committee (Protocol #55/June 2016). The gestational week (GW) has been determined on the basis of the history of last menstruation as reported by the patients.

Additional paraffin sections of human fetal brain were obtained through the MRC/Wellcome Trust-funded Human Developmental Biology Resource (HDBR, http://www.hdbr.org) which collects fetal tissue from terminated pregnancies. The Newcastle and North Tyneside NHS Health Authority Joint Ethics Committee authorized the collection of all tissue samples obtained with the mother's informed consent.

Transgenic mice

Tbr2/Eomes-CreER mice (Matho et al., 2021), JAX stock #036301, were maintained in a C57BL6/J background. Animals were handled according to the German Animal Protection Law.

METHOD DETAILS

TBR2+ nuclei sorting from embryonic human cortex

The method was adapted from a previously reported protocol (Sakib et al., 2021). Human fetal cortical tissue (GW14–17) was mechanically homogenized in ice-cold EZ Prep Lysis Buffer (Sigma) using a plastic pestle, followed by sequential lysis and filtration through a 70 μm cell strainer to remove debris and tissue aggregates. Nuclei were washed and resuspended in

nuclei suspension buffer containing PBS, BSA, RNase inhibitor, and protease inhibitor.

For intranuclear immunostaining, isolated nuclei were incubated with an anti-TBR2 antibody (R&D Systems, IC6166G; 1:15 dilution) for 50 min at 4°C with gentle rotation. After washing, nuclei were incubated with an appropriate fluorescent secondary antibody for 30 min at 4°C, followed by an additional wash step. Nuclei were subsequently filtered through a 40 μm cell strainer and subjected to sorting on a MoFlo Astrios EQ from Beckman Coulter. Debris and aggregates were excluded based on forward and side scatter properties, and singlet nuclei were selected by sequential gating. Sorted nuclei were collected into nuclei suspension buffer, distributed equally into four aliquots, which were processed independently as technical replicates, and processed for RNA isolation.

TBR2+/GFP+ cell sorting from mouse cortex

After *in utero* electroporation (described later in discussion) using a human *CDKN3* overexpression plasmid at E13.5, mouse cortices were collected at E15.5. To preserve GFP signal, whole cells (instead of nuclei) were isolated and processed for immunostaining of the nuclear marker TBR2 under RNA-preserving conditions.

First, cortices were dissociated in 1 mL Accutase for 5 min at 37°C with gentle agitation every 1–2 min, followed by centrifugation at 500 × g for 5 min at 4°C. Pellets were resuspended in 1 mL trituration buffer (PBS containing 1% BSA, 0.1–0.2 mg/mL DNase I, and RNase inhibitor) and triturated gently 20–30 times. The suspension was layered over 4 mL of 22% (v/v) Percoll and centrifuged at 500 × g for 10 min at 4°C to remove myelin and debris. Pellets were resuspended in PBS.

Cells were fixed in 1% PFA (final concentration) for 10 min at room temperature and quenched with 0.2 M glycine for 5 min. After centrifugation (1500 × g, 5 min, 4°C), cells were washed once in saponin buffer (PBS with 1% BSA, 0.1% saponin, 1 mM EDTA, and RNase inhibitor) and blocked for 10 min at 4°C in saponin buffer supplemented with 5% normal goat serum (NGS). Primary antibody incubation was performed for 50 min at 4°C with rabbit anti-TBR2 (Abcam, ab183991, 1:50). After washing once in saponin buffer, cells were incubated with Alexa 405-conjugated goat anti-rabbit secondary antibody (Invitrogen, A31556; 1:1000) for 30 min at 4°C in saponin buffer containing 5% NGS. Cells were washed once in sort buffer (PBS with 1% BSA, 2 mM EDTA, and RNase inhibitor) and passed through a 30 μm cell strainer. Cells were sorted with 405 nm and 488 nm excitation to isolate GFP+/TBR2+ and GFP–/TBR2+ fractions. Sorted cells were pelleted (1500 × g, 5 min, 4°C) and subjected to crosslink reversal by incubation in 100 μL of CR buffer (10 mM Tris-HCl, pH 7.5–8.0, 0.5% SDS, 1 mM EDTA, 0.5 mg/mL Proteinase K) for 45 min at 56°C–60°C followed by 30 min at 65°C. RNA was then extracted.

RNA-sequencing and bioinformatics analysis

RNA from sorted human nuclei and sorted mouse cells was purified using TRIzol LS and aqueous phase cleanup using Zymo RNA clean and concentrator-5 kit. RNA concentration was determined using a Qubit fluorometer (Thermo Fisher Scientific), and RNA integrity was assessed using an Agilent TapeStation system (Agilent Technologies). RNA-seq libraries were created utilizing the Takara SMART-Seq v4 Ultra Low Input RNA kit using 2 ng of RNA. Libraries were sequenced using Illumina NextSeq1000 (paired end, read lengths 50 bps).

The sequencing data were uploaded to the Galaxy web platform, and we used the public server at usegalaxy.org to analyze the data (Galaxy, 2024). Quality control was performed using FastQC (http://www.bioinformatics.babraham.ac.uk/projects/fastqc/). Reads were aligned to the human genome hg38, or the mouse genome mm10 respectively, using HISAT2 (Kim et al., 2015) and counted using featureCounts (Liao et al., 2014). DeSeq2 was used to determine differently expressed genes by calculating log2 Fold Changes (FC) from the count tables (Love et al., 2014). For GO and KEGG pathways analyses, the Database for Annotation, Visualization, and Integrated Discovery (DAVID) was used (Huang da et al., 2009; Sherman et al., 2022).

In utero electroporation (IUE)

Timed-pregnant mice were anesthetized by intraperitoneal injection of ketamine/xylazine (100/10 mixture, 0.1 mg per g body weight). Following laparotomy, uterine horns were carefully exposed and maintained under sterile conditions using humidified gauze pads. Endotoxin-free plasmid DNA was prepared using the EndoFree Plasmid Kit (Qiagen) and injected at a concentration of 2.5 μg/μL into the lateral ventricles of embryonic mouse brains together with 0.05% Fast Green (Sigma-Aldrich) to visualize successful injection. DNA solutions contained either a human *CDKN3* overexpression plasmid or an empty mCherry-eGFP plasmid as a control.

Electroporation was performed using platinum tweezer electrodes (CUY650P3, BEX Co., Ltd.) connected to an ECM-830 square-wave electroporator (BTX). Five electrical pulses of 30–35 V were applied to target dorsal cortical progenitors.

Following electroporation, uterine horns were returned to the abdominal cavity, the incision was sutured, and animals were allowed to recover on a heating pad before being returned to their home cage.

Embryos were collected at the developmental stages indicated in the respective experiments and processed for histological, molecular, or cell-sorting analyses.

Immunohistochemistry (IHC)

Frozen sections were allowed to dry at room temperature and washed in PBS for 10–60 min. Tissue sections were outlined using an ImmEdge hydrophobic barrier pen (Vector Laboratories) and blocked for 1 h at room temperature in blocking solution consisting of 5% normal goat serum (Biozol) and 0.1% Triton X-100 in PBS. Primary antibodies were diluted in blocking solution and applied overnight at 4°C in a humidified chamber.

Following primary antibody incubation, sections were washed twice in PBS for 15 min and incubated with species-appropriate Alexa Fluor-conjugated secondary antibodies (Invitrogen; 1:400) diluted in blocking solution for 2 h at room temperature. After two 30-min washes in PBS, sections were either incubated with a second primary antibody overnight at 4°C and processed analogously on the following day, or counterstained with DAPI and mounted. For nuclear staining, DAPI was applied for 5 min prior to mounting. Sections were mounted in Vectashield mounting medium containing DAPI or propidium iodide (Vector Laboratories) and stored at 4°C in the dark until imaging.

Fluorescence images were obtained by confocal fluorescence microscopy (TCS SP5, Leica) and analyzed using an Axio Imager M2 (Zeiss) with a Neurolucida system. Images were processed further using Adobe Photoshop.

Ratio of S-phase determination

Ratio of S-phase IPCs was determined on coronal sections of E15.5 brains 1h after intraperitoneal injection of BrdU (140 μg/g) of pregnant mice. After double IHC with BrdU and TBR2 antibodies, the ratio of S-phase IPCs was calculated as the number of TBR2+BrdU + cells divided by total TBR2+ cells.

Human fetal brain processing for ISH

After collection of a human fetus, the head was placed in 4% paraformaldehyde (PFA) solution in phosphate-buffered saline (PBS, pH 7.5) for 24 h, and then the brain was dissected and postfixed in PFA for 5 to 7 days, cryo-protected in sucrose, and frozen in optimal cutting temperature medium before cryosectioning at 20 μm.

In situ hybridization validation (ISH)

For ISH staining, templates were generated from cDNA obtained by reverse transcription of mRNA from human fetal cortical tissue. PCR with specific forward and reverse primers extended at their 5′ end with either T7 or SP6 promoter sequences were used (Table S8). Temperature gradient PCR conditions (55°C–65°C) were adjusted at: 3 min initial template denaturation at 94°C, 35 cycles with 30 s denaturation (94°C), 30 s primer annealing (55°C–65°C), 1 min per kilobase of elongation (72°C), and a final elongation cycle for 9 min at 72°C. The resulting product was analyzed and re-amplified via PCR as described above, except that primer annealing temperature was the one determined to be optimal in the gradient PCR. Templates were purified using the PureLink PCR Purification Kit spin columns (ThermoFisher).

Digoxigenin-labeled riboprobes were generated by *in vitro* transcription. The synthetized RNA was precipitated in ethanol, re-dissolved in 50 μL of DEPC water while vigorously shaking at room temperature. 2 μL of the RNA were analyzed on a 1% agarose gel, and 1 μL were used to determine the concentration on a spectrophotometer. Probes were diluted with Hyb-mix (Ambion, catalog no. B8807G) to a final concentration of 100–400 ng/mL. Hybridized probes were detected with a two-step chromogenic catalyzed reporter deposition using anti-DIG-AP antibody and NBT/BCIP (Roche).

scRNA-seq dataset and deconvolution

For comparison to our data from bulk RNA-seq, scRNA-seq data from 38 human neocortical samples across different developmental stages (Wang et al., 2025) was used, available in an interactive portal (https://cell.ucsf.edu/snMultiome) and at Dryad (https://doi.org/10.5061/dryad.2280gb612). Deconvolution of the bulk RNA-seq data was carried out using the CIBERSORT algorithm (Newman et al., 2015), which infers cell type proportions based on gene expression profiles. Count matrices from TBR2+ and TBR2− bulk RNA-seq replicates were used as input, and single-cell data (NeMO ID: nemo:dat-oiif74w) of the Wang et al. dataset served as the reference expression matrix for the deconvolution analysis.

Cross-species scRNA-seq and scATAC-seq analysis

Single-cell multiome (gene expression and chromatin accessibility) datasets from developing human and mouse cortex were obtained from publicly available studies. For mouse, dataset GSE241429 (Liu et al., 2025) was used. For human, dataset NeMO ID: nemo:dat-oiif74w (Wang et al., 2025) was used. To enable cross-species comparison, an orthologous gene list between human and mouse was generated using biomaRt (Durinck et al., 2009) based on the Ensembl December 2021 archive. Only one-to-one orthologs were retained to ensure unambiguous mapping between species, and RNA count matrices from both datasets were subset to the shared orthologous gene set prior to integration.

For ATAC processing, mouse ATAC peak coordinates and corresponding fragment files (mm10) were converted to the human genome assembly (hg38) using the liftOver function implemented in the rtracklayer package (Lawrence et al., 2009) together with the mm10ToHg38 chain file. Only successfully mapped regions were retained. ATAC peak sets from both species were subsequently intersected to generate a shared genomic feature space for cross-species comparison.

Datasets were integrated at the RNA level using SCTransform normalization followed by FindIntegrationAnchors and IntegrateData implemented in Seurat (Stuart et al., 2019). Chromatin accessibility datasets were harmonized using Harmony (Korsunsky et al., 2019) to correct for batch effects while preserving biological variation. To enable cross-species comparisons, mouse cell-type annotations were mapped to corresponding human cell-type categories, consolidating mouse neuronal subclasses into broader classes consistent with the human dataset. Specifically, mouse radial glia (RG) were mapped to radial glia, interneurons (In) to GABAergic neurons, glutamatergic projection neuron subclasses (MGN, CPN, CThPN, SCPN) to glutamatergic neurons, intermediate progenitors (IP) to IPC-EN populations, endothelial and pericyte cells (End, Per) to vascular cells, and oligodendrocyte lineage cells (OPC, oligodendrocyte) to IPC-Glia populations. Chromatin accessibility at the *CDKN3* locus was then examined across annotated progenitor populations to assess species-specific regulatory differences.

All analyses were performed in R (Dessau and Pipper, 2008) using the Seurat, Harmony, biomaRt, and rtracklayer packages.

Glioblastoma datasets

To analyze gene expression in human glioblastoma multiforme (GBM), transcriptomic data were obtained from the Gene Expression Omnibus (GEO) database with the dataset ID GSE96682. The dataset contains 12 samples: EGFR+ and EGFR– populations from human brain germinal matrix resections (GM, $N = 3$ independent human samples) and from human glioblastoma resections (GBM, $N = 3$ independent human samples). The FASTQ files were downloaded from GEO using the fasterq-dump tool (SRA Toolkit v3.0.3), followed by RNA-seq analysis as described above.

QUANTIFICATION AND STATISTICAL ANALYSIS

General quantification and statistical analysis

Quantitative data are presented as mean ± SD. Sample sizes are reported in the corresponding figure legends and refer to the experimental units analyzed in each assay (e.g., embryos, images, or independently prepared RNA-seq libraries). Statistical tests are indicated in the corresponding figure legends, and detailed statistical analyses are provided in Table S7.

Statistical analysis of gene set overlap

To assess the statistical significance of gene overlap between TBR2+ hIPCs and various GBM subtypes, random sampling and cumulative distribution function (CDF) calculation was performed. 1000 iterations of random sampling were conducted for each comparison, selecting gene sets of the same size as the GBM gene set and calculating their overlap with the TBR2+ hIPC-upregulated gene set. The resulting overlaps were used to construct a CDF, representing the probability of observing a given number of overlapping genes by chance. We compared our observed gene overlap to this CDF to calculate p-values, with $p < 0.001$ indicating that none of the 1000 random iterations reached the observed overlap, suggesting high statistical significance. The statistics analysis of gene set overlap are presented in Table S4.

Stem Cell Reports, Volume *21*

Supplemental Information

Identification of novel genes with enriched expression in human intermediate progenitors reveals a key role for CDKN3 in cortical development

Pauline Antonie Ulmke, Martin N. Ivanov, Hoang Duy Nguyen, Linh Pham, Asisa Muchamedin, Ayman Alzu'bi, Lora V. Veleva, Tsvetomir Kachovski, Xiaoyi Mao, Krzysztof P. Lubieniecki, Emil Kovachev, Gavin J. Clowry, Anton B. Tonchev, Huu Phuc Nguyen, and Tran Tuoc

Supplemental Figures

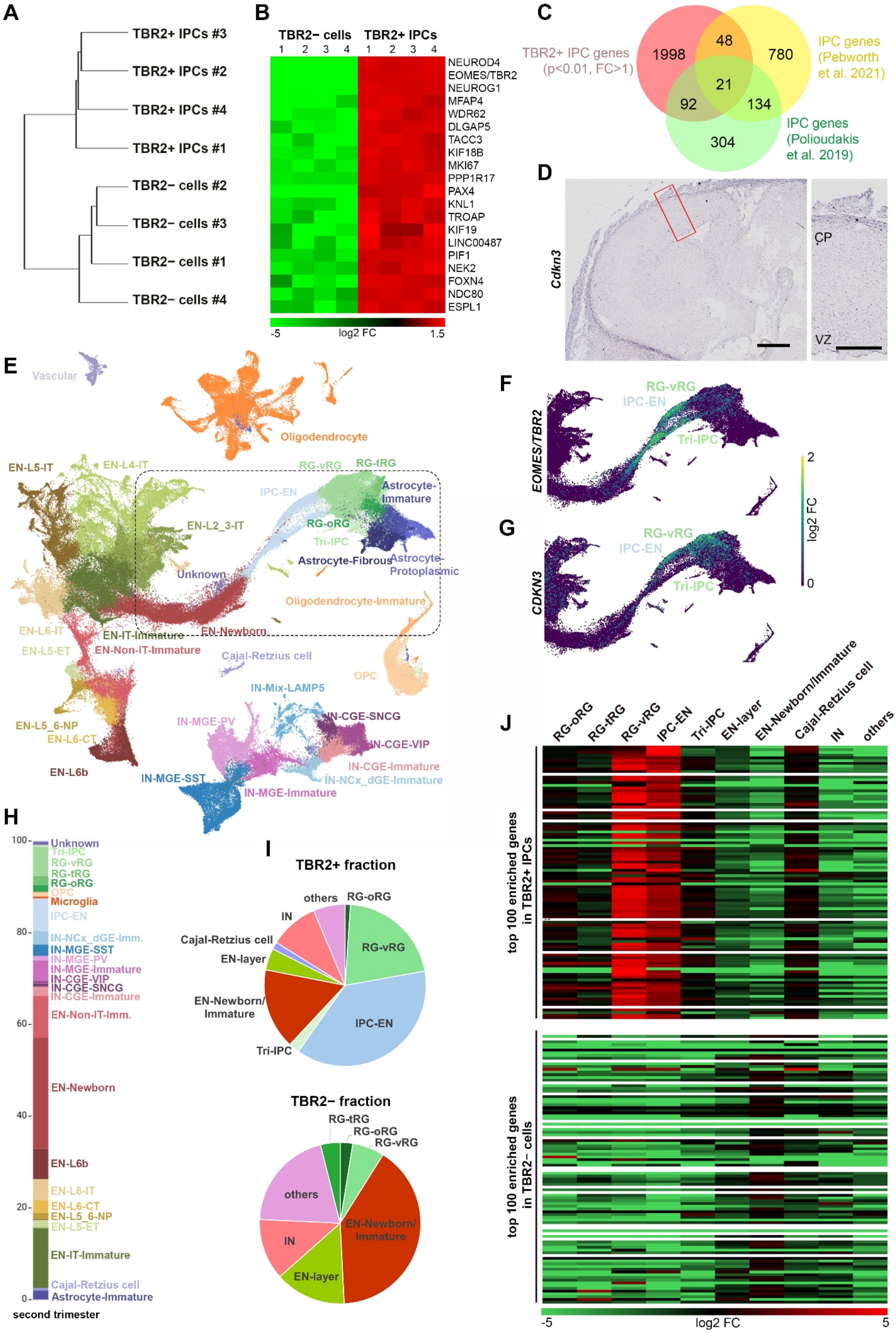

Figure S1. RNA-seq analysis of human TBR2+ IPCs and TBR2− nuclei in the context with scRNA-seq data.

(A) Cluster dendrogram analysis of RNA-seq for human TBR2+ and TBR2− sorted nuclei samples (*N* = 4 RNA-seq libraries per condition). **(B)** Heatmap showing the top 20 genes with high expression (enriched/upregulated) in TBR2+ nuclei and low expression in TBR2− nuclei. **(C)** Overlap of genes upregulated in hIPCs in this study (p-value < 0.01, log2FC > 1) and IPC genes identified in previous studies. **(D)** *In situ* hybridization for *Cdkn3* in the E14.5 mouse brain. Magnified cortical region is shown by a red box. **(E)** UMAP plots of the scRNA-seq data from Wang el al., 2025 showing the distribution of cell types from 38 human neocortical samples across different developmental stages. **(F, G)** UMAP plots of the scRNA-seq data from Wang el al. showing expression levels of *EOMES/TBR2* (F) and *CDKN3* (G). Expression values are displayed as log2-transformed normalized UMI counts, plotted relative to the mean expression across all cells in the dataset. **(H)** Cell composition of the second-trimester cortex (GW13–23) identified by Wang et al. **(I)** Pie chart showing cell type proportions of TBR2+ hIPCs and TBR2− cells after deconvolution with scRNA-seq data of the Wang et al. dataset. **(J)** Heatmap showing the top 100 genes with high expression in TBR2+ nuclei and the top 100 genes with high expression in TBR2− nuclei in cell types identified in the scRNA-seq from Wang el al. VZ, ventricular zone; CP, cortical plate; oRG, outer radial glia; tRG, truncated RG; vRG; ventricular RG, IPC-EN, intermediate progenitors for excitatory neurons; Tri-IPC, tripotent IPC; IT, intratelencephalic; L, layer; IN, interneurons; Imm., immature; IN-NCx_dGE-Immature, neocortex and dorsal ganglionic eminence-derived immature INs; CGE, caudal ganglionic eminence; SNCG, synuclein-gamma; VIP, vasoactive intestinal polypeptide; MGE, medial ganglionic eminence; PV, parvalbumin; SST, somatostatin; LAMP5, Lysosomal Associated Membrane Protein Family Member 5; OPC, oligodendrocyte precursor cells. Scale bars: 500 μm, 250 μm (higher magnification).

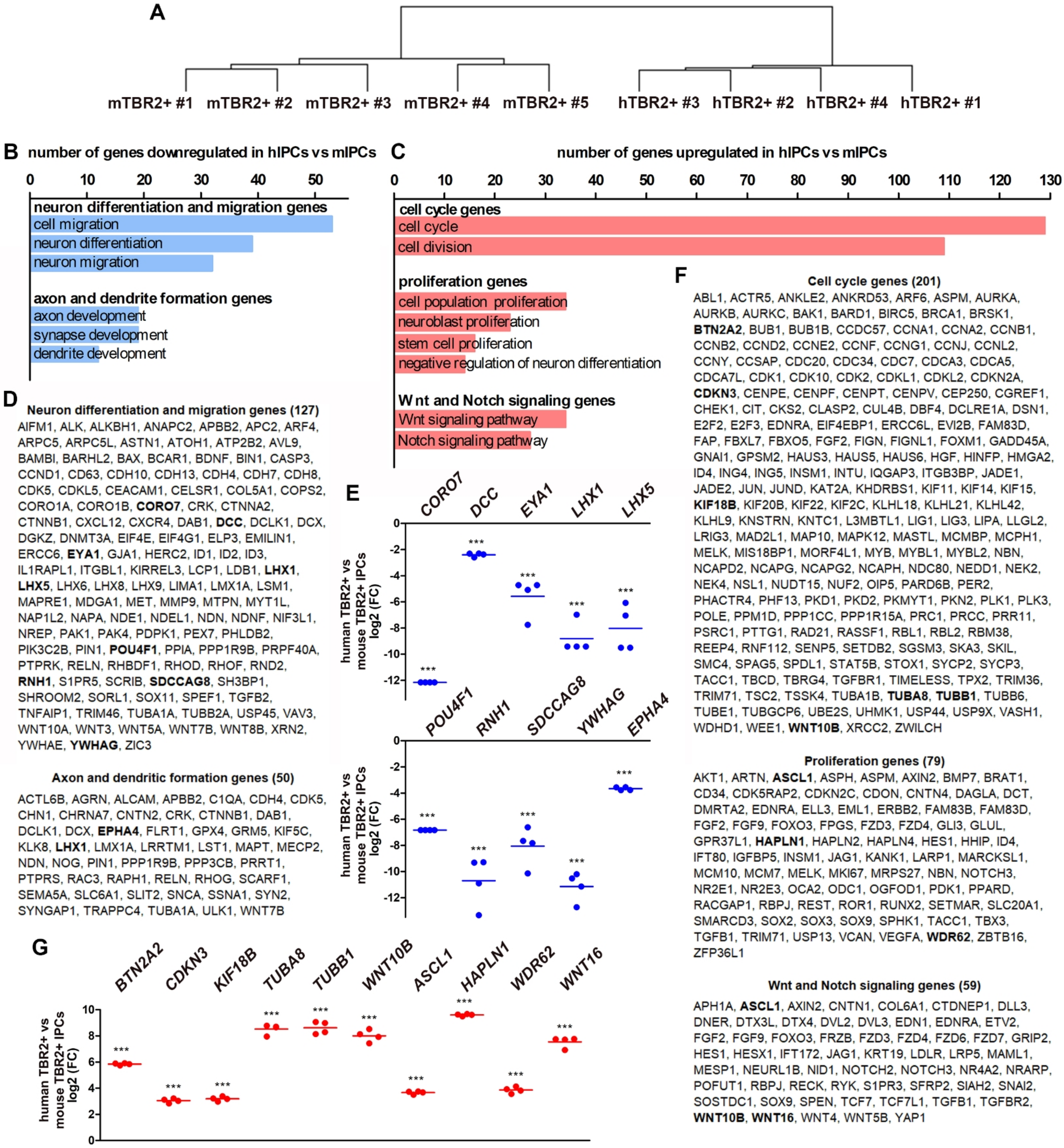
A
mTBR2+ #1
mTBR2+ #2
mTBR2+ #3
mTBR2+ #4
mTBR2+ #5
hTBR2+ #3
hTBR2+ #2
hTBR2+ #4
hTBR2+ #1
B
number of genes downregulated in hIPCs vs mIPCs
0 10 20 30 40 50
neuron differentiation and migration genes
cell migration
neuron differentiation
neuron migration
axon and dendrite formation genes
axon development
synapse development
dendrite development
C
number of genes upregulated in hIPCs vs mIPCs
0 10 20 30 40 50 60 70 80 90 100 110 120 130
cell cycle genes
cell cycle
cell division
proliferation genes
cell population proliferation
neuroblast proliferation
stem cell proliferation
negative regulation of neuron differentiation
Wnt and Notch signaling genes
Wnt signaling pathway
Notch signaling pathway
D
Neuron differentiation and migration genes (127)
AIFM1, ALK, ALKBH1, ANAPC2, APBB2, APC2, ARF4, ARPC5, ARPC5L, ASTN1, ATOH1, ATP2B2, AVL9, BAMBI, BARHL2, BAX, BCAR1, BDNF, BIN1, CASP3, CCND1, CD63, CDH10, CDH13, CDH4, CDH7, CDH8, CDK5, CDKL5, CEACAM1, CELSR1, COL5A1, COPS2, CORO1A, CORO1B, CORO7, CRK, CTNNA2, CTNNB1, CXCL12, CXCR4, DAB1, DCC, DCLK1, DCX, DGKZ, DNMT3A, EIF4E, EIF4G1, ELP3, EMILIN1, ERCC6, EYA1, GJA1, HERC2, ID1, ID2, ID3, IL1RAPL1, ITGBL1, KIRREL3, LCP1, LDB1, LHX1, LHX5, LHX6, LHX8, LHX9, LIMA1, LMX1A, LSM1, MAPRE1, MDGA1, MET, MMP9, MTPN, MYT1L, NAP1L2, NAPA, NDE1, NDEL1, NDN, NDNF, NIF3L1, NREP, PAK1, PAK4, PDPK1, PEX7, PHLDB2, PIK3C2B, PIN1, POU4F1, PPIA, PPP1R9B, PRPF40A, PTPRK, RELN, RHBDF1, RHOD, RHOF, RND2, RNH1, S1PR5, SCRIB, SDCCAG8, SH3BP1, SHROOM2, SORL1, SOX11, SPEF1, TGFB2, TNFAIP1, TRIM46, TUBA1A, TUBB2A, USP45, VAV3, WNT10A, WNT3, WNT5A, WNT7B, WNT8B, XRN2, YWHAE, YWHAG, ZIC3
Axon and dendritic formation genes (50)
ACTL6B, AGRN, ALCAM, APBB2, C1QA, CDH4, CDK5, CHN1, CHRNA7, CNTN2, CRK, CTNNB1, DAB1, DCLK1, DCX, EPHA4, FLRT1, GPX4, GRM5, KIF5C, KLK8, LHX1, LMX1A, LRRTM1, LST1, MAPT, MECP2, NDN, NOG, PIN1, PPP1R9B, PPP3CB, PRRT1, PTPRS, RAC3, RAPH1, RELN, RHOG, SCARF1, SEMA5A, SLC6A1, SLIT2, SNCA, SSNA1, SYN2, SYNGAP1, TRAPPC4, TUBA1A, ULK1, WNT7B
E
CORO7
DCC
EYA1
LHX1
LHX5
human TBR2+ vs mouse TBR2+ IPCs log2 (FC)
0 -2 -4 -6 -8 -10 -12
***
POU4F1
RNH1
SDCCAG8
YWHAG
EPHA4
human TBR2+ vs mouse TBR2+ IPCs log2 (FC)
0 -2 -4 -6 -8 -10 -12
***
F
Cell cycle genes (201)
ABL1, ACTR5, ANKLE2, ANKRD53, ARF6, ASPM, AURKA, AURKB, AURKC, BAK1, BARD1, BIRC5, BRCA1, BRSK1, BTN2A2, BUB1, BUB1B, CCDC57, CCNA1, CCNA2, CCNB1, CCNB2, CCND2, CCNE2, CCNF, CCNG1, CCNJ, CCNL2, CCNY, CCSAP, CDC20, CDC34, CDC7, CDCA3, CDCA5, CDCA7L, CDK1, CDK10, CDK2, CDKL1, CDKL2, CDKN2A, CDKN3, CENPE, CENPF, CENPT, CENPV, CEP250, CGREF1, CHEK1, CIT, CKS2, CLASP2, CUL4B, DBF4, DCLRE1A, DSN1, E2F2, E2F3, EDNRA, EIF4EBP1, ERCC6L, EVI2B, FAM83D, FAP, FBXL7, FBXO5, FGF2, FIGN, FIGNL1, FOXM1, GADD45A, GNAI1, GPSM2, HAUS3, HAUS5, HAUS6, HGF, HINFP, HMGA2, ID4, ING4, ING5, INSM1, INTU, IQGAP3, ITGB3BP, JADE1, JADE2, JUN, JUND, KAT2A, KHDRBS1, KIF11, KIF14, KIF15, KIF18B, KIF20B, KIF22, KIF2C, KLHL18, KLHL21, KLHL42, KLHL9, KNSTRN, KNTC1, L3MBTL1, LIG1, LIG3, LIPA, LLGL2, LRIG3, MAD2L1, MAP10, MAPK12, MASTL, MCMBP, MCPH1, MELK, MIS18BP1, MORF4L1, MYB, MYBL1, MYBL2, NBN, NCAPD2, NCAPG, NCAPG2, NCAPH, NDC80, NEDD1, NEK2, NEK4, NSL1, NUDT15, NUF2, OIP5, PARD6B, PER2, PHACTR4, PHF13, PKD1, PKD2, PKMYT1, PKN2, PLK1, PLK3, POLE, PPM1D, PPP1CC, PPP1R15A, PRC1, PRCC, PRR11, PSRC1, PTTG1, RAD21, RASSF1, RBL1, RBL2, RBM38, REEP4, RNF112, SENP5, SETDB2, SGSM3, SKA3, SKIL, SMC4, SPAG5, SPDL1, STAT5B, STOX1, SYCP2, SYCP3, TACC1, TBCD, TBRG4, TGFBR1, TIMELESS, TPX2, TRIM36, TRIM71, TSC2, TSSK4, TUBA1B, TUBA8, TUBB1, TUBB6, TUBE1, TUBGCP6, UBE2S, UHMK1, USP44, USP9X, VASH1, WDHD1, WEE1, WNT10B, XRCC2, ZWILCH
Proliferation genes (79)
AKT1, ARTN, ASCL1, ASPH, ASPM, AXIN2, BMP7, BRAT1, CD34, CDK5RAP2, CDKN2C, CDON, CNTN4, DAGLA, DCT, DMRTA2, EDNRA, ELL3, EML1, ERBB2, FAM83B, FAM83D, FGF2, FGF9, FOXO3, FPGS, FZD3, FZD4, GLI3, GLUL, GPR37L1, HAPLN1, HAPLN2, HAPLN4, HES1, HHIP, ID4, IFT80, IGFBP5, INSM1, JAG1, KANK1, LARP1, MARCKSL1, MCM10, MCM7, MELK, MKI67, MRPS27, NBN, NOTCH3, NR2E1, NR2E3, OCA2, ODC1, OGFOD1, PDK1, PPARD, RACGAP1, RBPJ, REST, ROR1, RUNX2, SETMAR, SLC20A1, SMARCD3, SOX2, SOX3, SOX9, SPHK1, TACC1, TBX3, TGFB1, TRIM71, USP13, VCAN, VEGFA, WDR62, ZBTB16, ZFP36L1
Wnt and Notch signaling genes (59)
APH1A, ASCL1, AXIN2, CNTN1, COL6A1, CTDNEP1, DLL3, DNER, DTX3L, DTX4, DVL2, DVL3, EDN1, EDNRA, ETV2, FGF2, FGF9, FOXO3, FRZB, FZD3, FZD4, FZD6, FZD7, GRIP2, HES1, HESX1, IFT172, JAG1, KRT19, LDLR, LRP5, MAML1, MESP1, NEURL1B, NID1, NOTCH2, NOTCH3, NR4A2, NRARP, POFUT1, RBPJ, RECK, RYK, S1PR3, SFRP2, SIAH2, SNAI2, SOSTDC1, SOX9, SPEN, TCF7, TCF7L1, TGFB1, TGFBR2, WNT10B, WNT16, WNT4, WNT5B, YAP1
G
BTN2A2
CDKN3
KIF18B
TUBA8
TUBB1
WNT10B
ASCL1
HAPLN1
WDR62
WNT16
human TBR2+ vs mouse TBR2+ IPCs log2 (FC)
0 2 4 6 8 10
***

Figure S2. Upregulation of proliferation and cell cycle genes and downregulation of neuronal differentiation genes in hIPCs versus mIPCs.

(**A**) Cluster dendrogram analysis of RNA-seq for mouse TBR2+ sorted nuclei (*N* = 5 RNA-seq libraries per condition) and human TBR2+ sorted nuclei (*N* = 4 RNA-seq libraries per condition). (**B, C**) Gene Ontology analyses of genes downregulated in hIPCs versus mIPCs (B), and genes upregulated in hIPCs versus mIPCs (C). **(D, E)** List of genes downregulated in hIPCs versus mIPCs that functionally fall under neuron differentiation and migration and axon and dendritic formation (D) and the expression levels of selected genes (highlighted bold in the adjoining gene list) as identified in the individual samples of hIPCs versus mIPCs (E). (**F, G**) List of genes upregulated in hIPCs versus mIPCs that functionally fall under cell cycle, proliferation, and Wnt/Notch signalling, respectively (F) and the expression levels of selected genes (highlighted bold in the adjoining gene list) as identified in the individual samples of hIPCs versus mIPCs (G). ***P<0.001.

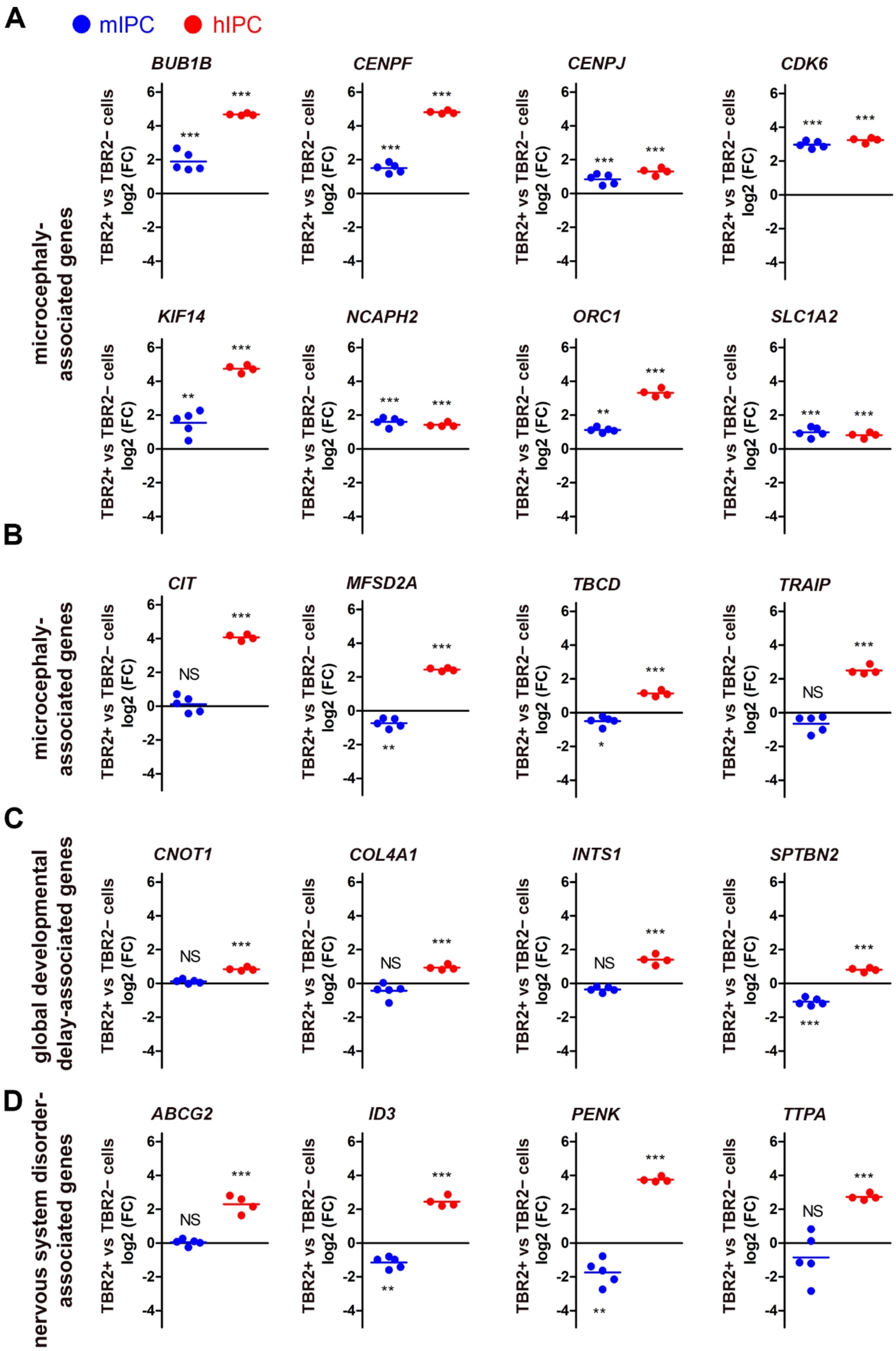
A
mIPC
hIPC
microcephaly-associated genes
BUB1B
CENPF
CENPJ
CDK6
KIF14
NCAPH2
ORC1
SLC1A2
B
microcephaly-associated genes
CIT
MFSD2A
TBCD
TRAIP
C
global developmental delay-associated genes
CNOT1
COL4A1
INTS1
SPTBN2
D
nervous system disorder-associated genes
ABCG2
ID3
PENK
TTPA
TBR2+ vs TBR2− cells
log2 (FC)
NS
***
**
*

Figure S3. hIPC genes involved in diseases linked to brain development.
(A-D) The expression levels of selected genes as identified in the individual samples in RNA-seq of mIPCs and hIPCs presenting evolutionary conserved IPC genes linked to primary microcephaly (A) and human-specific IPC-enriched genes associated with primary microcephaly (B), global developmental delay (C) and nervous system disorders (D). *N* = 5 RNA-seq libraries per condition for mIPCs and *N* = 4 RNA-seq libraries per condition for hIPCs. *P<0.05, **P<0.01, ***P<0.001; NS, not significant.

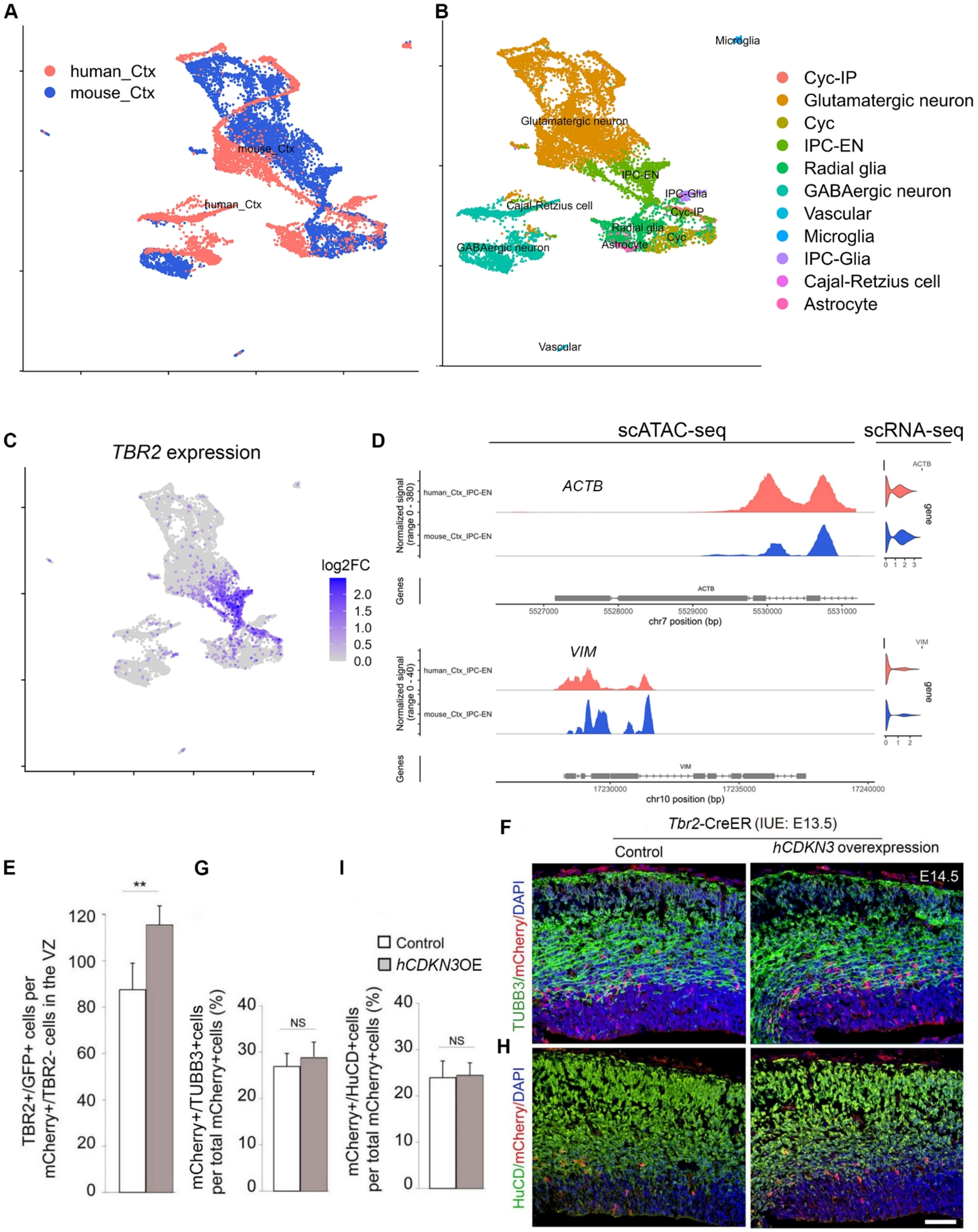

A
human_Ctx
mouse_Ctx
B
Microglia
Cyc-IP
Glutamatergic neuron
Cyc
IPC-EN
Radial glia
GABAergic neuron
Vascular
Microglia
IPC-Glia
Cajal-Retzius cell
Astrocyte
C
TBR2 expression
log2FC
2.0
1.5
1.0
0.5
0.0
D
scATAC-seq
scRNA-seq
ACTB
VIM
Normalized signal (range 0 - 380)
Normalized signal (range 0 - 40)
human_Ctx_IPC-EN
mouse_Ctx_IPC-EN
Genes
chr7 position (bp)
chr10 position (bp)
E
G
I
Control
hCDKN3OE
TBR2+/GFP+ cells per mCherry+/TBR2- cells in the VZ
mCherry+/TUBB3+cells per total mCherry+cells (%)
mCherry+/HuCD+cells per total mCherry+cells (%)
**
NS
NS
F
Tbr2-CreER (IUE: E13.5)
Control
hCDKN3 overexpression
E14.5
TUBB3/mCherry/DAPI
H
HuCD/mCherry/DAPI

Figure S4. Cross-species single-cell integration and effects of CDKN3 overexpression in mouse IPCs.

(A, B) UMAP visualization of integrated single-cell multiome datasets from developing human and mouse cortex (Wang et al., 2025; Liu et al., 2025), showing combined cells from both species following cross-species integration, colored by species (A) or by annotated cell clusters (B). **(C)** Feature plot showing *TBR2* expression across integrated clusters. **(D)** Cross-species single-cell ATAC-seq analysis showing chromatin accessibility at the *ACTB* and the *VIM* locus in mouse and human radial glia and IPC-EN together with expression levels. **(E)** Bar graph showing quantitative analysis of eGFP and mCherry co-expressing cells in E15.5 control and *hCDKN3*OE cortices (*N* = 6 images from 3 independent embryos per condition). **(F, H)** Double immunohistochemical analysis with antibodies that specifically mark mCherry and neuronal markers TUBB3 (F) or HuC/HuD (H) in the E14.5 *Tbr2*CreER mouse cortex, 24 h after electroporation with an empty mCherry-eGFP-only plasmid as control or *hCDKN3* overexpression plasmid. **(G, I)** Bar graphs showing quantitative analysis of TUBB3 (G) or HuC/HuD (I) and mCherry co-expressing neurons in E14.5 control and *hCDKN3*OE cortices (*N* = 6 images from 3 independent embryos per condition). Cyc-IP, cycling intermediate progenitor cells; IPC-EN, intermediate progenitors generating excitatory neurons; Cyc, cycling progenitors; IPC-Glia, intermediate progenitors generating glial cells. Scale bar: 100 μm. *P<0.05, **P<0.01, ***P<0.001; NS, not significant.

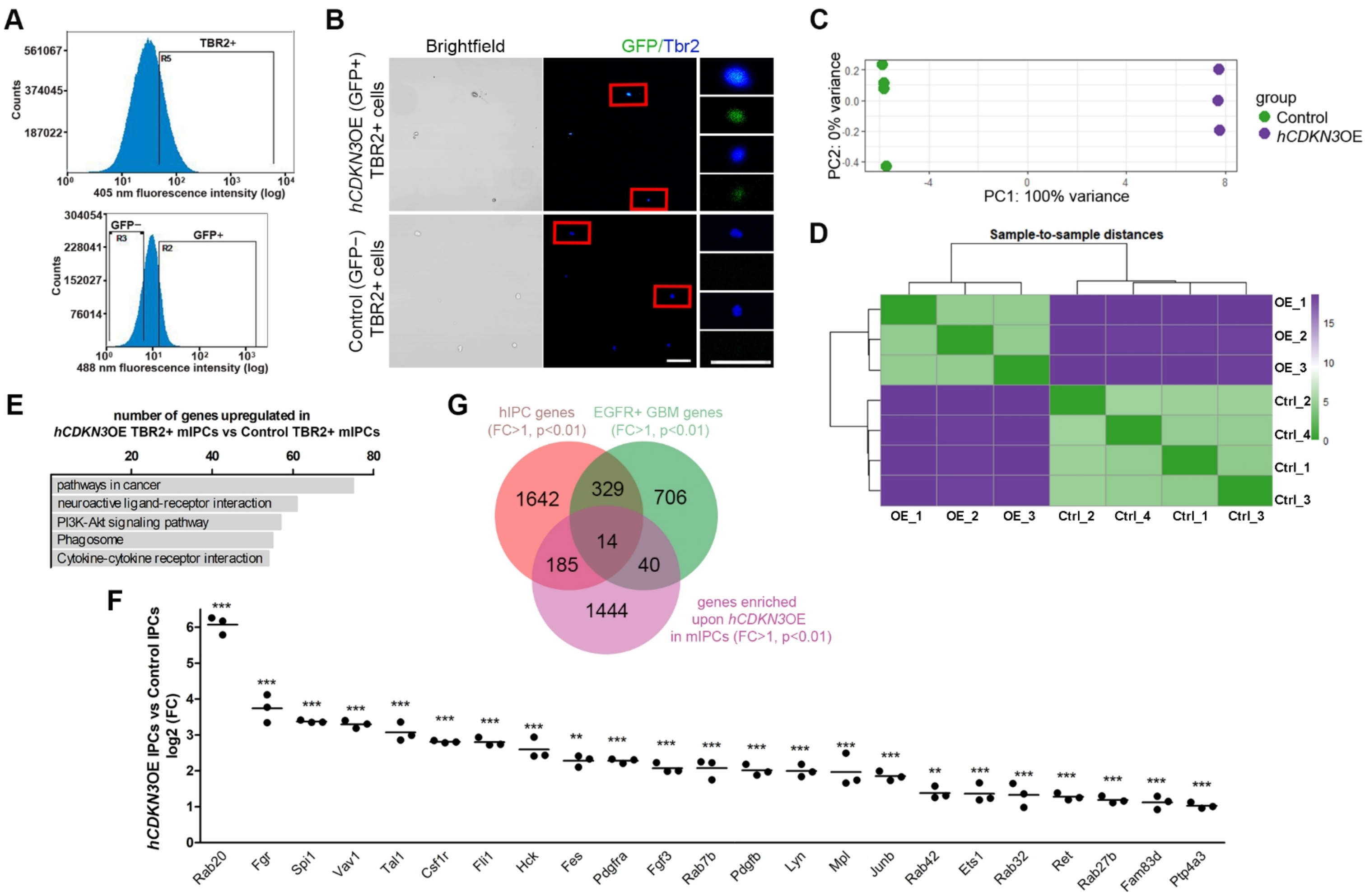

A
TBR2+
Counts
405 nm fluorescence intensity (log)
GFP−
GFP+
488 nm fluorescence intensity (log)
B
Brightfield
GFP/Tbr2
hCDKN3OE (GFP+) TBR2+ cells
Control (GFP−) TBR2+ cells
C
PC2: 0% variance
PC1: 100% variance
group
Control
hCDKN3OE
D
Sample-to-sample distances
OE_1
OE_2
OE_3
Ctrl_2
Ctrl_4
Ctrl_1
Ctrl_3
E
number of genes upregulated in
hCDKN3OE TBR2+ mIPCs vs Control TBR2+ mIPCs
pathways in cancer
neuroactive ligand-receptor interaction
PI3K-Akt signaling pathway
Phagosome
Cytokine-cytokine receptor interaction
G
hIPC genes (FC>1, p<0.01)
EGFR+ GBM genes (FC>1, p<0.01)
1642
329
706
14
185
40
1444
genes enriched upon hCDKN3OE in mIPCs (FC>1, p<0.01)
F
hCDKN3OE IPCs vs Control IPCs log2 (FC)
Rab20
Fgr
Spi1
Vav1
Tal1
Csf1r
Fli1
Hck
Fes
Pdgfra
Fgf3
Rab7b
Pdgfb
Lyn
Mpl
Junb
Rab42
Ets1
Rab32
Ret
Rab27b
Fam83d
Ptp4a3

Figure S5. Validation and transcriptomic analysis of CDKN3-overexpressing IPCs.

(**A**) Representative FACS plots showing sorting gates for TBR2 (405 nm) and GFP (488 nm) signals used to isolate *hCDKN3*-overexpressing (OE) and control TBR2+ IPCs (Ctrl). (**B**) Representative images of post-sorted cell suspensions from *in utero* electroporated mouse cortex labeled with TBR2 antibody. (**C**) PCA plot showing transcriptomic variance between *hCDKN3*OE and control samples. **(D)** Cluster dendrogram analysis of RNA-seq for sorted *hCDKN3*OE TBR2+ cells and control TBR2+ cells ($N \geq 3$ technical replicates for both conditions). **(E)** KEGG pathway enrichment analysis of genes upregulated in *hCDKN3*OE mIPCs vs control mIPCs (p-value < 0.01, log2FC > 1). **(F)** Expression levels of selected proto-oncogenes in *hCDKN3*OE mIPCs versus control mIPCs. **(G)** Overlap of genes upregulated in hIPCs, in EGFR+ GBM and in mIPCs with *hCDKN3*OE (p-value < 0.01, log2FC > 1). Scale bar: 100 μm. *P<0.05, **P<0.01, ***P<0.001; NS, not significant.

Supplemental Table Legends (separate files)

Table S1. Differential gene expression between human TBR2+ IPCs and TBR2− nuclei (related to Fig. 1 and S1)

Table S2. Overlap between mIPC and hIPC genes including Gene Ontology categories (related to Fig. 2 and S2)

Table S3. Chromatin, epigenetic and TF genes upregulated in hIPCs (related to Fig. 3)

Table S4. hIPC genes associated to diseases (related to Fig. 4 and S3)

Table S5. 266 human-specific top-enriched IPC genes and their categories (related to Fig. 5)

Table S6. Differential gene expression between *hCDKN3*OE mIPCs and Control mIPCs including Gene Ontology categories (related to Fig. 7 and S5)

Table S7. Statistical analyses

Table S8. List of primers
